# Decoding the fibromelanosis locus complex chromosomal rearrangement of black-bone chicken: genetic differentiation, selective sweeps and protein-coding changes in Kadaknath chicken

**DOI:** 10.3389/fgene.2023.1180658

**Published:** 2023-06-22

**Authors:** Sagar Sharad Shinde, Ashutosh Sharma, Nagarjun Vijay

**Affiliations:** Computational Evolutionary Genomics Lab, Department of Biological Sciences, IISER Bhopal, Bhauri, Madhya Pradesh, India

**Keywords:** Kadaknath, black-bone chicken, genetic linkage, fibromelanosis, *Fm* locus

## Abstract

Black-bone chicken (BBC) meat is popular for its distinctive taste and texture. A complex chromosomal rearrangement at the fibromelanosis (*Fm*) locus on the 20th chromosome results in increased endothelin-3 (*EDN3*) gene expression and is responsible for melanin hyperpigmentation in BBC. We use public long-read sequencing data of the Silkie breed to resolve high-confidence haplotypes at the *Fm* locus spanning both Dup1 and Dup2 regions and establish that the *Fm_2* scenario is correct of the three possible scenarios of the complex chromosomal rearrangement. The relationship between Chinese and Korean BBC breeds with Kadaknath native to India is underexplored. Our data from whole-genome re-sequencing establish that all BBC breeds, including Kadaknath, share the complex chromosomal rearrangement junctions at the fibromelanosis (*Fm*) locus. We also identify two *Fm* locus proximal regions (∼70 Kb and ∼300 Kb) with signatures of selection unique to Kadaknath. These regions harbor several genes with protein-coding changes, with the bactericidal/permeability-increasing-protein-like gene having two Kadaknath-specific changes within protein domains. Our results indicate that protein-coding changes in the bactericidal/permeability-increasing-protein-like gene hitchhiked with the *Fm* locus in Kadaknath due to close physical linkage. Identifying this *Fm* locus proximal selective sweep sheds light on the genetic distinctiveness of Kadaknath compared to other BBC.

## Introduction

Domestication of chicken during the Neolithic period involved a complex pattern of interbreeding with various jungle fowl species (Eriksson et al., 2008; Tixier-Boichard et al., 2011; Wang et al., 2020a; Lawal and Hanotte, 2021). After domestication, chickens have spread worldwide and occur as commercial, exotic, and indigenous village breeds. Humans use chickens as a research model due to their physiology and behavior (Cogburn et al., 2007), as game fowl and for religious reasons, or more commonly for egg or meat production (Rubin et al., 2010). Chicken is the most preferred source of meat for humans due to its easy availability and affordability (Kralik et al., 2016). Hence, understanding the genetics of meat features is commercially relevant (Mir et al., 2017). Although understudied, indigenous village chicken breeds with unique properties provide an opportunity to understand the genetics of meat. For instance, the black-bone chicken (BBC) is a delicacy due to its texture, color, firmness, flavor, and use in traditional medicine (Jaturasitha et al., 2008; Dou et al., 2022). The black color of the BBC results from melanin deposition throughout the body, i.e., melanin hyperpigmentation or fibromelanosis caused by the *Fm* allele (Muroya et al., 2000). Bateson and Punnet were the pioneers in identifying the autosomal dominant *Fm* allele (Bateson et al., 1911). Modern studies found a chromosomal rearrangement on chromosome 20 is involved in the *Fm* locus (Dorshorst et al., 2011; Dharmayanthi et al., 2017; Sohn et al., 2018). The overexpression of the endothelin-3 (*EDN3*) gene located within the *Fm* locus is responsible for hyperpigmentation seen in the BBC (Dorshorst et al., 2011; Shinomiya et al., 2012; Zhang et al., 2022).

BBC breeds have anti-fatigue and anti-hypoxic abilities, with their meat having antioxidant properties (Tu et al., 2009; Dou et al., 2022), high carnosine (Tian et al., 2007; Sharma et al., 2022b), and lower fat and cholesterol content (Jaturasitha et al., 2008; Tian et al., 2011). Some BBC breeds also have local adaptations. For instance, the Korean Ogye has improved fetal viability and innate immunity against microbial and viral infections (Cho et al., 2022). BBC breeds occur globally and have distinct names, such as Ayam Cemani (Indonesia), Black H'Mong (Vietnam), Tuzo (Argentina), Svarthöna (Sweden) (Johansson and Nelson, 2015; Dharmayanthi et al., 2017), Yeonsan Ogye (Korea) (Sohn et al., 2018), and Thai BBC (Thailand) (Buranawit et al., 2016). China has a high diversity of BBC breeds, including Silkie, Jiangshan, Lueyang, Sichuan, Xingwen, Yugan, Dehua, Jinhu, Muchuan, Wumeng, Yanjin, Xichuan, Tuanfu, Wuliangshan, Emei, and Miyi fowl (Zhu et al., 2014; Huang et al., 2020b; Li et al., 2020; Kriangwanich et al., 2021; Dou et al., 2022). India has a single breed of BBC, commonly known as Kadaknath (Sharma et al., 2022b).

Kadaknath is considered native to the Jhabua, Alirajpur, and Dhar districts of Madhya Pradesh, but its farming has recently spread across India (Jadhao et al., 2022; Tripathi et al., 2022). The Kadaknath breed has long been documented as a distinctive indigenous Indian breed and is also called Kali Masi or Karaknath (Slater, 1945). At least three distinctive phenotypes (i.e., jetblack, pencil, and golden) occur within the Kadaknath breed (Haunshi and Prince, 2021). In jetblack, all the body parts like plumage, comb, internal organs, eyes, skin, beak, shank, and claw are entirely black, whereas pencil and golden have white and golden color patches on the plumage, respectively (Haunshi and Prince, 2021). Several unique characteristics, such as earlier egg-laying maturity, high-protein content, better disease resistance, and adaptation to the local environment, are attributed to the Kadaknath chicken (Rout et al., 1992; Haunshi et al., 2011; 2022; Thakur et al., 2015; Jena et al., 2018; Shanmathy et al., 2018; Sahu et al., 2019; Haunshi and Prince, 2021; Sehrawat et al., 2021; Sharma et al., 2022b; Jadhao et al., 2022). Understanding the genetics behind these traits will help establish the uniqueness of Kadaknath and guide de-extinction efforts and breeding programs.

Despite its immense popularity and commercial value, the genomics of the Kadaknath chicken breed has received limited attention. Therefore, further research and genomic analyses are required to understand adaptations in this breed and its genetic history. The aims of this study are as follows: (1) Performing whole-genome re-sequencing of Kadaknath and evaluating its relationship with other black-bone and non-black-bone chicken breeds. (2) Assessing whether Kadaknath and other BBC breeds share a common origin for the *Fm* locus by comparing the chromosomal rearrangement junction and resolving the correct arrangement of duplicated regions at the *Fm* locus. (3) Evaluating how the BBC breeds dispersed to various parts of Asia. (4) Using population genetic statistics to identify signatures of selection in Kadaknath compared to other BBC breeds.

## Materials and methods

### Population sampling

The study was approved by the Institutional Ethics Committee (IEC) of the Indian Institute of Science Education and Research, Bhopal, vide reference number IISERB/IEC/Certificate/2018-11/03 dated 8th June 2018. We purchased the meat of six individuals (two individuals each from jetblack, pencil, and golden morphs (Supplementary Figure S1)) of Kadaknath from an Food Safety and Standards Authority of India (FSSAI)-licensed shop in Bhopal, Madhya Pradesh, India. We procured two other individuals with a black-bone phenotype from the same FSSAI-licensed shop to examine hybrids. One had a golden–pencil external appearance, and another was completely white on the exterior. We also sampled two non-BBCs from the same shop to determine the genetic relationship of Kadaknath with the native-village chicken and broiler reared in the same poultry (Supplementary Figure S1). To reduce the chances of inter-breeding and have relatively pure Kadaknath samples, we obtained three (one individual each from jetblack, pencil, and golden morphs) additional Kadaknath breed chickens from Jhabua, Madhya Pradesh. Whole-genomic DNA with high purity and quality was extracted from the liver tissue samples using DNeasy Blood & Tissue Kits (QIAGEN). We generated >25x coverage whole-genome short-read paired-end data (using Illumina Novaseq) for all 13 individuals sampled (Supplementary Table S1).

To evaluate the relationship of Kadaknath chicken globally, we compared our dataset with publicly available chicken re-sequencing data. High-quality re-sequencing datasets of 88 chicken individuals from other BBCs, commercial chicken lines, and other distinctive chicken breeds with a coverage >20x were selected and obtained from European Nucleotide Archive (ENA) and Korean National Agricultural Biotechnology Information Center (KNABIC) (https://nabic.rda.go.kr/). Out of 88 individuals, 23 are BBCs, which we considered for further analysis. Hence, we analyze a dataset of 101 individuals (88 public +13 sequenced as part of this study) from different breeds (Supplementary Table S2 for more details) (Fan et al., 2013; Roux et al., 2014; Wang et al., 2015; Ulfah et al., 2016; Sohn et al., 2018; Qanbari et al., 2019; Li et al., 2020; Mariadassou et al., 2021; Cho et al., 2022). Transcriptomic datasets from four individuals each for five native Indian breeds (Kadaknath, Ankleshwar, Aseel, Punjab brown, and Nicobari) and broiler chicken from India generated from the breast muscle are publicly available (Supplementary Table S2). We only include these 24 transcriptome samples with the 101 WGS samples to assess the population structure.

### Read mapping, variant calling, and phylogeny

We mapped the paired-end raw reads of 101 individuals to the chicken genome assembly (genome assembly version is Gallus_gallus.GRCg6a) using the BWA (Li and Durbin, 2009) (Burrows–Wheeler aligner) v0.7.17-r1188 mem read mapper with default parameters. We added the read group information using Picard tools and removed duplicate reads in all 101 individual BAM files (https://github.com/broadinstitute/picard). We performed the variant calling using FreeBayes (Garrison and Marth, 2012) with different quality control flags such as -min-alternate-count -C 10, -min-mapping-quality -m 20, -min-base-quality -q 20, and -min-coverage 10. Similarly, we used the bcftools (Li and Barrett, 2011; Danecek et al., 2021) with mapping quality flags such as -mapping quality -C 50, -min base quality -Q 20, and -min mapping quality -q 20 for robust variant calling. We removed the indels from variant calls using VCFtools (Danecek et al., 2011) with the -remove-indels flag and extracted the common SNPs using both SNP callers for retaining reliable SNP calls. The single-nucleotide polymorphisms (SNPs) identified by both variant callers (bcftools v1.9 and FreeBayes v1.0.0) were used for subsequent analysis. To identify the effect of genetic variants, we used the snpEff v SnpEff 4.3t and GRCg6a.96″databases for annotation (Cingolani et al., 2012). For the prediction of the possible impact of non-synonymous fixed variants in BPIL on the protein’s structure and function, we used the Polymorphism Phenotyping v2 (PolyPhen-2) tool (Adzhubei et al., 2010). Common SNPs from both vcf files were used to construct phylogeny for 34 and 101 individuals using SNPhylo (Lee et al., 2014) v20180901, based on a maximum likelihood tree with 1,000 bootstrap values. We excluded the scaffolds, Z, W, and MT chromosomes in phylogeny analysis. The local phylogeny for Dup1 and Dup2 regions was generated using the vk phylo command (using both NJ and UPGMA methods) implemented in the VCF-kit (Cook and Andersen, 2017). The Mt genome haplotype median-joining networks were constructed using PopART (Leigh and Bryant, 2015) and SplitsTree (Huson, 1998).

We mapped the paired-end transcriptomic raw read data of 24 individuals to chicken genome assembly (genome assembly version is Gallus_gallus.GRCg6a) using the STAR (Dobin et al., 2013) (v2.7.0d read mapper with default parameters. We perform the variant calling using bcftools with flags -mapping quality -C 50, -min base quality -Q 20, and -min mapping quality -q 20. Using VCFtools, we extracted 67,617 SNPs only considering autosomes where no data were missing in any individuals using the flag–max-missing 1. For assessing the population structure of all 125 individuals (101 with whole genomic data +24 transcriptomic data), we performed the variant calling only on 67,617 SNPs (extracted from transcriptomic data of 24 individuals) using bcftools.

### *Fm* locus junction identification

The *Fm* locus consists of a complex chromosomal rearrangement composed of two different non-paralogous regions (Dup1 (∼127 Kb) and Dup2 (∼170 Kb)) separated by an intermediate (Int) region. Dup1 and Dup2 regions are both duplicated and are involved in a complex rearrangement consisting of two junctions: (**A**) Dup1 + (inverted Dup2) and (**B**) (inverted Dup1) + Dup2. To identify the base-pair level positions of Dup1, Dup2, and Int regions, we compared the short-read coverage of black and non-black chicken in 1 Kb windows along chromosome 20. We used the makewindows command of bedtools (Quinlan and Hall, 2010) (v2.26.0) to create 1 Kb non-overlapping windows along chromosome 20. The number of reads in each 1 Kb window was calculated using the bedtools coverage command. We shortlisted adjacent windows with drastically different read coverage in black individuals but not in non-black individuals. The base-pair level coordinates of Dup1 and Dup2 in the Gallus_gallus.GRCg6a genome were narrowed down further using coverage estimates in 1bp windows. Dup1 starts at 20:10766772 and ends at 20:10894151. Dup2 occurs further along the chromosome and starts at 20:11306686 and ends at 20:11477501. In addition to Dup1, Dup2, and Int regions, we defined ∼500 Kb flanking regions as Flank1 (20:10263555-10766771) and Flank2 (20:11477502-11980000).

### Black-bone-specific *Fm* locus junction

The junction between the rearranged regions in the BBC does not occur in the Gallus_gallus.GRCg6a genome (Supplementary Figure S2, 3). In searching for a completely assembled *Fm* locus, we check the previously published assembly of BBC breeds. We find that the two genome assemblies of Silkie (Silkie2 (GCA_024679325.10 and Silkie3 (GCA_024653025.1) generated by Li et al. (2022b) and one genome assembly of Yeonsan Ogye generated by Sohn et al. (2018) have been published. We generated the dot plot of chromosome 20 of chicken genome assembly (Gallus_gallus.GRCg6a) with chromosome 20 of Yeonsan Ogye (CM008847.1) and Silkie3 (CM045235.1) using the Gepard tool (Krumsiek et al., 2007). The rearranged *Fm* locus is partially assembled at Chr 20 in Yeonsan Ogye (Supplementary Figure S2, 3). In the Silkie3 genome, parts of the *Fm* locus occur on chr20 and unplaced scaffold (JAJMOI010001544.1) (Supplementary Figure S4, 5). However, in Silkie2 genome assembly, chr20 is not assembled, so Dup1 and Dup2 are independently present on four different unplaced scaffolds (JAJMOM010018106.1, JAJMOM010006651.1, JAJMOM010000156.1, and JAJMOM010000154.1). None of the existing assemblies provide the full-length assembly of the *Fm* locus in BBC breeds. While the junction sequences have been reported earlier (Dorshorst et al., 2010) for the Silkie breed, it is unclear whether all BBC breeds share the same junction sequences. Hence, we searched for the *Fm* locus junction in the Korean BBC PacBio data from ENA (SRR6189090). Based on our search of PacBio reads that mapped to the Gallus_gallus.GRCg6a genome, we shortlisted reads that simultaneously aligned to two of the five (Flank1, Dup1, Int, Dup2, and Flank2) genomic regions we have defined. We found several reads spanning Flank1-Dup1, Dup1-Int, Int-Dup2, and Dup2-Flank2, which is unsurprising because these regions are adjacent.

We found five reads (SRR6189090.111279, SRR6189090.56386, SRR6189090.880702, and SRR6189090.387043, and SRR6189090.54824) that span across both Dup1 and Dup2. Dup1 and Dup2 are far apart in their genomic location in Gallus_gallus.GRCg6a assembly. Hence, these reads support a rearrangement that leads to junctions between these two regions. Three of these five reads (SRR6189090.111279, SRR6189090.880702, and SRR6189090.387043) support the junction Dup1 + (Inverted Dup2) (i.e., START-DUP1-END-END-DUP2-START), and the other two reads (SRR6189090.54824 and SRR6189090.56386) support the junction (inverted Dup1) + Dup2 (i.e., END-DUP1-START-START-DUP2-END). The read coverage at these junctions consistently differed between all black-bone and non-black-bone chickens. Based on the read coverage across the Fm locus junctions, we identified 34 out of 101 samples analyzed as BBC.

### Long-read data mapping and haplotype-specific read identification

Recently, a new high-coverage multi-platform genomic public dataset for the Silkie BBC became available on the European Nucleotide Archive (ENA) as part of the Bioproject# PRJNA805080 (we thank China Agricultural University for publishing these data). With a ∼65X (Nanopore) and >660X coverage (PacBio) of the same Silkie individual, this dataset is well suited to resolve the haplotypes at the *Fm* locus and identify the exact order of rearrangement. For mapping the PacBio and Nanopore long-read data of this Silkie individual, we used the Gallus_gallus.GRCg6a (galgal6a genome assembly) genome with the BWA (Burrows–Wheeler aligner) v0.7.17-r1188 bwasw read mapper with the flags -t 24, -a2, -b3, -q2, -r2, and -z1 to obtain high-quality read mapping.

We used the bam-readcount (Khanna et al., 2022) tool to obtain the number of reads supporting each of the four nucleotide bases at each position along the Dup1 (∼127 Kb) and Dup2 (∼170 Kb) regions. Potentially heterozygous sites with reliable read support were shortlisted as sites with at least 10 reads supporting each of the two alleles. Sites with at least 10 reads supporting three or more bases were excluded as potentially tri-allelic sites. Using these criteria, we found around 1% of the sites that are potentially heterozygous in Dup1 (1,214/127000) and Dup2 (1774/170000) regions. Subsequent steps were performed using the phasing.sh script (provided on our GitHub page). For each of these 2,988 sites, we labeled the reads based on the nucleotide base (i.e., A, T, G, and C) present in that read at each position using the biostar214299 program from jVarkit (Lindenbaum, 2015). We prepared the list of reads supporting each of the four bases for each site using the reads labeled in the previous step. Starting from the sites (i.e., 10766895 for Dup1 and 11476819 for Dup2) identified by visual inspection of the long-read alignments in IGV, we extended the haplotypes using the phasing.sh script. The rationale of the script is to find the next site which can distinguish the haplotypes with at least five reads supporting each haplotype. The number of reads supporting each of the four nucleotide bases at the two sites is calculated for every pair of sites the script considers. Hence, the number of reads supporting each of the 16 possible combinations of the four bases at the two sites is counted. We used this script sequentially to extend the haplotypes by identifying haplotype-defining sites by manually inspecting these counts. Of the 2,988 sites considered, 49 haplotype-defining sites were sufficient to span entire Dup1 and Dup2 regions (Supplementary Table S3).

### Principal component analysis and admixture

Principal components analysis (PCA) was performed using PCAngsd (Meisner and Albrechtsen, 2018) based on genotype likelihood estimates from Analysis of Next-Generation Sequencing Data (ANGSD) v0.935 (Korneliussen et al., 2014). PCA was performed both genome-wide and *Fm* locus region-wise (Flank1, Dup1, Int, Dup2, and Flank2) for the 34 black-bone and all 101 chicken individuals**.** We used several flags in ANGSD for population structure analysis as follows: -GL 2, -doMaf 1, -minMapQ 30, -minQ 20, -doGlf 2, and -SNP_pval < 1e-6. Genotype likelihood values from ANGSD were used to identify principal components using PCAngsd and genome-wide admixture proportions using NGSadmix (Skotte et al., 2013). NGSadmix was run for different values of K from K1 to K10 using each K with 15 iterations with flags -minMaf 0.05 and -minInd as specified. Admixture analysis suggests Kadaknath has a sub-structure but remains distinct from the other black-bone breeds at best K = 7 using median values of Ln (Pr Data) or log probability of the data (referred to as ln Pr (X|K), i.e., k for which Pr(K = k) is highest, while according to ΔK (Evanno’s best K method), the best K is 8. We used the Evanno (Evanno et al., 2005) method implemented in the Clumpak (Kopelman et al., 2015) web server to find the best K.

Using the variant calls of 125 individuals for 67,617 SNPs, we performed the admixture analysis through AdmixPipe v3 (Mussmann et al., 2020) for K1– K15, and we ran 10 iterations for each K. During the admixture analysis, two individuals of the Brown layer (BROL1 and BROL2, Supplementary Table S2) got excluded due to the poor quality of variant calls. In the admixture analysis of 123 individuals, we found that according to Evanno’s best K method, the best K is 2. The observation of K = 2 in admixture analysis using ΔK (Evanno’s best K method) is reported in more than 50% of recent studies (Janes et al., 2017). Hence, we followed the recommendations of Gilbert et al., (2012) and Janes et al. (2017) to assess the population structure. We re-run the admixture pipeline for 109, 100, and 88 individuals after removing the populations (Rhode Island Red, White Leghorn, and broiler), respectively, which separated at K = 2. As per the suggestion by Janes et al. (2017) to infer the optimal K value, we are providing the Ln Pr (X|K) and ΔK plots and bar plots for the multiple K values in supplementary. PCA was also performed using PCAngsd for *Fm* locus region-wise (Flank1, Dup1, Int, Dup2, and Flank2) using 123 individuals (BROL1 and BROL2 are excluded).

### Population genetic analysis

Chinese black-bone breeds have diverged to differing extents from Kadaknath and have limited sample sizes. Hence, we combined the individuals from closely related XBBC (Xichuan black-bone chicken), LCEM (Emei black fowl), and LCMY (Miyi black fowl) breeds into a single population representative of Chinese black-bone (CHIN, n = 9) chicken and JETB, PENC, and GOLD into another population representative of Kadaknath (KADK, n = 9) (Supplementary Table S4). We calculated population genetic statistics genome-wide to identify signatures of selection. However, to avoid false positives, we excluded genomic regions (50 Kb windows with <80 percent callable sites) with poor callability. We used the CallableLoci walker of GATK (McKenna et al., 2010) on the BAM files to quantify callability. For identifying callable regions from mapped BAM files for CHIN and KADK individuals, we used GATK with different flags such as -minMappingQuality 20, -minBaseQuality 20, -minDepth 10, -minDepthForLowMAPQ 20, and -maxFractionOfReadsWithLowMAPQ 20. Furthermore, we filtered 50 Kb windows with >0.1 repeat element fraction to rule out the possibility of false positives (Supplementary Figure S6–8). We used stringent coverage and quality criteria (-GL 2, -dosaf 1, -baq 1, -C 50, -setMinDepthInd 6, -minInd 3, 4 or 9, -minMapQ 30, -minQ 20, and -doCounts 1) in ANGSD to calculate all population genetic statistics. The list of individuals in each population and the population pairs compared is in Supplementary Table S4. We found that at least 20,000 of the 21,659 50 Kb windows had sufficient high-quality data in all populations.

We used the folded site frequency spectrum (SFS) approach implemented in ANGSD to calculate genome-wide population-specific estimates of π (the average pairwise differences), Watterson’s θ (the average number of segregating sites), τ (Tajima’s D), and Fu and Li’s D. We estimated inter-population genomic differentiation (F_ST_) and divergence (D_xy_) using ANGSD and popgenWindows python script (https://github.com/simonhmartin/genomics_general) for each population pair. The F_ST_ estimates from the two methods were highly correlated (Pearson’s *ρ* = 0.98, *p*-value <2.2 e−16) (Supplementary Figures S9,10). We defined FST outlier regions as 50 Kb windows in the top 1 percent of the genome-wide estimates and merged adjoining windows using bedtools. Similarly, the 50 Kb windows in the top 10 percent of the genome-wide estimates of Dxy were deemed to have high levels of divergence. We identified fixed sites as those sites with FST >0.9. We estimate haplotype-based statistics iHS and XP-EHH in the rehh (Gautier and Vitalis, 2012) R package with option polarized = FALSE using genotype data phased with SHAPEIT (Delaneau et al., 2011).

## Results

### Whole-genome re-sequencing

**FIGURE 1:**
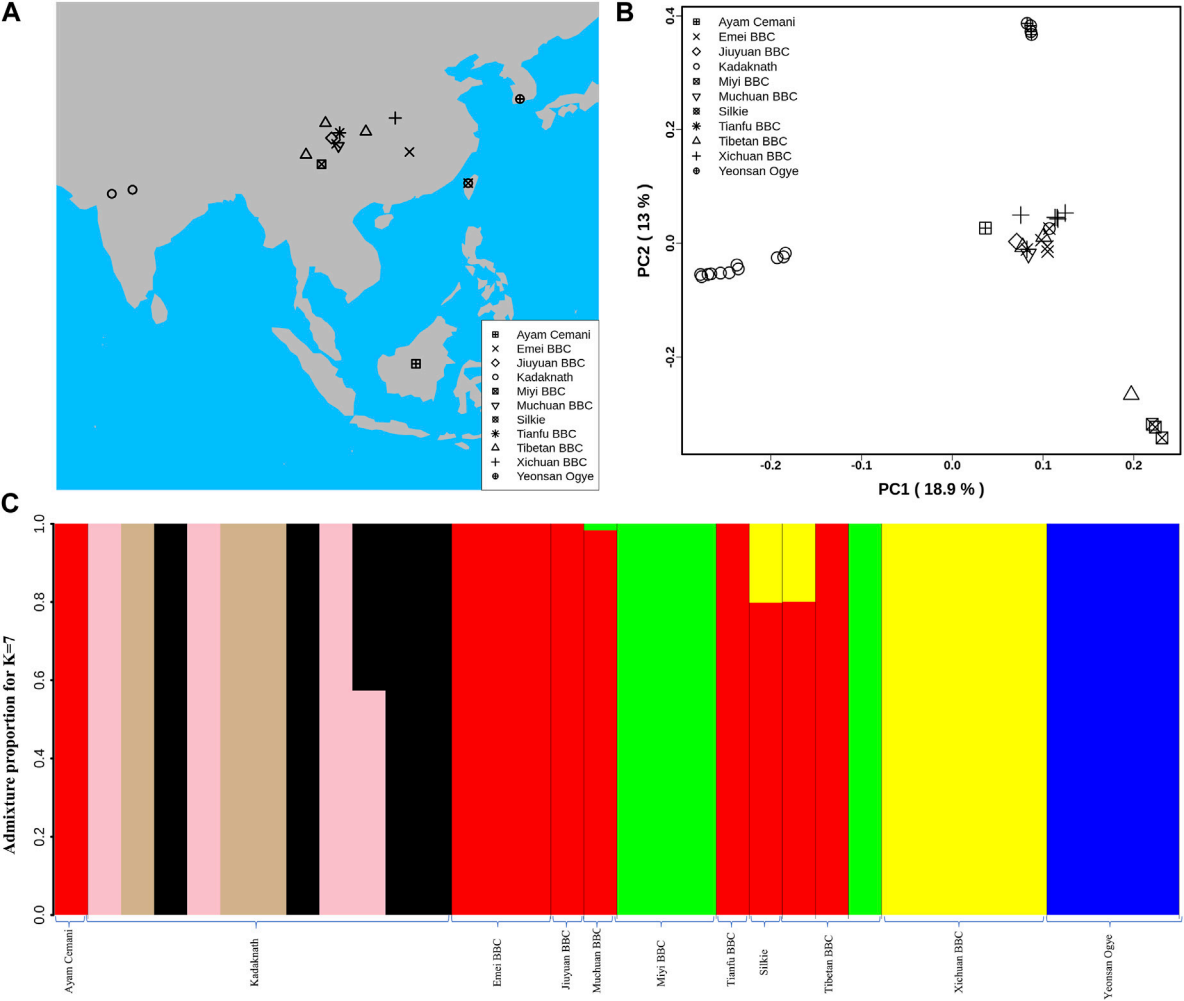
Population structure analysis: **(A)** Geographical locations of different BBC breeds used in this study are shown on the map using different shapes. The map was generated using rworldmap, map, and mapdata R packages. **(B)** Genome-wide principal component analysis reveals the genetic relationship of 34 BBC individuals. PC1 and PC2 explained 18.9% and 13% variance, respectively. **(C)** Population genetic structure and individual ancestry were estimated using NGSadmix for 34 BBCs from different breeds based on best K = 7.

We generated >25X coverage whole-genome re-sequencing Illumina data for 13 chicken individuals from India. Our collection includes nine Kadaknath samples as representatives of all three extant morphs jetblack (JETB n = 3), golden (GOLD *n* = 3), and pencil (PENC n = 3). We also sampled one individual with a golden–pencil-like phenotype (GOPE n = 1), one with complete white plumage with black bone (CROS n = 1), one non-black-bone individual where plumage was black (NONB), and one individual of broiler breed (Supplementary Figure S1; Supplementary Table S1). We compared these Kadaknath chicken genomes with public re-sequencing datasets of chicken breeds. The sampling location (Figure 1A for black-bone chicken) of individuals analyzed in this study is spread across Asia (for more detail, see Supplementary Table S2, which includes the complete list of all chicken breeds examined). A detailed map of China depicts the locations of all the Chinese black-bone breeds (Supplementary Figure S11).

### Kadaknath is a distinct breed of black-bone fowl

The major axes of genetic variation in the BBC (PC1:18.9% and PC2:13%) separated the Kadaknath, Yeonsan Ogye (YOSK n = 4), and Chinese BBC (*n* = 17) (see Figure 1B; Supplementary Figure S12). We observed that all Kadaknath individuals form a single cluster distinct from other black-bone breeds (from Indonesia, China, and South Korea) in the PCA (Figure 1B; Supplementary Figure S12). Similarly, in the genome-wide PCA analysis of 101 individuals consisting of both BBCs and non-BBCs, Kadaknath forms a separate cluster from other commercial, native, and BBC breeds in different PC comparisons (Supplementary Figure S13A–C).

Admixture analysis suggests that Kadaknath has sub-structure but remains distinct from the other black-bone breeds at best K = 7 (see Figure 1C and Supplementary Figure S14A–C; Supplementary Figure S15). In Chinese BBC, the admixture analysis identified three groups supporting the PCA clustering pattern, while YOSK forms a separate cluster (see Figure 1C; Supplementary Figure S12). We observed lower genetic diversity (*π* = 0.002, θ = 0.002) in YOSK (*n* = 4), while Kadaknath (*n* = 9) and CHIN (*n* = 9) BBC have comparable genetic diversity (*π* = 0.004 and 0.003, θ = 0.003 in both) (Supplementary Figure S16A, B). Genetic diversity can be affected by crossbreeding with native chicken breeds. Genome-wide admixture analysis of 101 individuals consisting of both BBCs and non-BBCs supports gene flow between Chinese BBC and native Chinese non-BBC breeds (Supplementary Figure S17A–C). Crossbreeding between Ogye and broiler is also supported by the genome-wide admixture analysis. Including native Indian non-BBC breeds in the admixture analysis suggests crossbreeding between Kadaknath and Ankleshwar breeds (Supplementary Figure S18A–C). However, Kadaknath is distinct from other native Indian breeds, and the Kadaknath samples from the transcriptome dataset cluster with the genome sequencing data generated in this study (Supplementary Figure S19–21A–C).

In contrast to the nuclear genome, the mitochondrial genome haplotype network did not separate Kadaknath from other BBC breeds (Supplementary Figure S22, 23). The *Fm* locus region on chromosome 20, which codes for the black-bone phenotype, is the defining feature of all BBCs. Even after excluding chromosome 20, the PCA of the remaining chromosomes finds that Kadaknath is genetically distinct from other BBC breeds (Supplementary Figure S24A–C). Hence, the genetic distinctiveness of Kadaknath is spread across the entire genome.

### *Fm_2 is the correct arrangement of duplicated regions at the *Fm* locus

**FIGURE 2:**
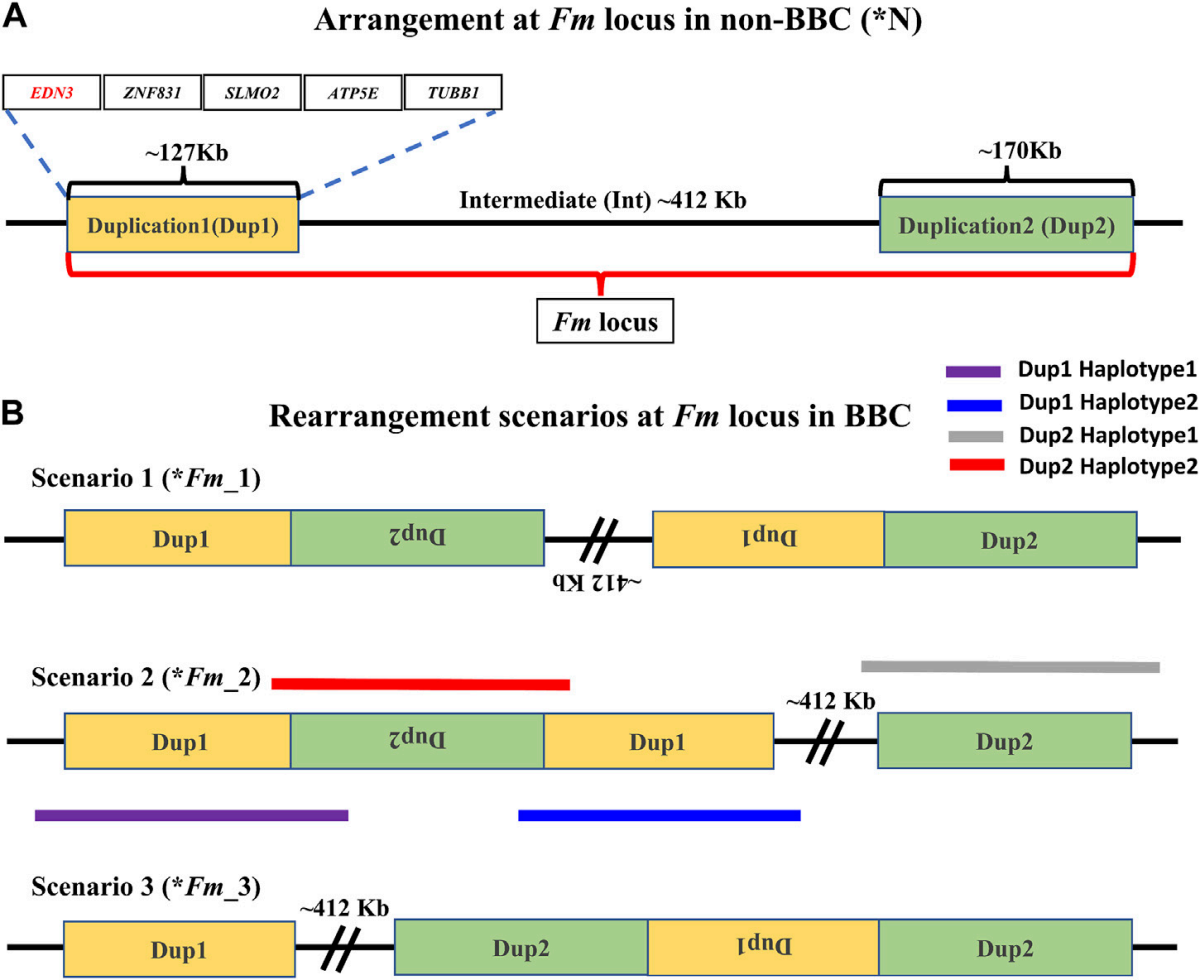
Arrangement of the *Fm* locus region in non-BBC and BBC breeds: two different non-paralogous regions on chromosome 20 are referred to as Duplication 1 (Dup1) and Duplication 2 (Dup2), shown in gold and light green colors, respectively. The length of the Dup1 region is ∼127 Kb, the intermediate (Int) is ∼412 Kb, and Dup2 is ∼170 Kb in size. **(A)** These regions are neither duplicated nor rearranged in non-BBC (*N). Dup1, Int, and Dup2 regions are collectively referred to as the *Fm* locus. Dup1 contains five genes, *EDN3*, *ZNF831*, *SLMO2*, *ATP5E*, and *TUBB1*, whereas the Dup2 region does not have any protein-coding genes. **(B)** Three possible scenarios (*Fm_1, *Fm_2, and *Fm_3) for *Fm* locus have been proposed in the BBC (earlier described in Dorshorst et al., 2011; Dharmayanthi et al., 2017; Sohn et al., 2018). The dark solid purple line represents haplotype-1, which spans across Dup1 from Flank1 to inverted Dup2 (i.e., Flank1 + Dup1 + (inverted Dup2)). The blue line represents haplotype-2, which spans Dup1 from inverted Dup2 to Int (i.e., (inverted Dup2) + Dup1 + Int).

In the non-black chicken, Dup1 (∼127 Kb), Int (∼412 Kb), and Dup2 (∼170 Kb) regions occur in a single copy, are arranged sequentially, and are flanked by Flank1 (∼500 Kb) and Flank2 (∼500 Kb) regions (Figure 2A). While the Dup1 region contains five protein-coding genes (*EDN3*, *ZNF831*, *SLMO2*, *ATP5E*, and *TUBB1*), the Dup2 region probably consists of only long-non-coding RNA genes. All non-BBCs have a single copy of this region, referred to as *N locus (Figure 2A). The corresponding locus in the BBC is known as the *Fm* locus. The *Fm* locus consists of three different non-paralogous regions (Dup1, Int, and Dup2) that form a complex chromosomal rearrangement in which both Dup1 and Dup2 regions are duplicated, giving rise to two junctions: (**A**) Dup1 + (inverted Dup2) and (**B**) (inverted Dup1) + Dup2. Although the exact ordering of these regions in the rearrangement is not conclusively established, both Dup1 and Dup2 regions are known to be duplicated due to these regions having a sequencing coverage that is twice the genomic average (Dorshorst et al., 2011). The presence of Dup1 + (inverted Dup2) and (inverted Dup1) + Dup2 junctions has been verified in several BBC breeds (Dorshorst et al., 2011; Dharmayanthi et al., 2017; Sohn et al., 2018). Based on this information, three possible scenarios have been proposed by earlier studies (Figure 2B). The *Fm_2 scenario is supported based on crosses between black and non-black-bone chicken (Dorshorst et al., 2011). However, both *Fm_2 and *Fm_3 scenarios require two rearrangement events compared to a single rearrangement event needed for the *Fm_1 scenario (Sohn et al., 2018).

Distinguishing between these three (*Fm_1, *Fm_2, and *Fm_3) scenarios requires long-range connectivity information such as long-read sequencing data (PacBio, Nanopore, Synthetic long reads, *etc.*), Hi-C (high-resolution chromosome conformation capture) (Kronenberg et al., 2021; Wang et al., 2022), or optical mapping (Weissensteiner et al., 2020). Both *Fm_1 and *Fm_2 contain the same adjacent regions for Dup1 (defined as haplotype-1 and haplotype-2 in Figure 2B) inconsistent with *Fm_3. Hence, long-range information that can span the entire ∼127 Kb Dup1 region in a single read will be able to distinguish between (*Fm_1 and *Fm_2) vs. *Fm_3. However, to differentiate between *Fm_1 and *Fm_2 scenarios, we must span the entire ∼170 Kb Dup2 region in a single read. Spanning Dup1 or Dup2 regions with PacBio (average read lengths of 10–25 Kb) or Nanopore (average read lengths of 10–30 Kb) technologies with a single read is challenging but not impossible as reads longer than 1 Mb are possible (Amarasinghe et al., 2020; Hon et al., 2020). Without such individual reads that can span the entire region, it is possible to perform read-based phasing to infer haplotypes that extend to distinct junctions (Patterson et al., 2015). Using a public long-read dataset, we have inferred distinct high-confidence Dup1 and Dup2 haplotypes (supporting the *Fm_2 scenario).

**FIGURE 3:**
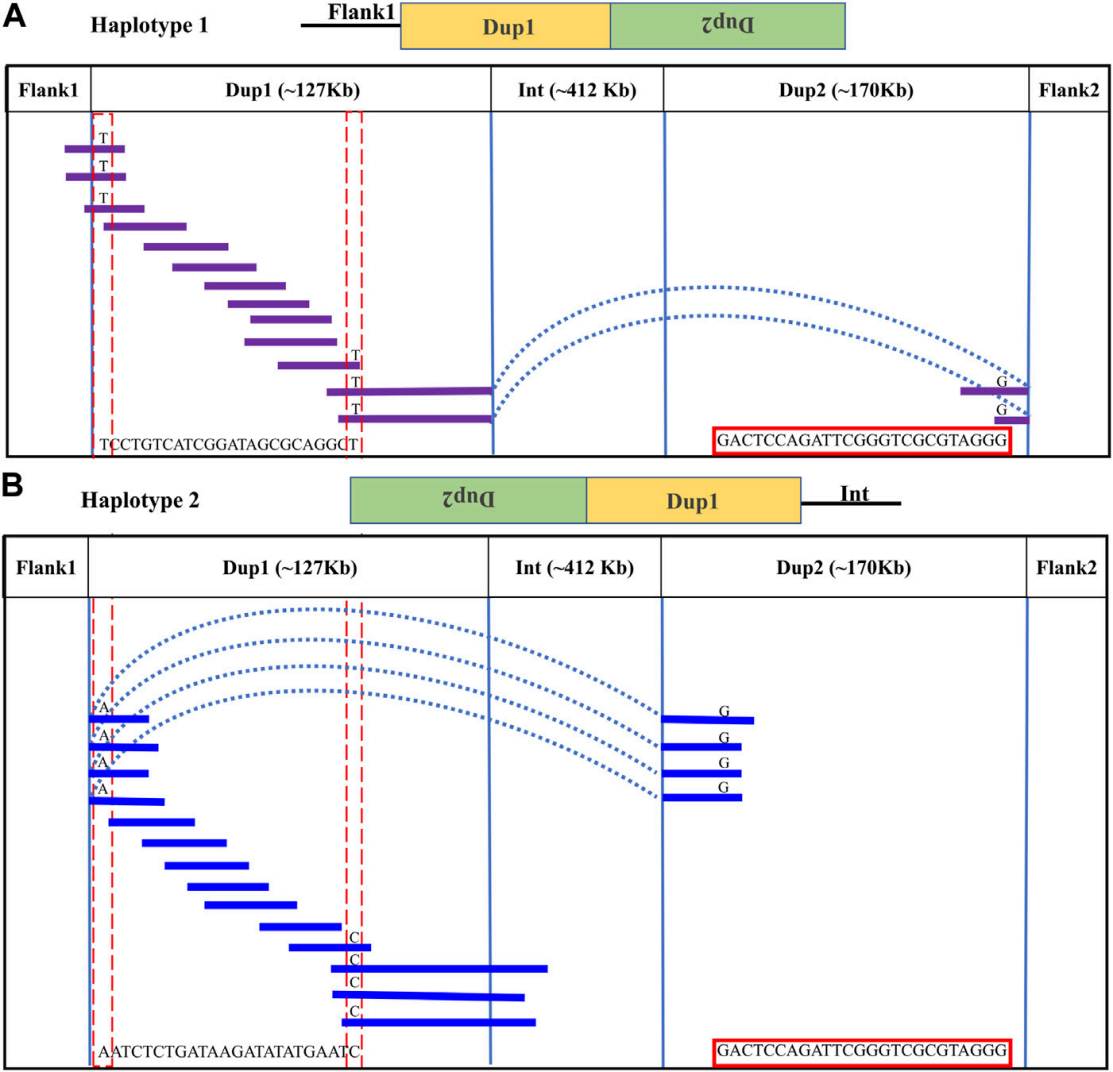
Long-read-based haplotypes resolve the sequence spanning the Dup1 region at *Fm* locus: the two haplotypes spanning the Dup1 region are distinguished by distinct alleles at 24 positions using long sequencing reads. Red dotted vertical boxes highlight the alleles that differ between haplotype-1 and haplotype-2 at the same positions. The alleles at 24 sites that separate these two haplotypes at various positions along Dup1 are presented sequentially between the red dotted boxes. **(A)** Haplotype-1 of Dup1 spans from Flank1 to inverted Dup2 (i.e., Flank1 + Dup1 + (inverted Dup2)). The purple lines represent overlapping reads of Nanopore containing Dup1 haplotype-1 alleles. The light blue dotted line represents the span of the same read from the end of haplotype-1 of Dup1 to the end of haplotype-2 of Dup2. **(B)** Haplotype-2 spans Dup1 from haplotype-2 of Dup2 to the Int region (i.e., (inverted Dup2) + Dup1 + Int). The blue lines represent the overlapping reads of Nanopore containing haplotype-2 alleles. The light blue dotted line represents the span of the same read from the start of haplotype-2 of Dup1 to the start of haplotype-2 of Dup2. Both haplotypes of Dup1 are connected to the single haplotype of Dup2 (i.e., haplotype-2 of Dup2) but at different ends.

Upon visual inspection of the long-read alignments in IGV at the Flank1–Dup1 junction region, three heterozygous sites (10766895, 10766948, and 10767078 at chromosome 20) were recognized at the Dup1 start region potentially separating the two haplotypes of Dup1 (Supplementary Figure S25). We extended these haplotypes toward the end of Dup1 by relying upon overlapping long-reads at haplotype-defining sites (**Methods**). To ensure the robustness of our read-backed phasing approach, we required that more than 10 sequencing reads support haplotype-specific alleles at each pair of adjacent sites (Supplementary Table S3). Dup1 haplotypes are anchored by (Flank1 end)/(Dup2 start) at the Dup1 start and (Int start)/(Dup2 end) at the Dup1 end (Figure 3). The Flank1 + Dup1 junction containing haplotype is referred to as Dup1 haplotype-1 (D1H1) and contains the allele T at position 10766895. The (inverted Dup2) + Dup1 junction containing haplotype is referred to as Dup1 haplotype-2 (D2H2) and contains the allele A at position 10766895. Each haplotype can be distinguished at 24 sites (Supplementary Table S3) along the Dup1 region. Each haplotype-defining site shares more than 10 long reads with haplotype-specific alleles at adjacent positions. Dup1 haplotype-1 (Figure 3A; Supplementary Figure S26A, B) spans Dup1 from Flank1 to Dup2 haplotype-2 (D2H2) end, and haplotype-2 (Figure 3B; Supplementary Figure S26A, B) spans Dup1 from Dup2 haplotype-2 (D2H2) start to Int start. Our inference of Dup1 haplotype-1 (i.e., Flank1 + Dup1 + (inverted Dup2)) and haplotype-2 (i.e (inverted Dup2) + Dup1 + Int) sequences rule out the *Fm_3 scenario, suggesting that either *Fm_1 and *Fm_2 scenario is possible.

**FIGURE 4:**
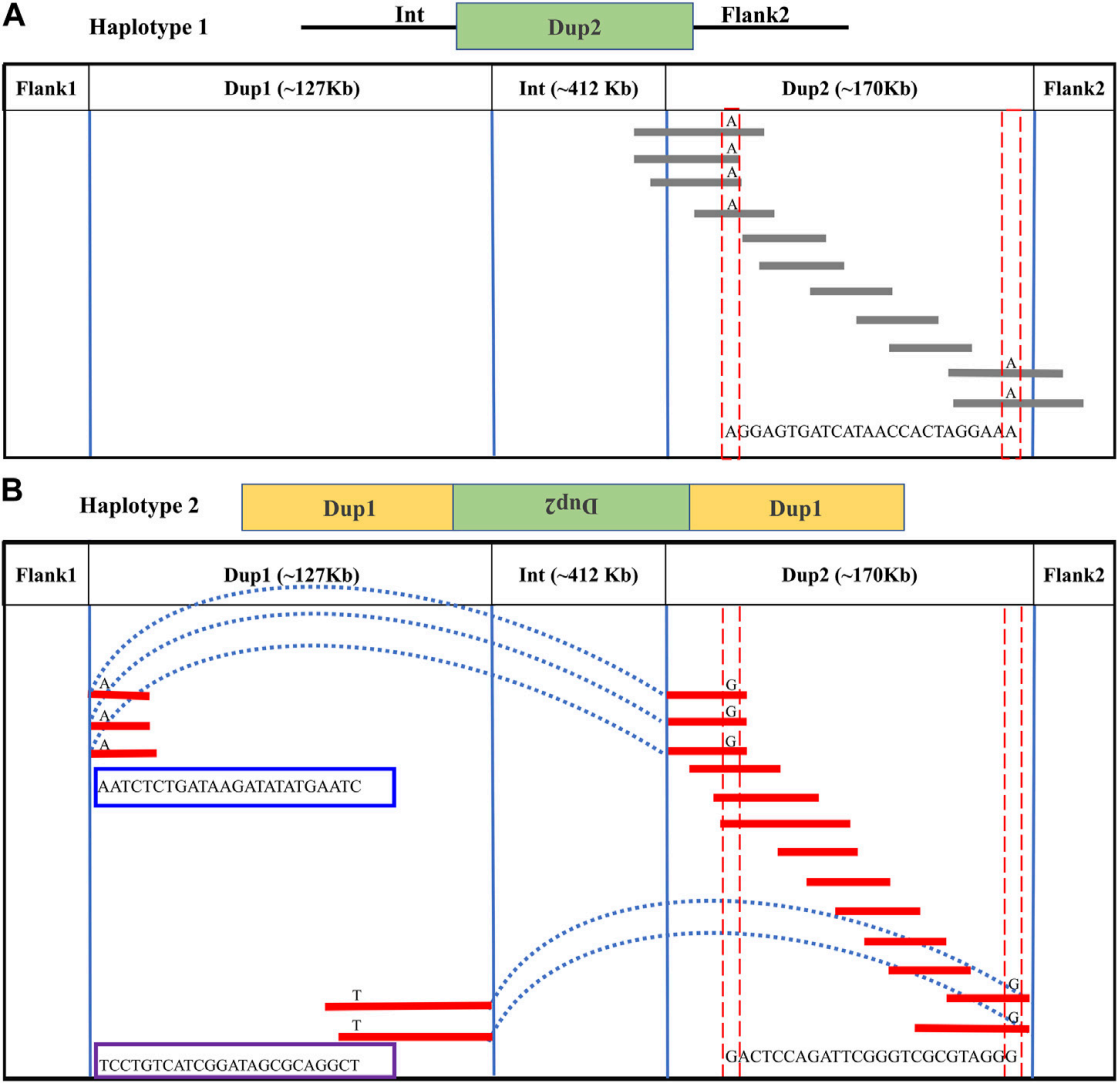
Long-read-based haplotypes resolve the sequence spanning the Dup2 region at *Fm* locus: The two haplotypes spanning the Dup2 region are distinguished by distinct alleles at 25 positions using long sequencing reads. Red dotted vertical boxes highlight the alleles that differ between haplotype-1 and haplotype-2 at the same positions. The alleles at 25 sites that separate these two haplotypes at various positions along Dup2 are presented sequentially between the red dotted boxes. **(A)** Haplotype-1 of Dup2 spans from Int to Flank2 (i.e., Int + Dup2 + Flank2). The gray lines represent overlapping reads of Nanopore containing Dup2 haplotype-1 alleles. **(B)** Haplotype-2 of Dup2 spans from the start of haplotype-2 of Dup1 to the end of haplotype-1 of the Dup1 region (i.e., Dup1 +(inverted Dup2)+ Dup1). The red lines represent the overlapping reads of Nanopore containing Dup2 haplotype-2 alleles. The light blue dotted line represents the span of the same reads from the start of haplotype-2 of Dup1 to the start of haplotype-2 of Dup2. Similarly, the same reads span the end of haplotype-1 of Dup1 to the end of haplotype-2 of Dup2.

**FIGURE 5:**
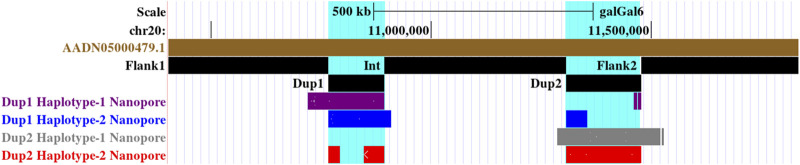
Screenshot of the UCSC genome browser of two haplotypes at Dup1 and Dup2 regions highlighted in cyan color identified using Nanopore long reads. Haplotype-1 of Dup1, shown in purple, spans from Flank1 to the end of inverted Dup2 (i.e., Flank1 + Dup1 + (inverted Dup2)). Haplotype-2 of Dup1, shown in blue, spans from the start of inverted Dup2 to the Int region (i.e, (inverted Dup2) + Dup1 + Int). Haplotype-1 of Dup2, shown in gray, spans from Int to Flank2 (i.e., Int + Dup2 + Flank2). Haplotype-2 of Dup2, shown in red, spans from the start of haplotype-2 of Dup1 to the end of haplotype-1 of the Dup1 region (i.e., Dup1 +(inverted Dup2)+ Dup1).

In the Dup2 region, one haplotype was anchored to the Dup2+ Flank2 junction and another haplotype to the Dup2 + (inverted Dup1) junction. The Dup2 + Flank2 junction containing the haplotype is Dup2 haplotype-1 (D2H1) and contains the allele A at position 11476819. The Dup2 + (inverted Dup1) junction containing the haplotype is Dup2 haplotype-2 (D2H2) and contains the allele G at position 11476819 (Supplementary Figure S27). To verify the correct arrangement, we used the same read phasing approach on the Dup2 region, and we could distinguish the two haplotypes at the Dup2 region based on 25 sites (Supplementary Table S3). Haplotype-1 of the Dup2 region (Figure 4A; Supplementary Figure S28, 29) spans Dup2 from the end of Int to the start of Flank2, and haplotype-2 (Figure 4B; Supplementary Figure S28, 29) spans Dup2 from Dup1 haplotype-2 (D1H2) start to Dup1 haplotype-1 (D1H1) end. Our inference of haplotype-1 of Dup2 (i.e., Int+ Dup2 + Flank2) and haplotype-2 (i.e., start of Dup1 + (inverted Dup2) + Dup1 end) sequences rule out the *Fm_1 and *Fm_3 scenarios, suggesting that the *Fm_2 scenario is correct (Figure 5).

### All black-bone chicken breeds share the same rearrangement junctions at the *Fm* locus

**FIGURE 6:**
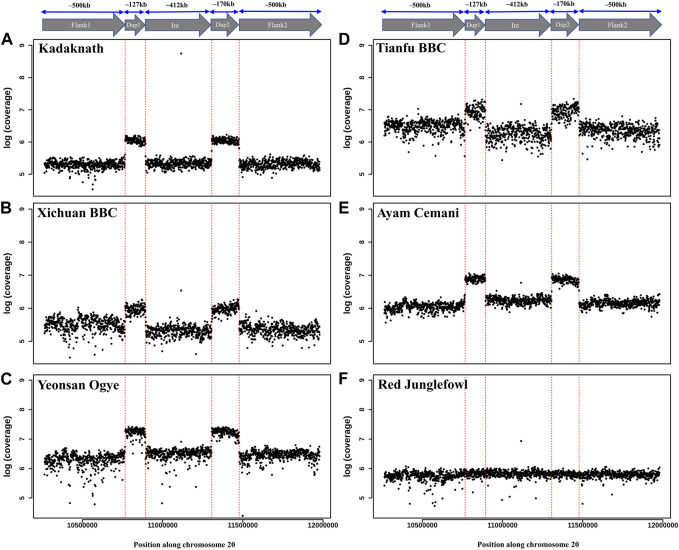
Comparison of *Fm* locus read coverage in black-bone vs. non-black-bone chicken breeds: read coverage along chromosome 20 at the *Fm* locus is shown in 1 Kb sliding windows. Two duplicated genomic loci, Dup1 and Dup2, are denoted by vertical dotted red lines and have a higher coverage in BBC breeds: **(A)** Kadaknath, **(B)** Xichuan, **(C)** Yeonsan Ogye, **(D)** Tianfu, **(E)** Ayam Cemani than non-BBC, and **(F)** red jungle fowl (RJF).

**FIGURE 7:**
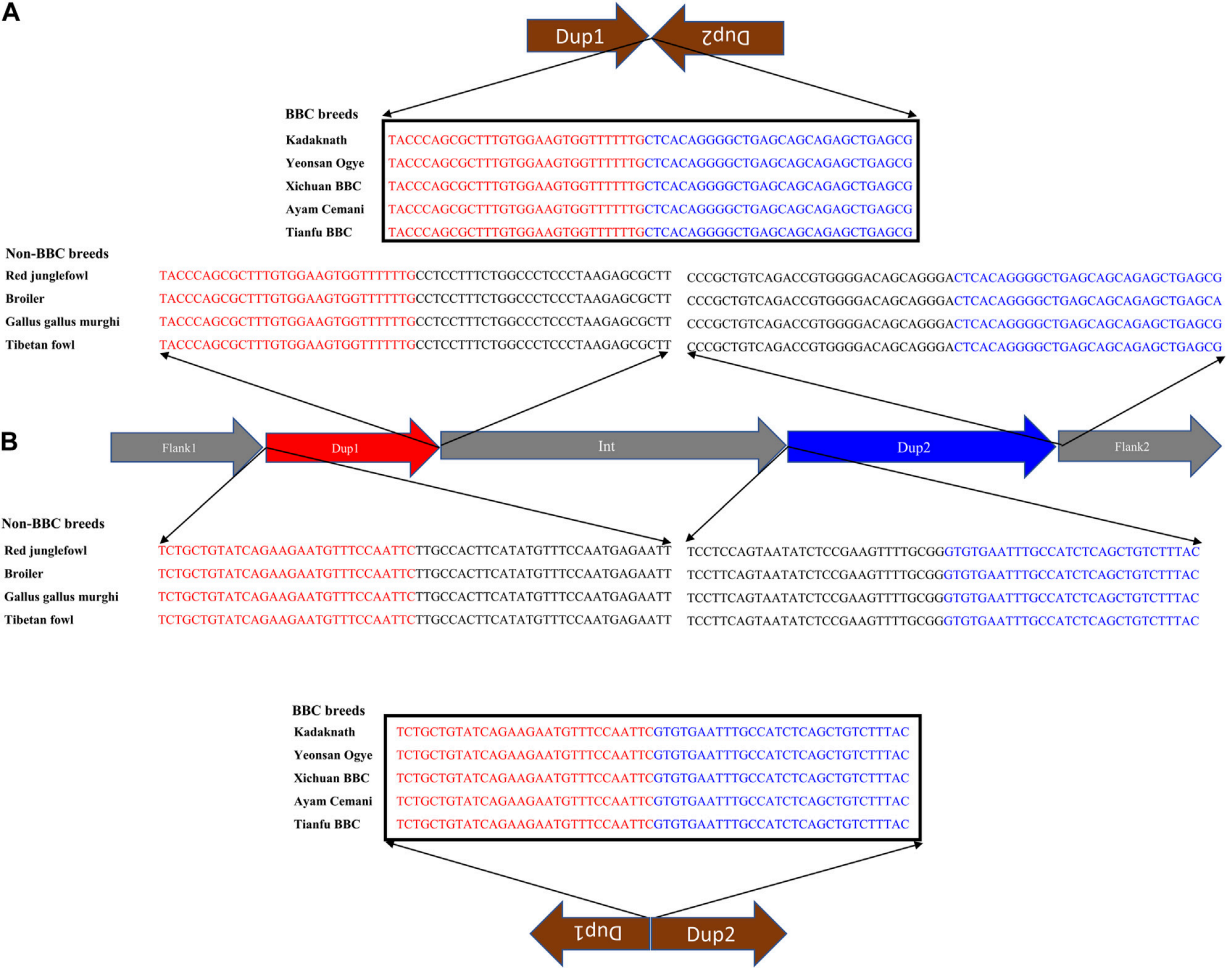
Nucleotide sequence of rearranged junctions at the *Fm* locus in black-bone vs. non-black-bone chicken: **(A)** Dup1 + (inverted Dup2) **(B)** (inverted Dup1) + Dup2. Brown arrows represent the direction of rearranged junction sequence, which is present as a continuous sequence (verified by their presence in a single read) in BBC breeds. In contrast, non-BBC breeds did not contain these junctions. Sequences highlighted in red and blue represent the Dup1 and Dup2 sequences, respectively. Sequences in black color represent the subsequent nucleotides.

Conclusive inference regarding which scenario is present in the BBC has been extremely challenging due to the large (∼1 Mb) size and complexity of the rearrangement. However, in the case of Kadaknath, it has not even been established whether Dup1 + (inverted Dup2) and (inverted Dup1) + Dup2 junctions identified in other BBCs are present. We have compared the normalized short-read coverage at these junctions to evaluate whether all BBC breeds share the same rearrangement junctions at the *Fm* locus. The normalized short-read coverage in BBCs (Figures 6A–E) abruptly increases at Dup1 and Dup2 regions, while no such increase occurs in non-BBCs (Figure 6F). The drastic change in coverage at Dup1 and Dup2 boundaries occurs at the same base in all BBCs (Supplementary Figure S30–34). In the case of non-BBCs, no change in coverage occurs at this position (Supplementary Figure S35). We also identified the black-bone-specific *Fm* locus junctions Dup1 + (inverted Dup2) and (inverted Dup1) + Dup2 using published PacBio data (Figures 7A,B and Methods). Short-read coverage spanning these black-bone-specific *Fm* locus junctions is present in the BBC and missing in the non-BBC (Supplementary Figure S36–38). We can infer that BBC defining *Fm* locus originated through a single event based on coverage and rearranged junction sequence.

Crossbreeding with native and commercial breeds affects the genome-wide patterns of genetic variation in black-bone breeds (Supplementary Figure S17-21A–C; Supplementary Figure S39, 40). Hence, we focused on the *Fm* locus to investigate the history of the BBC breeds. The F_ST_ between the BBC and non-BBC along chromosome 20 is elevated at Dup1 and Dup2 regions (see Supplementary Figure S41). However, other population genetic parameters, such as genetic diversity (*π* and θ) and divergence (D_xy_), lack any prominent signatures (Supplementary Figure S42–44). As evident from the elevated F_ST_, the major axis of genetic variation (assessed using local PCA) in Dup1 and Dup2 regions separates the BBC from non-BBCs (Supplementary Figure S45, 46A–E). A phylogenetic tree of the SNPs from Dup1 and Dup2 regions also largely separates the BBC breeds from non-BBC breeds (Supplementary Figure S47A, B). Our evaluation of the genetic differentiation (F_ST_) landscape between BBC breeds found reduced F_ST_ at Dup1 and Dup2 regions compared to the genomic background (Supplementary Figure S48). However, comparing individual BBC breeds with non-black chicken breeds showed the opposite pattern with elevated F_ST_ at Dup1 and Dup2 regions (Supplementary Figure S49, 50). Hence, the patterns of genetic differentiation (F_ST_), local PCA, and phylogenetic tree also support a common origin of the *Fm* locus in all BBC breeds.

### Isolation by distance pattern suggests dispersal between India and China

Our analysis discovered that the rearrangement junctions in all BBCs are identical to the one in Kadaknath and strongly support a common origin for the *Fm* locus. An independent origin for the *Fm* locus would mean separate rearrangement events have created identical junction sequences. Given the lack of repeat sequences at the junctions, such independent origins seem highly unlikely and are not consistent with the genetic relatedness of BBC and non-BBC breeds at the *Fm* locus. Hence, the current distribution of BBC breeds across Asia needs an explanation. We hypothesized (see a schematic of the proposed scenario in Supplementary Figure S51) that the dispersal of BBC occurred after a common origin for the *Fm* locus, followed by recent crossbreeding with native and commercial breeds.

To test our hypothesis and delineate the dispersal route of different black-bone breeds, we first evaluated if a pattern of isolation by distance (IBD) is prevalent. The IBD plot shows a consistent increase in genetic distance with an increase in the geographic distance (Supplementary Figure S52, mantel’s r = 0.64, *p*-value = 0.0002). The IBD pattern persisted even after we repeated the analysis using ANGSD/variant-call-based estimates of nucleotide differentiation (F_ST_) after excluding various populations to avoid errors due to auto-correlation and biased estimates from isolated populations (Supplementary Figure S53–59). Among the black-bone breeds, Tibetan black-bone (TBTC) and Sichuan black-bone (LCTMJ) chickens are genetically closest to Kadaknath (mean F_ST_∼0.17). The other black-bone breeds that are geographically more distant from Jhabua (LCEM, mean F_ST_ = 0.21; XBBC, mean F_ST_ = 0.22; LCMY, mean F_ST_ = 0.3; and YOSK, mean F_ST_ = 0.36) occur at increasing genetic distances from Kadaknath (Supplementary Table S5). The pattern of IBD spanning India and China suggests potentially human-mediated dispersal. We lack conclusive evidence to identify the direction of dispersal. However, the analysis of our dataset, even after excluding alleles found in dbSNP (Sherry et al., 2001) (Supplementary Figure S60, 61), found that Kadaknath has almost twice the number of private alleles than Chinese and Korean BBC breeds and suggests an India-to-China dispersal. More extensive fine-scale sampling may provide a definitive answer regarding the direction and timing of the dispersal.

### Genome-wide signatures of selection in Kadaknath chicken

**FIGURE 8:**
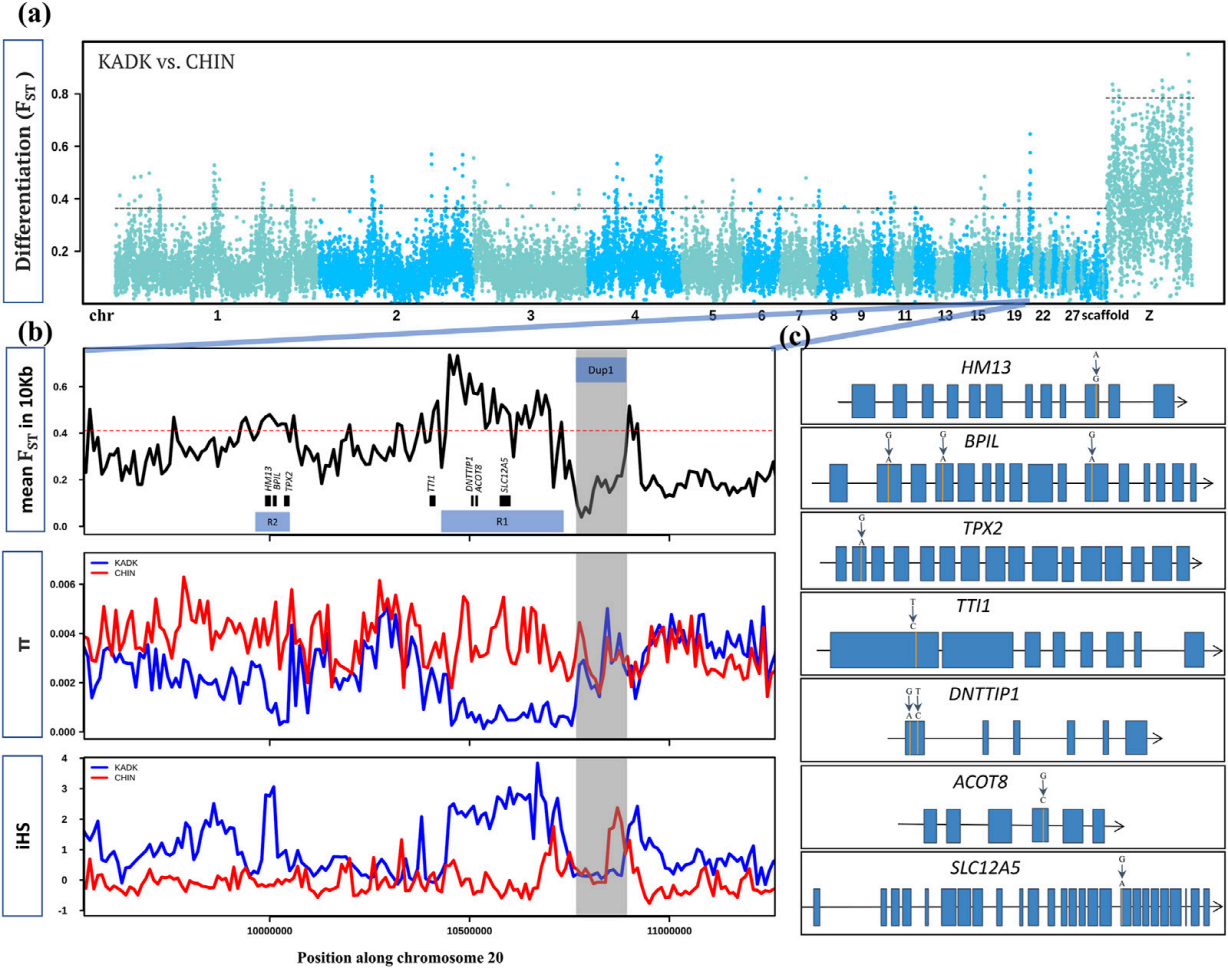
Within and between population comparison of KADK with CHIN: **(A)** genome-wide landscape of pairwise genetic differentiation (F_ST_) between KADK and CHIN in 50 Kb non-overlapping windows. The dark slate gray and deep sky blue colors represent the alternative chromosomes. The dotted horizontal black line marks the 99th percentile outlier of estimated F_ST_ for autosome and the Z chromosome, respectively. **(B)** Highest F_ST_ region with a prominent pattern occurs on chromosome 20. The panels from top to bottom show estimates of F_ST_ in 10 Kb non-overlapping windows within population pairwise nucleotide diversity (π) and integrated haplotype score (iHS) with the major allele as ancestral and the minor allele as derived. The genes are represented as black boxes with names. The horizontal red dotted line marks the 99th percentile 10 Kb F_ST_ outlier. The transparent gray color highlights the Dup1 region in all panes in the B panel, while R1 and R2 represent the selective sweep regions. **(C)** Non-synonymous changes (shown above the exon) within genes in the regions R1 and R2 are denoted by vertical orange lines. Blue boxes are the exons connected by black lines representing introns.

We screened the genome for selection signatures to identify all Kadaknath-specific regions by comparing Kadaknath with Chinese BBC. We also compared Kadaknath and a Chinese BBC population (XBBC, n = 4) with the Korean Yeonsan Ogye to identify population-specific signatures. Despite the sizeable geographic separation, the genome-wide mean F_ST_ between KADK and CHIN is 0.16, making it amenable to identifying selection signatures ( Figure 8A; Supplementary Table S6). A pairwise comparison between KADK and CHIN revealed 137 genic regions in the top 1% F_ST_ windows ( Supplementary Table S7; Supplementary Figure S62–94). We further shortlisted candidates using the additional criteria that genetic diversity (*π* and θ) and integrated haplotype score (iHS) should be strikingly different between KADK and CHIN. Two prominent signatures from these shortlisted regions indicative of selective sweeps in the KADK breed are evident on the 20th chromosome in the vicinity of the Dup1 region. The first region (R1) of ∼300 Kb is ∼160 Kb before Dup1, and the second region (R2) of ∼70 Kb is ∼0.7 Mb before Dup1. In both regions, the genetic diversity (*π* and θ) and Tajima’s D (τ) are strongly reduced in KADK compared to CHIN (Figure 8B; Supplementary Figure S81). Compared to CHIN, the elevated iHS in KADK and the positive value of extended haplotype homozygosity (XP-EHH) support a selective sweep in KADK. This region also occurs in the top 10% genome-wide genetic divergence (D_xy_) windows ( Supplementary Figure S95.

In the first region (R1), deoxynucleotidyltransferase terminal interacting protein 1 (*DNTTIP1*), acyl-CoA thioesterase 8 (*ACOT8*), and solute carrier family 12 member 5 (*SLC12A5*) genes contain non-synonymous changes. The TELO2-interacting protein 1 homolog (*TTI1*), which occurs near the first sweep region (R1), also has a non-synonymous change (Figure 8C; Supplementary Table S8, 9). The second sweep region (R2) contains three genes with non-synonymous changes: histocompatibility minor 13 (*HM13*), bactericidal/permeability-increasing protein-like (*BPIL*), and targeting protein for Xklp2 (*TPX2*). The most striking differences were in the *BPIL* gene, which has three fixed sites with non-synonymous changes. Of these changes, two alleles are unique to the KADK population (Supplementary Figure S96). The first non-synonymous change (C**G**G->C**A**G, R->Q) occurs at position 69 in exon 2. The second non-synonymous change (**G**AG->**A**AG, E−>K) is within the first BPI superfamily protein domain at position 159 in exon 4. The third and last non-synonymous change (C**G**C->C**A**C, R->H) is in the second domain at position 405 in exon 11. The impact of non-synonymous changes (R69Q, E159K, and R405H) in *BPIL* specific to Kadaknath is annotated as a moderate effect in the snpEff database. The PolyPhen-2 (Polymorphism Phenotyping v2) tool found that the non-synonymous changes R69Q and E159K are possibly damaging, while R405H is probably damaging (Supplementary Figure S97–99). We also found that *HM13*, *SLC12A5*, and *DNTTIP1* have novel Kadaknath-specific changes. The *HM13* gene is highly expressed in the muscle tissue, and we could assess the Kadaknath-specific change in all the native Indian breeds. Only the Kadaknath transcriptome contained the change identified in the genome sequencing data.

The Korean Yeonsan Ogye breed is closer to the Chinese XBBC (mean F_ST_ = 0.26) than to Kadaknath (mean F_ST_ = 0.3). However, the genome-wide mean F_ST_ is still low enough to distinguish selection signatures. A comparison of YOSK with KADK confirmed that R1 and R2 regions are specific to Kadaknath (Supplementary Figure S100; Supplementary Table S6). Surprisingly, our comparisons with YOSK identified a third region (R3) of ∼570 kb, which is ∼2.16 Mb before Dup1 with a sweep specific to YOSK (Supplementary Figure S101; Supplementary Table S9). Local PCA of the *Fm* locus, R1, and R2 regions demonstrates that Flank1, R1, and R2 regions have changed only in Kadaknath and not in other BBC (Supplementary Figure S102A–E; Supplementary Figure S103, 104). A careful examination of entire chromosome 20 failed to identify any other sweep regions (Supplementary Figure S105). Similar to the sweep in KADK, we found two genomic regions (on Chr 4 and Chr 9) with signatures of a sweep in the CHIN population (Supplementary Table S9). The sweep region on Chr 4 contains the *PCDH7* gene, and the region on Chr 9 contains the *COL4A3* and *MFF* genes (Supplementary Figure S65, 70). However, none of these genes have any non-synonymous fixed differences.

## Discussion

### All BBC breeds share a common origin of the *Fm* locus

As part of this study, we have generated the first whole-genome dataset of the Kadaknath breed spanning all three morphs. Hence, we can evaluate the relationship between all BBC breeds by comparing our data with public datasets. Our comparison of the *Fm* locus junction region in genome sequencing data from BBC breeds conclusively establishes a common origin for the complex chromosomal rearrangement (Dharmayanthi et al., 2017). The following four lines of evidence support this conclusion: (1) the short-read coverage along the *Fm* locus, (2) the high-sequence identity of the rearranged junctions across the BBC breeds, (3) the local and phylogenetic relationship between chicken breeds at the *Fm* locus, and (4) the patterns of pairwise genetic differentiation between BBC and non-BBC breeds. Independent structural variants can produce the same phenotypes, as seen in the case of blue eggshell (Wang et al., 2013). However, in the case of BBC breeds, the hyperpigmentation phenotype results from a single shared complex chromosomal rearrangement.

### Spread of BBC across Asia

The earliest records of BBC have been found in the writings of Marco Polo, the Venetian explorer–writer who traveled through Asia (Dorshorst et al., 2011). The Compendium of Materia Medica or Bencao Gangmu, compiled and edited by Li Shizhen and published in the late 16th century, attributes various medicinal properties to BBCs (Wang et al., 2021). In Korea, the BBC is thought to have had a royal connection, and “Dongui Bogam,” a traditional Korean medical encyclopedia compiled and edited by Heo Jun in 1,613, records the medicinal use of BBC (Sohn et al., 2018). While an earlier study notes that “In 1635 AD, the finding of chickens with black meat (typical of fibromelanosis, FM mutation) in Mozambique suggested direct introductions from India” (Tixier-Boichard et al., 2011), we could not find literature on how Kadaknath ended up in Jhabua, India. However, Indian chickens are thought to have entered Africa through Egypt (contributing to breeds such as Fayoumi) before dispersing into Europe (Eltanany and Hemeda, 2016).

Jhabua is close to the ancient port cities of Bharuch (also known as Bharukaccha, Barygaza, and Broach) (260 Km) and Lothal (300 Km) on the west coast of India. Given the proximity to port cities and the prevalence of BBC only in Western India (and the spread of BBC to Africa), we suspected that Kadaknath may have traveled through a marine route. The ancient sea trade between India, Korea, and other parts of Asia are well documented (Acri, 2018). Moreover, the movement of domesticated breeds during the period of colonialism was also facilitated by the common rule of large parts of Asia by various European powers (Sudrajad et al., 2020). We find evidence of crossbreeding between Kadaknath and Ankleshwar chicken breeds. The Ankleshwar breed of chicken is named after the city of Ankleshwar, located near modern-day Bharuch. Similar to Kadaknath, the Ankleshwar breed is reared by tribal communities of southern Gujarat. Evidence of gene flow between these native Indian breeds suggests that the rearing of Kadaknath and Ankleshwar breeds overlapped. None of the other native Indian breeds share ancestry with Kadaknath and support the movement of Kadaknath through the port city of Bharuch via a marine route.

Historical records are patchy, prone to error/obfuscation, and fail to provide conclusive information about the spread of BBC across Asia (Peters et al., 2022). Hence, we evaluated the relationship between BBC breeds and patterns of isolation by distance to infer the ancient dispersal route. Although each BBC breed has considerable genetic distinctiveness, population genetic analyses support the common heritage of all BBC breeds and reveal a trend of isolation by distance. While IBD patterns are well established in Chinese native chicken breeds (Nie et al., 2019), our dataset spans BBC breeds from India, China, Indonesia, and Korea. The sample size of some BBC breeds in our analysis is limited, and the sampling does not cover the entire geographic distribution of some breeds. However, our analysis achieves genome-wide pan-BBC breed sampling by including all the major breeds from Asia.

The BBC chicken from Tibet is genetically closest to the Kadaknath breed and suggests that the old Tibet–Nepal salt trade route or the maritime silk route may have facilitated the spread of BBC. Interestingly, the Tibetan BBC is genetically more similar to Kadaknath than some Chinese BBC breeds. Unfortunately, we lack BBC samples from Nepal. However, we believe BBC poultry in Nepal is Kadaknath chicken, recently imported from India. Hence, the ancestral stock of the BBC spread across Asia may not be currently available. Changes in trade routes and the introduction of commercial poultry breeds limit our ability to trace the historical prevalence of BBC breeds.

### Did black-bone chicken originate in India?

The lack of data from India and the considerable interest in BBC breeds in Europe, China, and Korea have meant that studies have focused mainly on non-Indian BBCs. Hence, Southern China and Tibet are considered the source of all BBC breeds (Zhu et al., 2014; Zhang et al., 2018; Xue et al., 2023). However, despite a traditionally restricted geographic distribution, the Kadaknath breed has nucleotide diversity comparable to Chinese BBC breeds and much higher than the Korean BBC. The number of private alleles identified in Kadaknath is also greater than that in Chinese and Korean BBC breeds. Moreover, the mean genome-wide F_ST_ of 0.11 between Jhabua and Bhopal is comparable to the differentiation between some Chinese BBC breeds. The presence of three distinct morphs within Kadaknath chicken suggests phenotypic diversity derived either from commercial breeds, native Indian breeds such as Ankleshwar, or existing variations within Kadaknath. The extent of phenotypic diversity within BBC breeds from China and Korea is not documented to allow a fair comparison. The high genetic diversity in Kadaknath supports the potential origin of all BBC in Jhabua, India. The export of black chicken from India to Africa in ∼1600 AD also supports that BBC was present in ancient India (Tixier-Boichard et al., 2011).

While we cannot conclude whether BBC had an Indian, Chinese, or more Southeast Asian origin, our data suggest that any of these sources are plausible. More widespread geographic sampling and analysis of allele-sharing patterns may provide a definitive answer regarding the origin of BBC. For instance, the human-aided dispersal of the ginkgo tree out of China could be traced back to samples from eastern China using extensive sampling (Zhao et al., 2019). Future studies using such large-scale sampling could provide a more definitive answer regarding the origin and dispersal of BBC. Unlike the initial domestication of the chicken, which may have occurred independently (Tixier-Boichard et al., 2011; Mariadassou et al., 2021) in several locations, the BBC has a single origin linked to the rearrangement at the *Fm* locus. Irrespective of the source of all BBCs, we identify several Kadaknath-specific genetic changes. Hence, the genetic distinctiveness of the Kadaknath breed has long diverged from other BBC breeds and is a result of its unique heritage sustained by the Bhil and Bhilala tribal communities of Madhya Pradesh. The beliefs and practices of tribal communities may have contributed to the domestication and conservation of Kadaknath (Dar et al., 2019).

### Conclusive resolution of the complex chromosomal rearrangement

Earlier studies of the *Fm* locus have proposed three possible scenarios for the complex chromosomal rearrangement. Although the *Fm_2 scenario was favored based on crosses between BBC and non-BBC breeds, the correct scenario was not established by previous studies as the genome assembly of this region is challenging. We use a haplotype phasing approach relying on published long-read datasets to conclusively resolve the correct arrangement to be the *Fm_2 scenario at the *Fm* locus. In the *Fm_2 scenario, the distal region (Dup1+Int + Dup2+Flank2) resembles the *N arrangement found in non-BBC breeds. However, the proximal region (Flank1+Dup1+(inverted Dup2)) has a very different arrangement than the non-BBC *N arrangement. In contrast to the *Fm_2 scenario, the proximal region would be similar to the *N arrangement in *Fm_3 scenario. In the *Fm_1 scenario, only the first occurrence of Dup1 and the last occurrence of Dup2 are similar to the *N arrangement. Hence, in the *Fm_2 and *Fm_3 scenarios, recombination with *N arrangement may be easier (i.e., recombination is suppressed only in the inverted ∼127 Kb (Dup1) or 170 Kb (Dup2) region) compared to the * Fm_1 scenario (i.e., recombination is suppressed in ∼709 Kb (inverted (Dup2+Int + Dup1)) region). The ease of recombination with *N arrangement could explain the prevalence of the *Fm_2 scenario, which requires two rearrangement events. Ongoing improvements to genome assembly methods such as haplotype-aware *de novo* assembly (Chin et al., 2016; Korlach et al., 2017; Garg et al., 2018; Sohn and Nam, 2018) and/or Strand-seq (Falconer et al., 2012; Sanders et al., 2017; Ghareghani et al., 2018) should allow easier resolution of such complex chromosomal rearrangements.

### Selective sweep near the *Fm* locus may be a consequence of linkage

Our analyses identified two genomic regions (R1 and R2) in the vicinity of the *Fm* locus with prominent signatures of a selective sweep in the KADK population. The most promising candidate gene in this region that may have been the focus of selection is the *BPIL* gene which has accumulated three non-synonymous changes predicted to be functionally important. The *BPIL* gene is part of the innate immune defense system, which binds and neutralizes lipopolysaccharides (LPS) from the outer membrane of Gram-negative bacteria (Holweg et al., 2011; Sun and Sun, 2016; Monson et al., 2019). *BPIL* is associated with upregulated expression after exposure to heat stress and LPS treatment in a comparative transcriptomic study between Fayoumi and broiler chicken breeds (Monson et al., 2019). The Fayoumi chicken breed is relatively resistant to Newcastle disease virus (NDV) compared to Leghorns (Deist et al., 2017) and potentially shares ancestry with Indian chicken breeds (Eltanany and Hemeda, 2016). Earlier studies have shown that the Kadaknath has a high level of disease tolerance against Newcastle disease, also known as Ranikhet disease, compared to other chicken breeds (Kokate et al., 2017; Gumasta et al., 2021; Malarmathi et al., 2023). Along with resistance against Newcastle disease, it has been reported that the Kadaknath breed is less sensitive to coccidial infection (Thakur et al., 2015). The non-synonymous variants in the *BPIL* gene might enhance the disease resistance in Kadaknath against various bacterial and viral infections and be associated with a better immune response. Further work to functionally evaluate the effect of these changes will provide a clear answer.

Five *BPI*-like genes occur near *BPI* (Chiang et al., 2011), which help arrest bacterial growth, are prominent in neutrophil phagocytosis, and work as a bactericidal protein (Elsbach and Weiss, 1998; Weiss, 2003). In chickens, defense against Gram-negative bacteria may be especially important as they lack the complement *C9* gene required to effectively eliminate pathogens through the membrane attack complex (MAC) formation (Sharma et al., 2022a). Notably, another BBC breed from Korea, YOSK, has signatures of selection at the *TLR4* gene involved in detecting Gram-negative bacteria (Cho et al., 2022). Hence, various immune genes may have been selected in different chicken breeds to protect against Gram-negative bacteria.

Several other genes with non-synonymous changes occur in R1 and R2 genomic regions. Although we cannot identify the functional consequences of these changes, the strong signature of selection and KADK-specific protein-coding alterations suggest phenotype-altering ability. Predictions from snpEff and PolyPhen-2 also suggest a major effect of these protein-coding changes located within the domain regions. The genomic region of high differentiation is close to the *Fm* locus, while weak genome-wide differentiation (except for chr 4 and chr9 regions) between Kadaknath and Chinese BBCs is strongly suggestive of hitchhiking. Moreover, the sweep signature is in the Chinese BBC in chr 4 and chr 9 cases. Hence, the proximity of R1 and R2 regions to the *Fm* locus indicates that selective sweep may be due to close physical linkage with the *Fm* locus. Such co-selection of traits due to linkage and pleiotropy has occurred during animal domestication, including in chicken (Rubin et al., 2010; Feder et al., 2014; Webster et al., 2015). Hitchhiking of alleles can increase the frequency of both beneficial and mildly deleterious alleles (Kirkpatrick and Barton, 2006; Bierne, 2010; Huff et al., 2012; Flaxman et al., 2013; Franssen et al., 2015; Martin et al., 2020; Hale et al., 2021; Weist et al., 2022). Whether the changes in KADK are beneficial or deleterious need to be investigated in future studies.

The genomic co-occurrence of economically important traits in domesticated plants and animals is also known (Zhang et al., 2010; Kijas et al., 2012; Beissinger et al., 2014; Fang et al., 2017; Kong et al., 2018; Huang et al., 2020a; Wang et al., 2020b; Jayakodi et al., 2020; Guan et al., 2021; Li et al., 2022a). The alleles causing phenotypic changes can occur in closely linked genes and will undergo selection for any of the phenotypes being favored by the breeding process. Notably, R1 and R2 selective sweeps are found only in KADK, R3 in YOSK, and none in Chinese BBC breeds. Hence, the genetic variants and the selective sweep may represent recent events after the BBC breeds separated from each other. Comparing different BBC breeds provides snapshots of the selection process and the build-up of genetically linked co-selected allelic changes and may help understand the hitchhiking process during domestication.

Genome-wide data generated as part of this study and comparative analysis with other BBC breeds establish the genetic uniqueness of Kadaknath that extends beyond the *Fm* locus. We also identify specific genes with selection signatures that are likely responsible for the Kadaknath-specific phenotypes. The co-selection of genes that are in linkage, as shown in Kadaknath, is widespread in domestic species (Rubin et al., 2010; Zhang et al., 2010; Webster et al., 2015; Fang et al., 2017; Kong et al., 2018; Li et al., 2022a). Such clustering of linked alleles under selection is favored in low recombination regions and near chromosomal rearrangements. Our work exemplifies the interaction of artificial selection and chromosomal rearrangement-linked traits in domesticated species.

## Data Availability

The datasets presented in this study can be found in online repositories. The names of the repository/repositories and accession number(s) can be found at: https://www.ebi.ac.uk/ena, PRJEB51457. Scripts and data are available at: https://github.com/ceglabsagarshinde/Kadaknath_Project and https://doi.org/10.17632/8f9dn6h76h.1.
