# Supplementary material for: Decoding the fibromelanosis locus complex chromosomal rearrangement of black-bone chicken: genetic differentiation, selective sweeps and protein-coding changes in Kadaknath chicken
Source: Front Genet. 2023 Jun 22;14:1180658. doi: 10.3389/fgene.2023.1180658 (PMC10325862; doi:10.3389/fgene.2023.1180658)
Supplement: Supplementary file 1 [file DataSheet2.PDF]

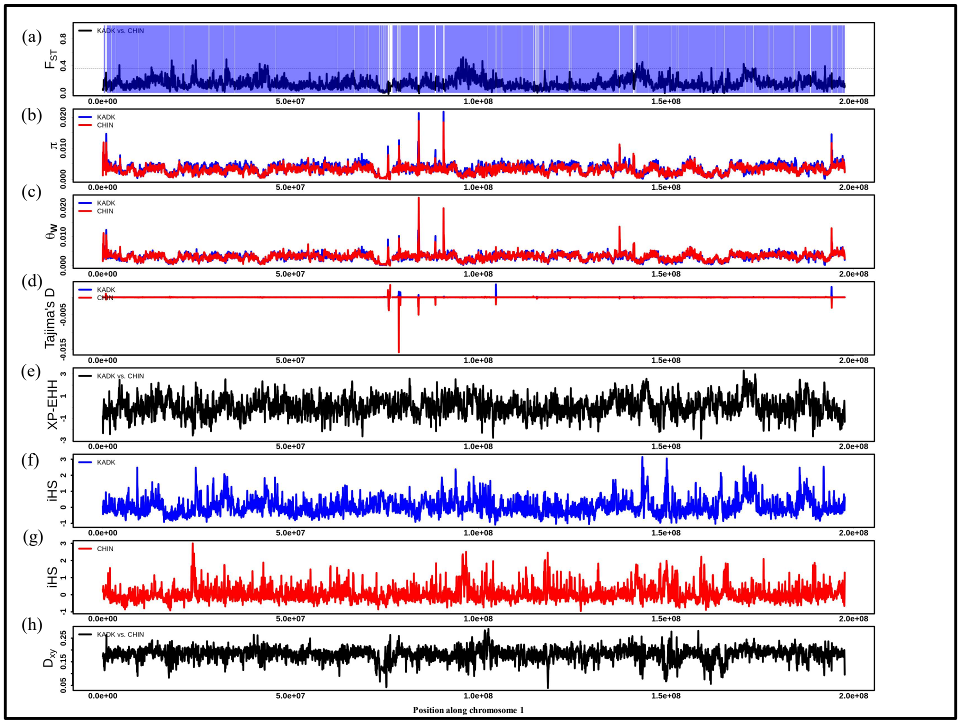

Supplementary Figure 62. (a) Pairwise F_{ST} comparison between KADK and CHIN population along chromosome 1 using 50Kb windows. A horizontal black dotted line represents the 99 percentile F_{ST} threshold. >80 percent callable region shown in transparent blue color while white color region represents <80 percent callable region. **b,c,d.** represents the π, Watterson theta, and Tajima`s D, respectively, where the solid blue line represents the KADK, and the solid red line represents the CHIN population. **(e)** Pairwise XP-EHH comparison between KADK and CHIN using 50Kb window. **f, g.** iHS results visualized in 50Kb window along the chromosome for KADK shown in solid blue color and CHIN shown in solid red color, respectively. **(h)** Dxy between KADK and CHIN.

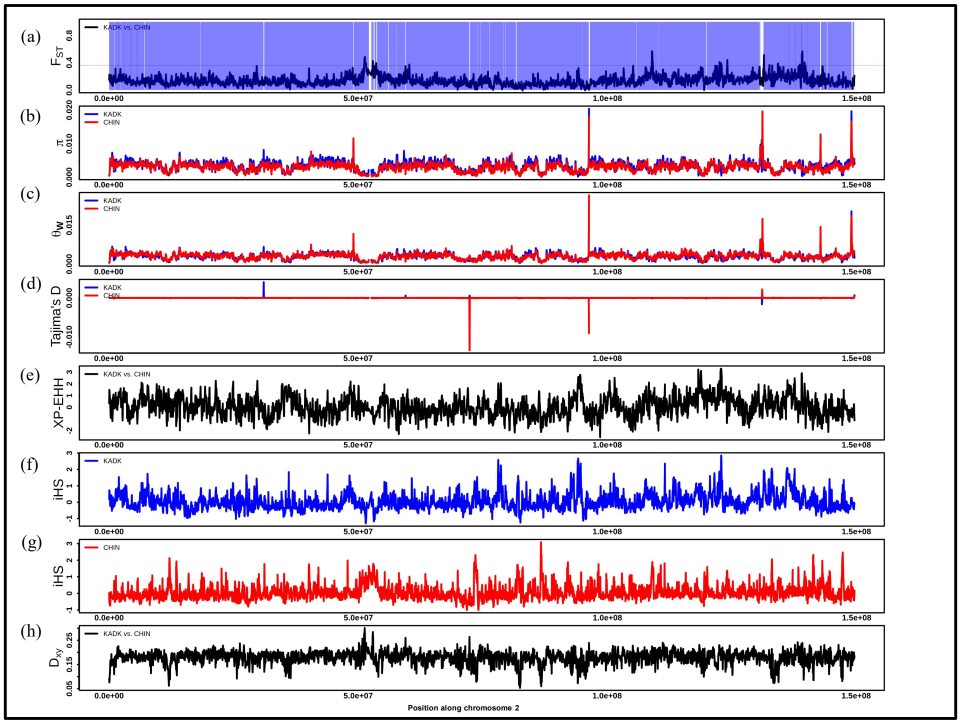

Supplementary Figure 63. (a) Pairwise F_{ST} comparison between KADK and CHIN population along chromosome 2 using 50Kb windows. A horizontal black dotted line represents the 99 percentile F_{ST} threshold. >80 percent callable region shown in transparent blue color while white color region represents <80 percent callable region. **b,c,d.** represents the π, Watterson theta, and Tajima`s D, respectively, where the solid blue line represents the KADK, and the solid red line represents the CHIN population. **(e)** Pairwise XP-EHH comparison between KADK and CHIN using 50Kb window. **f, g.** iHS results visualized in 50Kb window along the chromosome for KADK shown in solid blue color and CHIN shown in solid red color, respectively. **(h)** Dxy between KADK and CHIN.

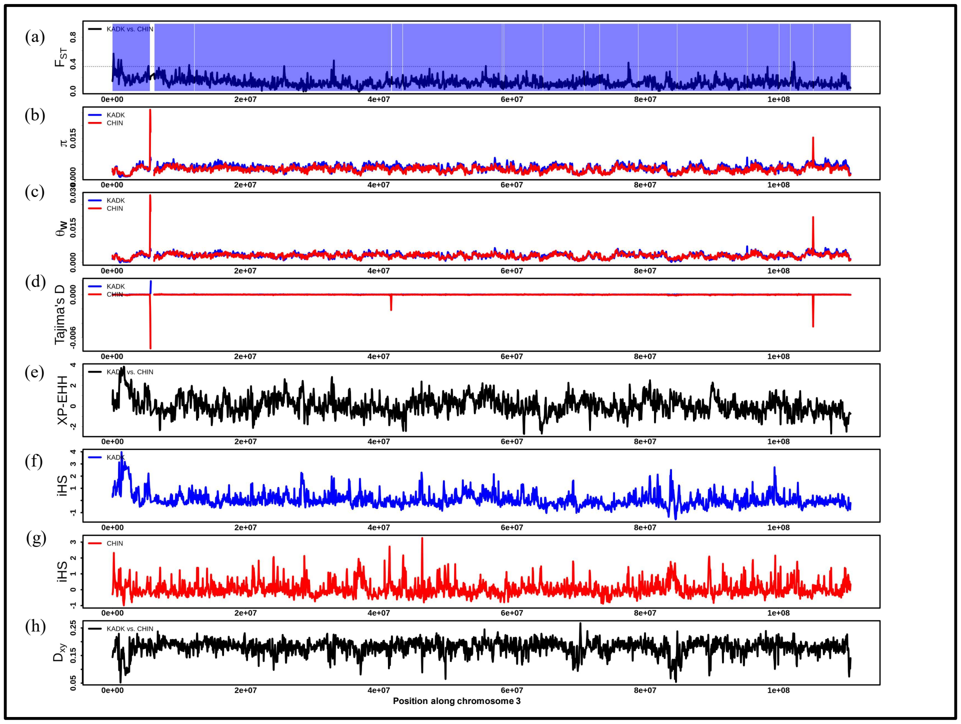

Supplementary Figure 64. (a) Pairwise F_{ST} comparison between KADK and CHIN population along chromosome 3 using 50Kb windows. A horizontal black dotted line represents the 99 percentile F_{ST} threshold. >80 percent callable region shown in transparent blue color while white color region represents <80 percent callable region. **b,c,d.** represents the π, Watterson theta, and Tajima`s D, respectively, where the solid blue line represents the KADK, and the solid red line represents the CHIN population. **(e)** Pairwise XP-EHH comparison between KADK and CHIN using 50Kb window. **f, g.** iHS results visualized in 50Kb window along the chromosome for KADK shown in solid blue color and CHIN shown in solid red color, respectively. **(h)** Dxy between KADK and CHIN.

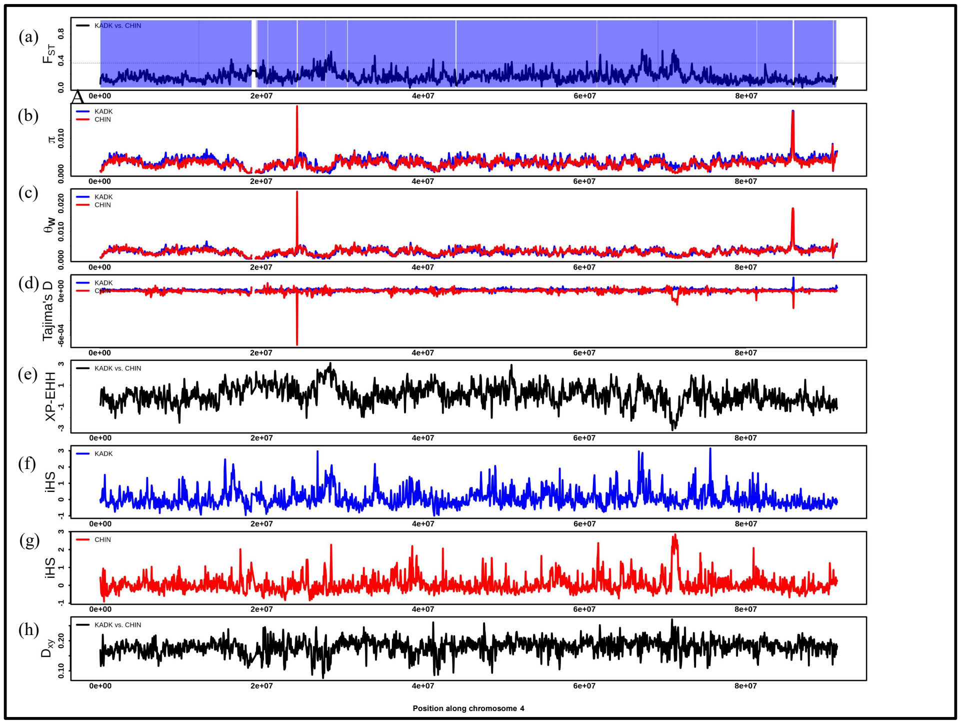

Supplementary Figure 65. (a) Pairwise F_{ST} comparison between KADK and CHIN population along chromosome 4 using 50Kb windows. A horizontal black dotted line represents the 99 percentile F_{ST} threshold. >80 percent callable region shown in transparent blue color while white color region represents <80 percent callable region. **b,c,d.** represents the π, Watterson theta, and Tajima`s D, respectively, where the solid blue line represents the KADK, and the solid red line represents the CHIN population. **(e)** Pairwise XP-EHH comparison between KADK and CHIN using 50Kb window. **f, g.** iHS results visualized in 50Kb window along the chromosome for KADK shown in solid blue color and CHIN shown in solid red color, respectively. **(h)** Dxy between KADK and CHIN.

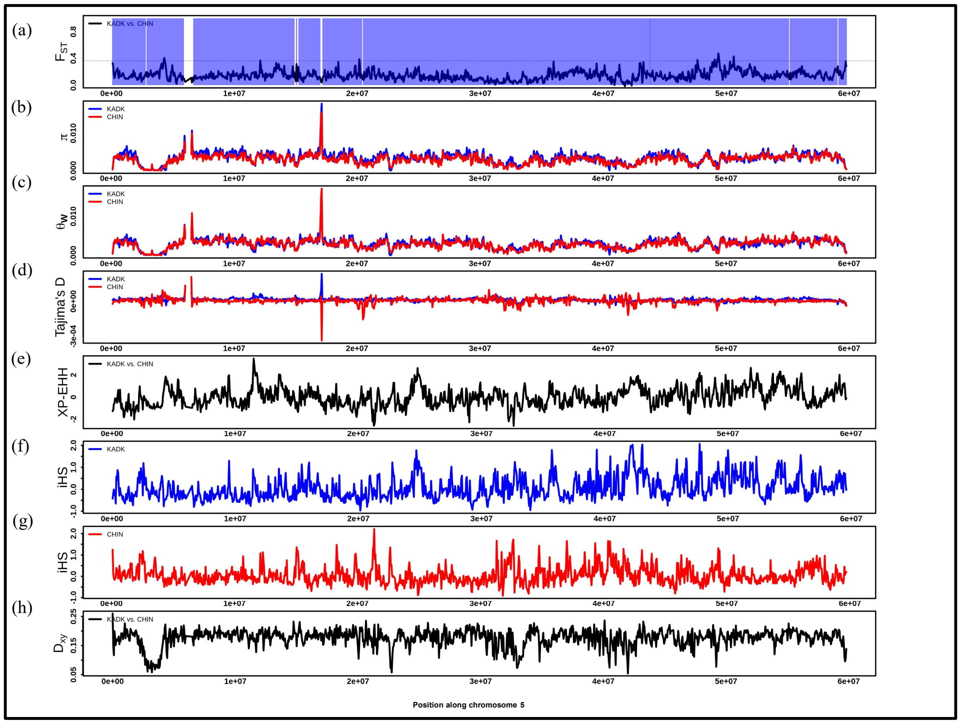

Supplementary Figure 66. (a) Pairwise F_{ST} comparison between KADK and CHIN population along chromosome 5 using 50Kb windows. A horizontal black dotted line represents the 99 percentile F_{ST} threshold. >80 percent callable region shown in transparent blue color while white color region represents <80 percent callable region. **b,c,d.** represents the π, Watterson theta, and Tajima`s D, respectively, where the solid blue line represents the KADK, and the solid red line represents the CHIN population. **(e)** Pairwise XP-EHH comparison between KADK and CHIN using 50Kb window. **f, g.** iHS results visualized in 50Kb window along the chromosome for KADK shown in solid blue color and CHIN shown in solid red color, respectively. **(h)** Dxy between KADK and CHIN.

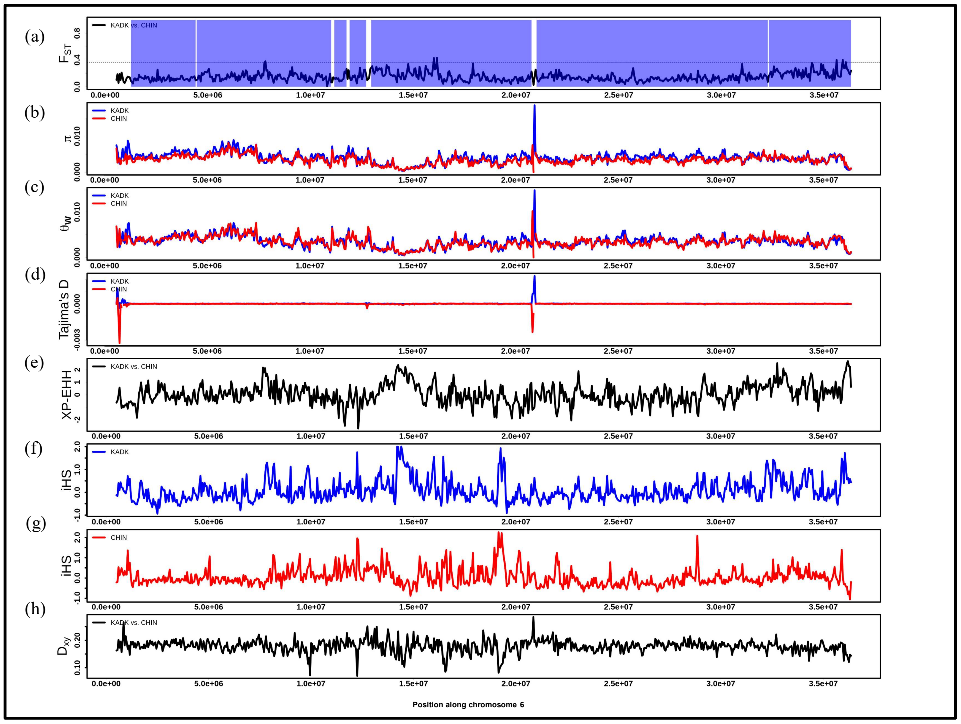

Supplementary Figure 67. (a) Pairwise F_{ST} comparison between KADK and CHIN population along chromosome 6 using 50Kb windows. A horizontal black dotted line represents the 99 percentile F_{ST} threshold. >80 percent callable region shown in transparent blue color while white color region represents <80 percent callable region. **b,c,d.** represents the π, Watterson theta, and Tajima`s D, respectively, where the solid blue line represents the KADK, and the solid red line represents the CHIN population. **(e)** Pairwise XP-EHH comparison between KADK and CHIN using 50Kb window. **f, g.** iHS results visualized in 50Kb window along the chromosome for KADK shown in solid blue color and CHIN shown in solid red color, respectively. **(h)** Dxy between KADK and CHIN.

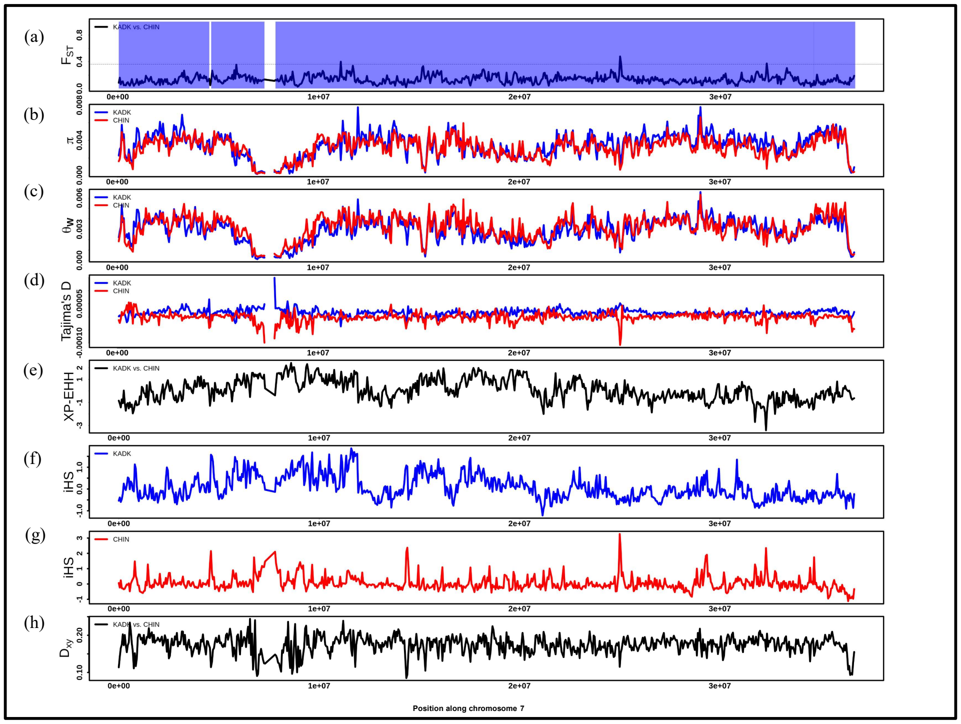

Supplementary Figure 68. (a) Pairwise F_{ST} comparison between KADK and CHIN population along chromosome 7 using 50Kb windows. A horizontal black dotted line represents the 99 percentile F_{ST} threshold. >80 percent callable region shown in transparent blue color while white color region represents <80 percent callable region. **b,c,d.** represents the π, Watterson theta, and Tajima`s D, respectively, where the solid blue line represents the KADK, and the solid red line represents the CHIN population. **(e)** Pairwise XP-EHH comparison between KADK and CHIN using 50Kb window. **f, g.** iHS results visualized in 50Kb window along the chromosome for KADK shown in solid blue color and CHIN shown in solid red color, respectively. **(h)** Dxy between KADK and CHIN.

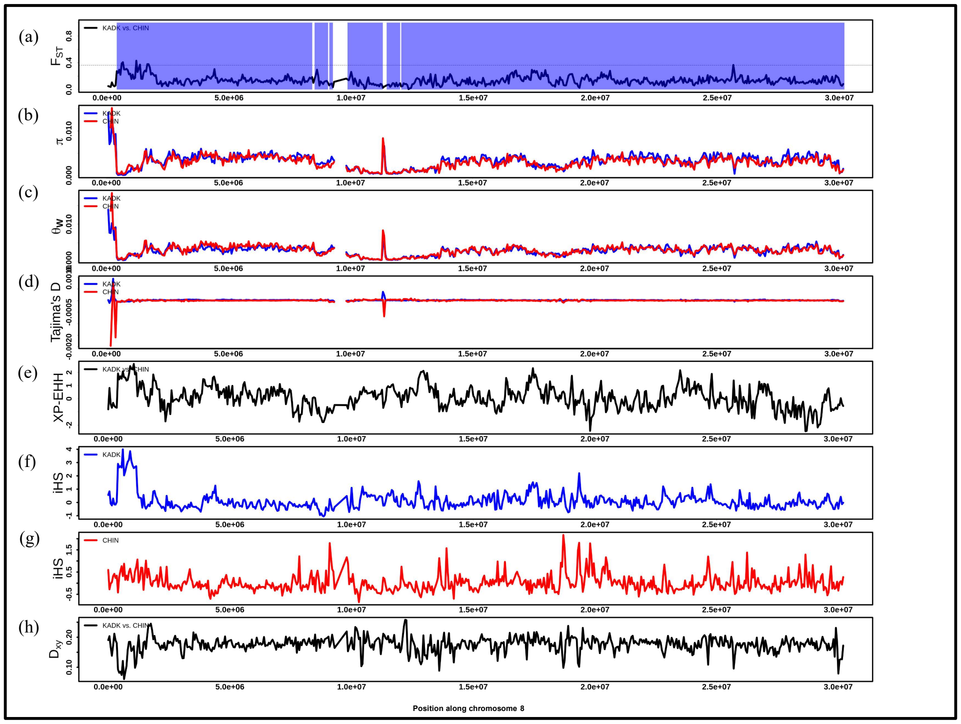

Supplementary Figure 69. (a) Pairwise F_{ST} comparison between KADK and CHIN population along chromosome 8 using 50Kb windows. A horizontal black dotted line represents the 99 percentile F_{ST} threshold. >80 percent callable region shown in transparent blue color while white color region represents <80 percent callable region. **b,c,d.** represents the π, Watterson theta, and Tajima`s D, respectively, where the solid blue line represents the KADK, and the solid red line represents the CHIN population. **(e)** Pairwise XP-EHH comparison between KADK and CHIN using 50Kb window. **f, g.** iHS results visualized in 50Kb window along the chromosome for KADK shown in solid blue color and CHIN shown in solid red color, respectively. **(h)** Dxy between KADK and CHIN.

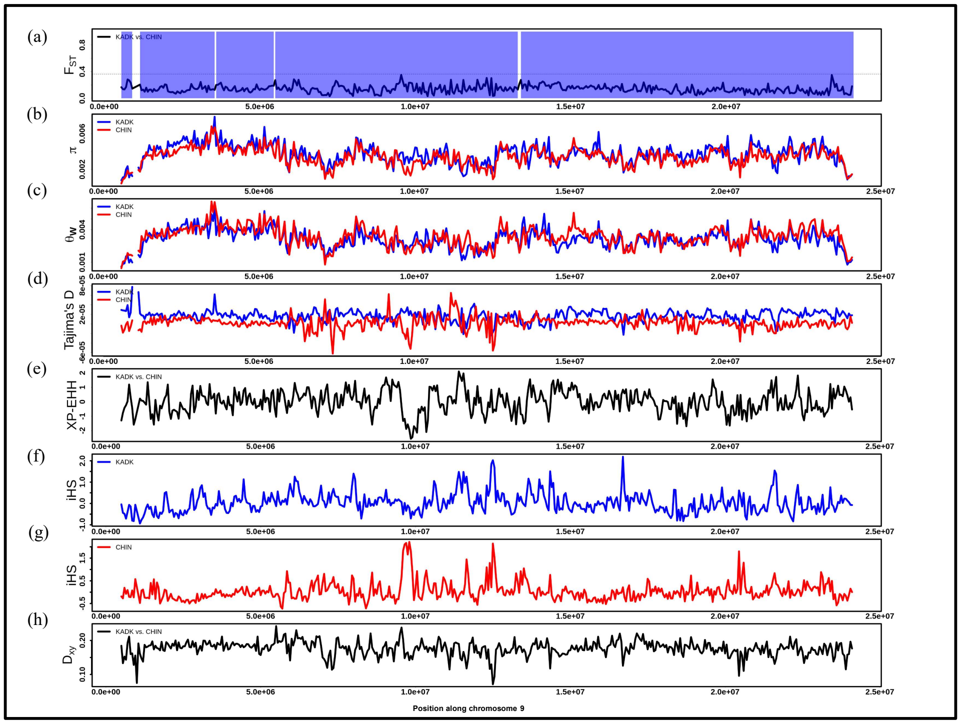

Supplementary Figure 70. (a) Pairwise F_{ST} comparison between KADK and CHIN population along chromosome 9 using 50Kb windows. A horizontal black dotted line represents the 99 percentile F_{ST} threshold. >80 percent callable region shown in transparent blue color while white color region represents <80 percent callable region. **b,c,d.** represents the π, Watterson theta, and Tajima`s D, respectively, where the solid blue line represents the KADK, and the solid red line represents the CHIN population. **(e)** Pairwise XP-EHH comparison between KADK and CHIN using 50Kb window. **f, g.** iHS results visualized in 50Kb window along the chromosome for KADK shown in solid blue color and CHIN shown in solid red color, respectively. **(h)** Dxy between KADK and CHIN.

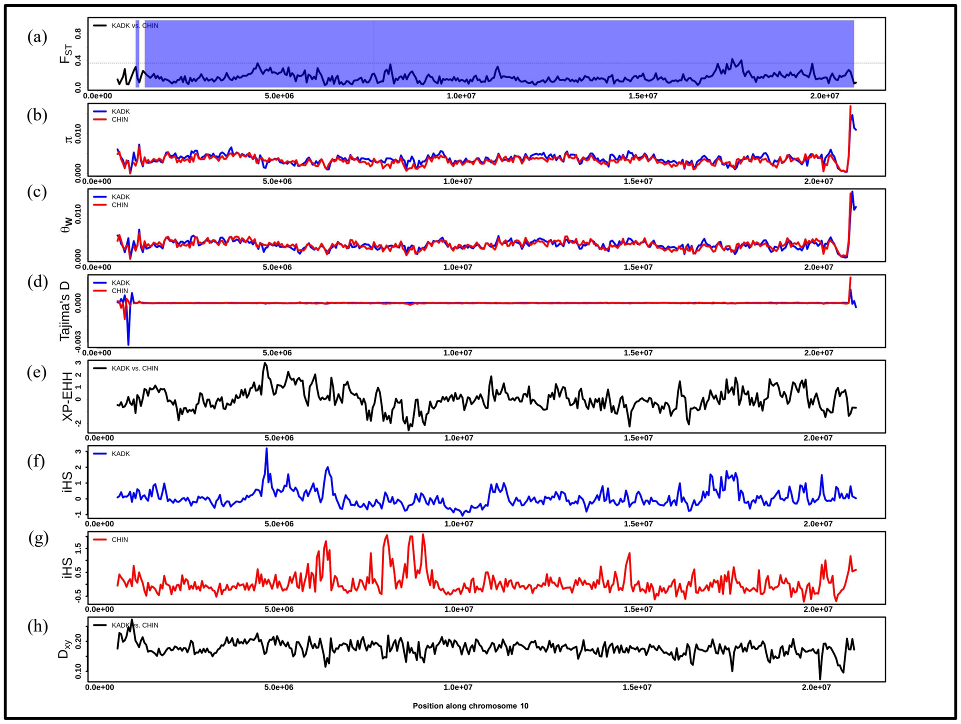

Supplementary Figure 71. (a) Pairwise F_{ST} comparison between KADK and CHIN population along chromosome 10 using 50Kb windows. A horizontal black dotted line represents the 99 percentile F_{ST} threshold. >80 percent callable region shown in transparent blue color while white color region represents <80 percent callable region. **b,c,d.** represents the π, Watterson theta, and Tajima`s D, respectively, where the solid blue line represents the KADK, and the solid red line represents the CHIN population. **(e)** Pairwise XP-EHH comparison between KADK and CHIN using 50Kb window. **f, g.** iHS results visualized in 50Kb window along the chromosome for KADK shown in solid blue color and CHIN shown in solid red color, respectively. **(h)** Dxy between KADK and CHIN.

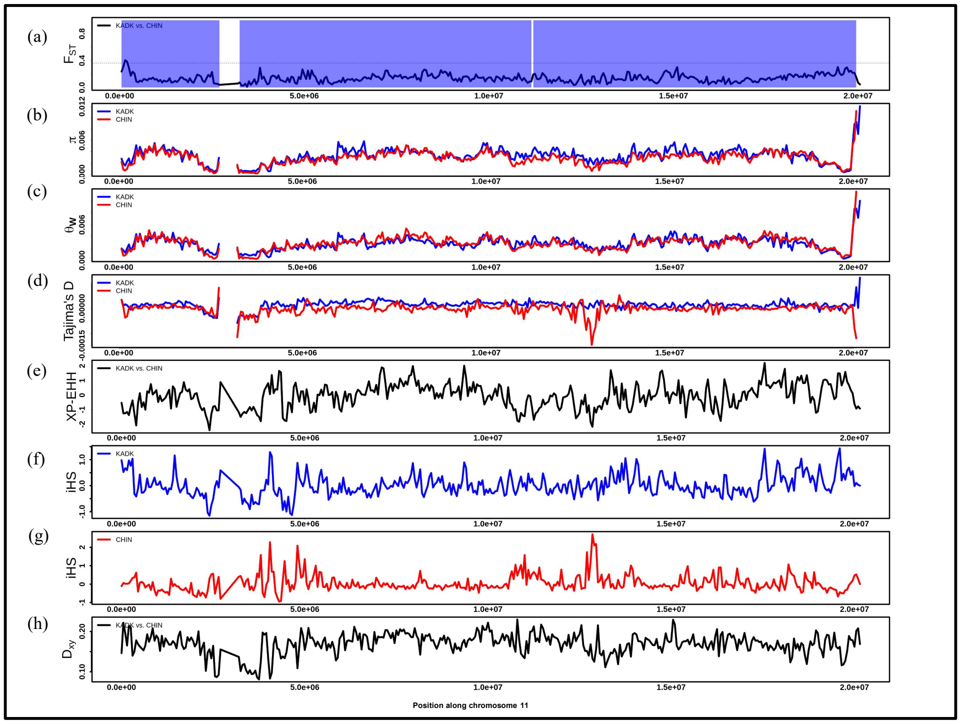

Supplementary Figure 72. (a) Pairwise F_{ST} comparison between KADK and CHIN population along chromosome 11 using 50Kb windows. A horizontal black dotted line represents the 99 percentile F_{ST} threshold. >80 percent callable region shown in transparent blue color while white color region represents <80 percent callable region. **b,c,d.** represents the π, Watterson theta, and Tajima`s D, respectively, where the solid blue line represents the KADK, and the solid red line represents the CHIN population. **(e)** Pairwise XP-EHH comparison between KADK and CHIN using 50Kb window. **f, g.** iHS results visualized in 50Kb window along the chromosome for KADK shown in solid blue color and CHIN shown in solid red color, respectively. **(h)** Dxy between KADK and CHIN.

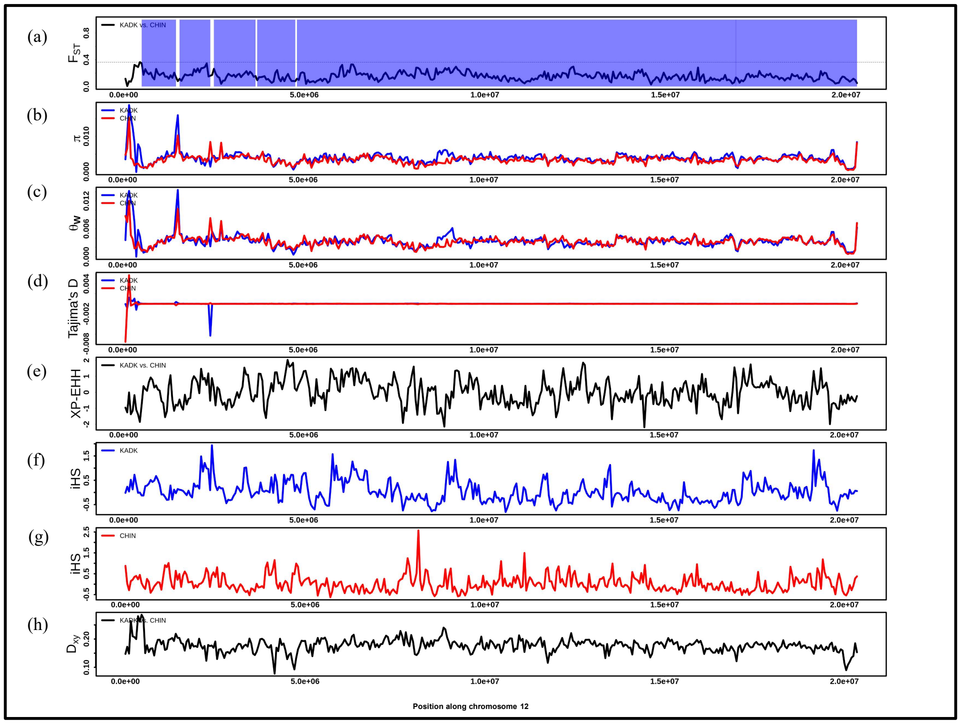

Supplementary Figure 73. (a) Pairwise F_{ST} comparison between KADK and CHIN population along chromosome 12 using 50Kb windows. A horizontal black dotted line represents the 99 percentile F_{ST} threshold. >80 percent callable region shown in transparent blue color while white color region represents <80 percent callable region. **b,c,d.** represents the π, Watterson theta, and Tajima`s D, respectively, where the solid blue line represents the KADK, and the solid red line represents the CHIN population. **(e)** Pairwise XP-EHH comparison between KADK and CHIN using 50Kb window. **f, g.** iHS results visualized in 50Kb window along the chromosome for KADK shown in solid blue color and CHIN shown in solid red color, respectively. **(h)** Dxy between KADK and CHIN.

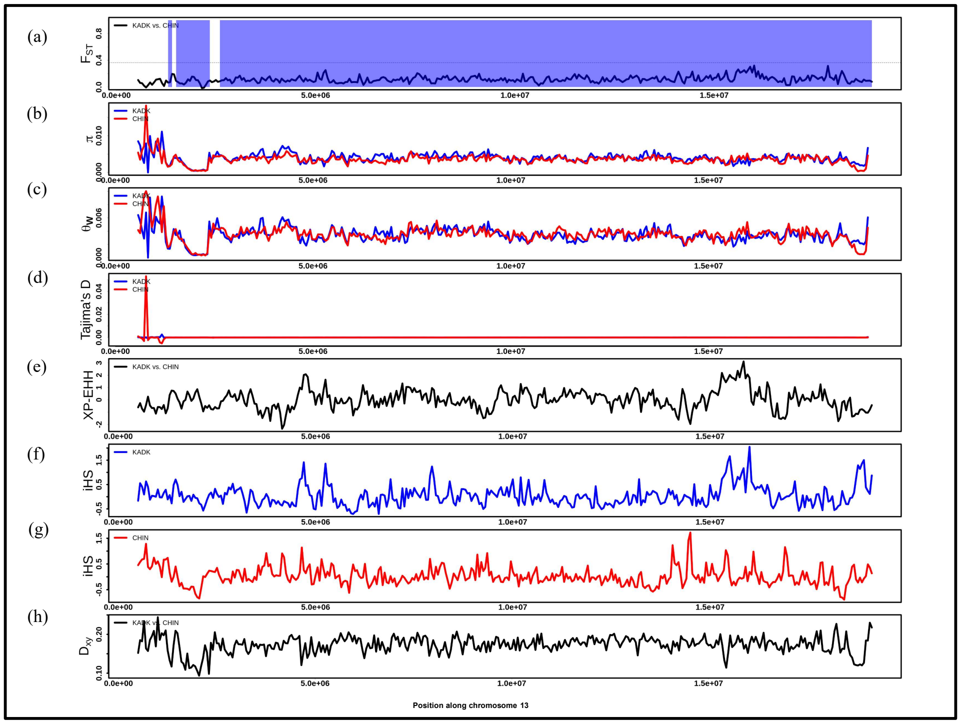

Supplementary Figure 74. (a) Pairwise F_{ST} comparison between KADK and CHIN population along chromosome 13 using 50Kb windows. A horizontal black dotted line represents the 99 percentile F_{ST} threshold. >80 percent callable region shown in transparent blue color while white color region represents <80 percent callable region. **b,c,d.** represents the π, Watterson theta, and Tajima`s D, respectively, where the solid blue line represents the KADK, and the solid red line represents the CHIN population. **(e)** Pairwise XP-EHH comparison between KADK and CHIN using 50Kb window. **f, g.** iHS results visualized in 50Kb window along the chromosome for KADK shown in solid blue color and CHIN shown in solid red color, respectively. **(h)** Dxy between KADK and CHIN.

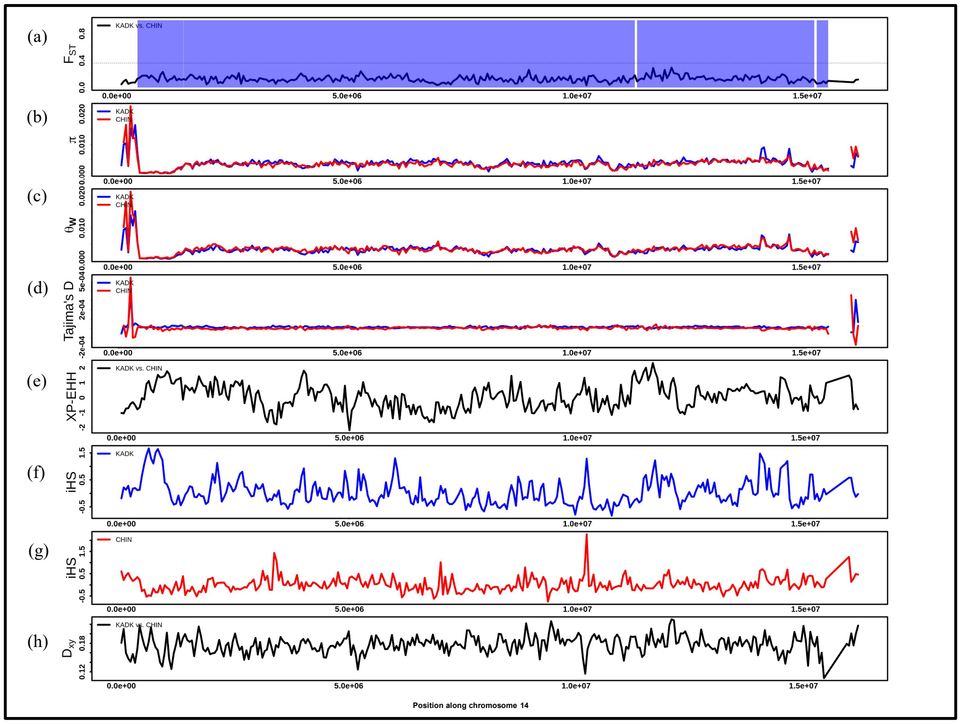

Supplementary Figure 75. (a) Pairwise F_{ST} comparison between KADK and CHIN population along chromosome 14 using 50Kb windows. A horizontal black dotted line represents the 99 percentile F_{ST} threshold. >80 percent callable region shown in transparent blue color while white color region represents <80 percent callable region. **b,c,d.** represents the π, Watterson theta, and Tajima`s D, respectively, where the solid blue line represents the KADK, and the solid red line represents the CHIN population. **(e)** Pairwise XP-EHH comparison between KADK and CHIN using 50Kb window. **f, g.** iHS results visualized in 50Kb window along the chromosome for KADK shown in solid blue color and CHIN shown in solid red color, respectively. **(h)** Dxy between KADK and CHIN.

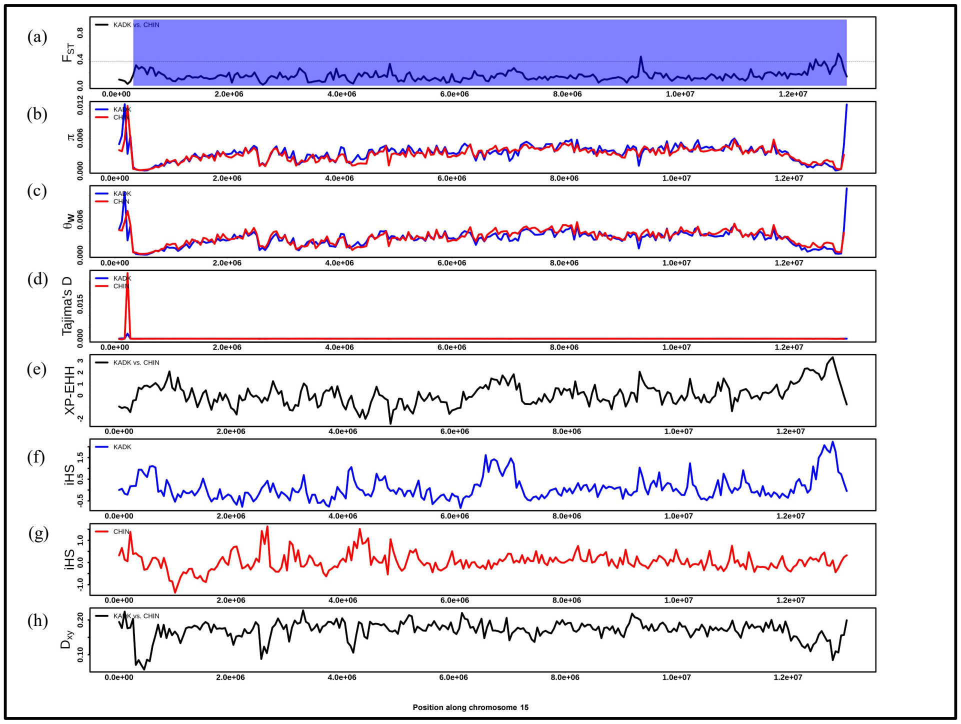

Supplementary Figure 76. (a) Pairwise F_{ST} comparison between KADK and CHIN population along chromosome 15 using 50Kb windows. A horizontal black dotted line represents the 99 percentile F_{ST} threshold. >80 percent callable region shown in transparent blue color while white color region represents <80 percent callable region. **b,c,d.** represents the π, Watterson theta, and Tajima`s D, respectively, where the solid blue line represents the KADK, and the solid red line represents the CHIN population. **(e)** Pairwise XP-EHH comparison between KADK and CHIN using 50Kb window. **f, g.** iHS results visualized in 50Kb window along the chromosome for KADK shown in solid blue color and CHIN shown in solid red color, respectively. **(h)** Dxy between KADK and CHIN.

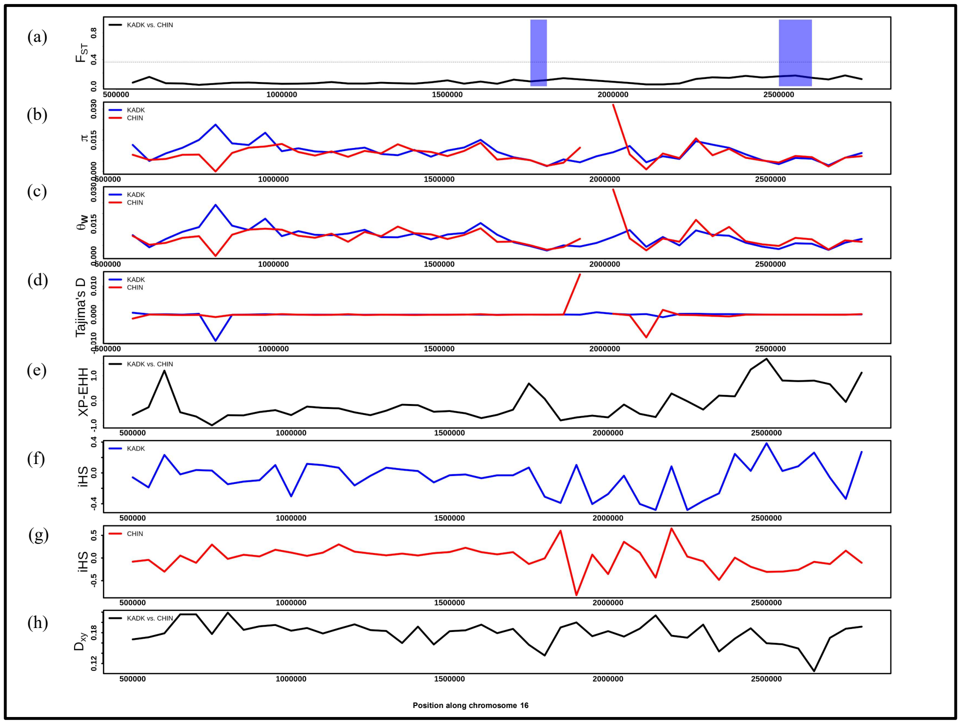

Supplementary Figure 77. (a) Pairwise F_{ST} comparison between KADK and CHIN population along chromosome 16 using 50Kb windows. A horizontal black dotted line represents the 99 percentile F_{ST} threshold. >80 percent callable region shown in transparent blue color while white color region represents <80 percent callable region. **b,c,d.** represents the π, Watterson theta, and Tajima`s D, respectively, where the solid blue line represents the KADK, and the solid red line represents the CHIN population. **(e)** Pairwise XP-EHH comparison between KADK and CHIN using 50Kb window. **f, g.** iHS results visualized in 50Kb window along the chromosome for KADK shown in solid blue color and CHIN shown in solid red color, respectively. **(h)** Dxy between KADK and CHIN.

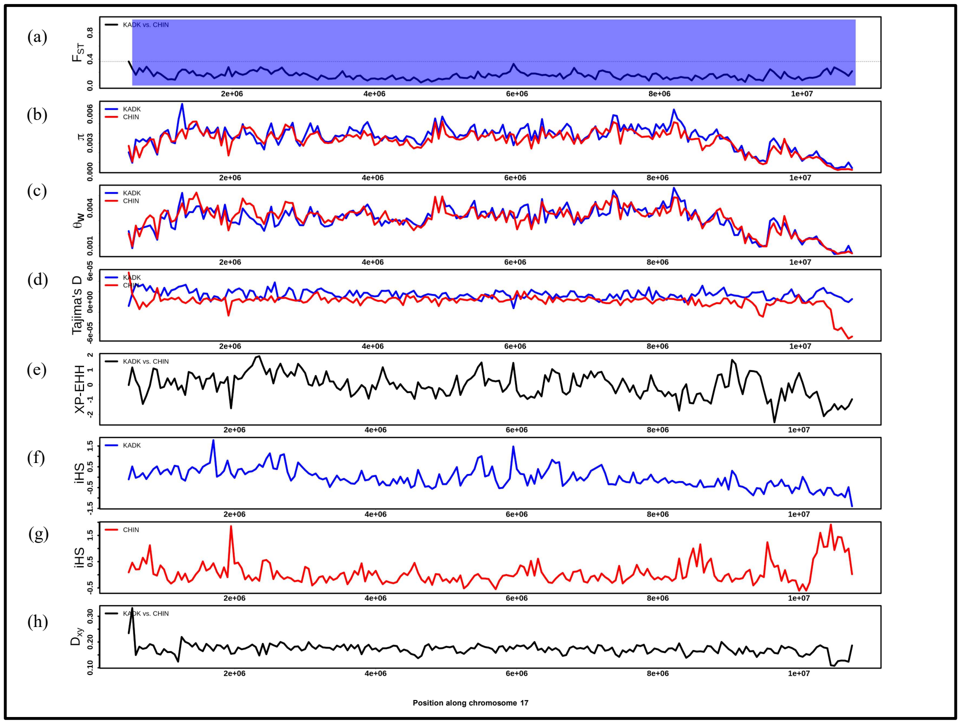

Supplementary Figure 78. (a) Pairwise F_{ST} comparison between KADK and CHIN population along chromosome 17 using 50Kb windows. A horizontal black dotted line represents the 99 percentile F_{ST} threshold. >80 percent callable region shown in transparent blue color while white color region represents <80 percent callable region. **b,c,d.** represents the π, Watterson theta, and Tajima`s D, respectively, where the solid blue line represents the KADK, and the solid red line represents the CHIN population. **(e)** Pairwise XP-EHH comparison between KADK and CHIN using 50Kb window. **f, g.** iHS results visualized in 50Kb window along the chromosome for KADK shown in solid blue color and CHIN shown in solid red color, respectively. **(h)** Dxy between KADK and CHIN.

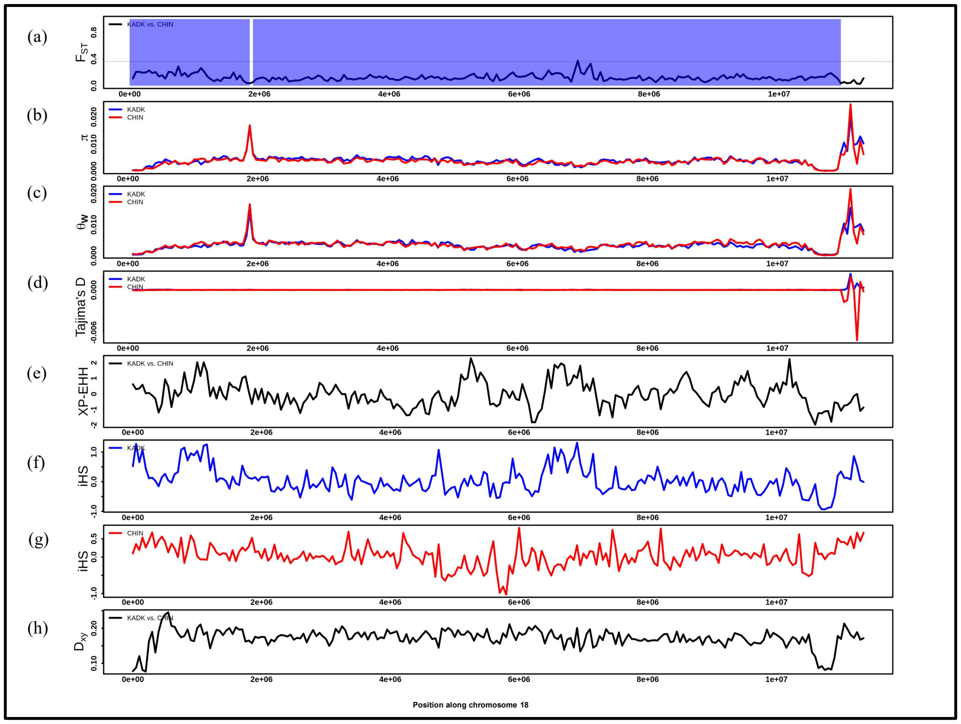

Supplementary Figure 79. (a) Pairwise F_{ST} comparison between KADK and CHIN population along chromosome 18 using 50Kb windows. A horizontal black dotted line represents the 99 percentile F_{ST} threshold. >80 percent callable region shown in transparent blue color while white color region represents <80 percent callable region. **b,c,d.** represents the π, Watterson theta, and Tajima`s D, respectively, where the solid blue line represents the KADK, and the solid red line represents the CHIN population. **(e)** Pairwise XP-EHH comparison between KADK and CHIN using 50Kb window. **f, g.** iHS results visualized in 50Kb window along the chromosome for KADK shown in solid blue color and CHIN shown in solid red color, respectively. **(h)** Dxy between KADK and CHIN.

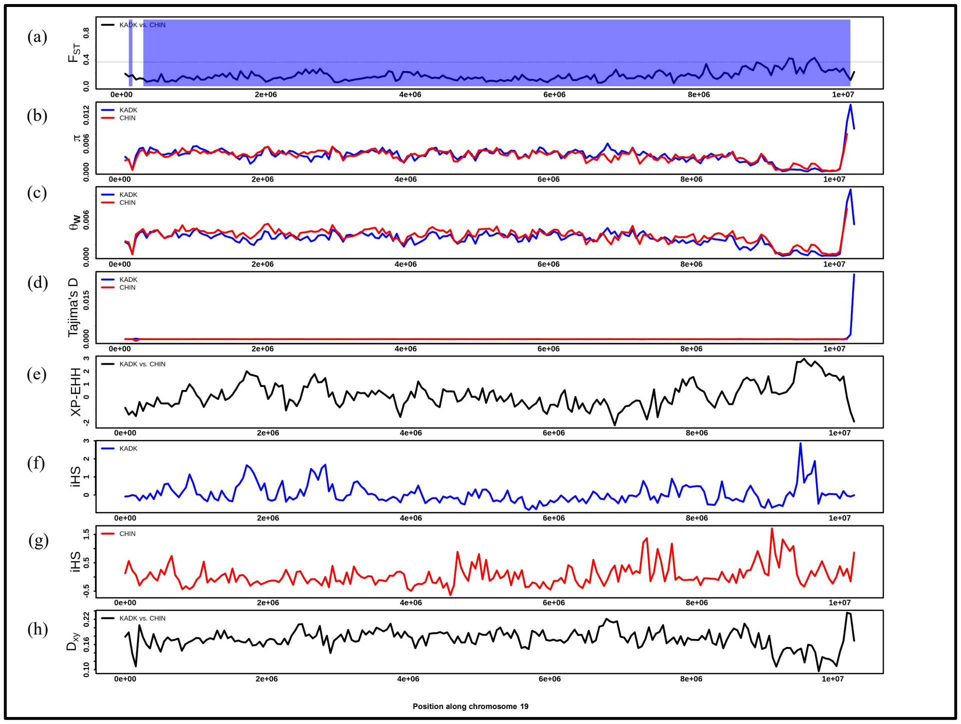

Supplementary Figure 80. (a) Pairwise F_{ST} comparison between KADK and CHIN population along chromosome 19 using 50Kb windows. A horizontal black dotted line represents the 99 percentile F_{ST} threshold. >80 percent callable region shown in transparent blue color while white color region represents <80 percent callable region. **b,c,d.** represents the π, Watterson theta, and Tajima`s D, respectively, where the solid blue line represents the KADK, and the solid red line represents the CHIN population. **(e)** Pairwise XP-EHH comparison between KADK and CHIN using 50Kb window. **f, g.** iHS results visualized in 50Kb window along the chromosome for KADK shown in solid blue color and CHIN shown in solid red color, respectively. **(h)** Dxy between KADK and CHIN.

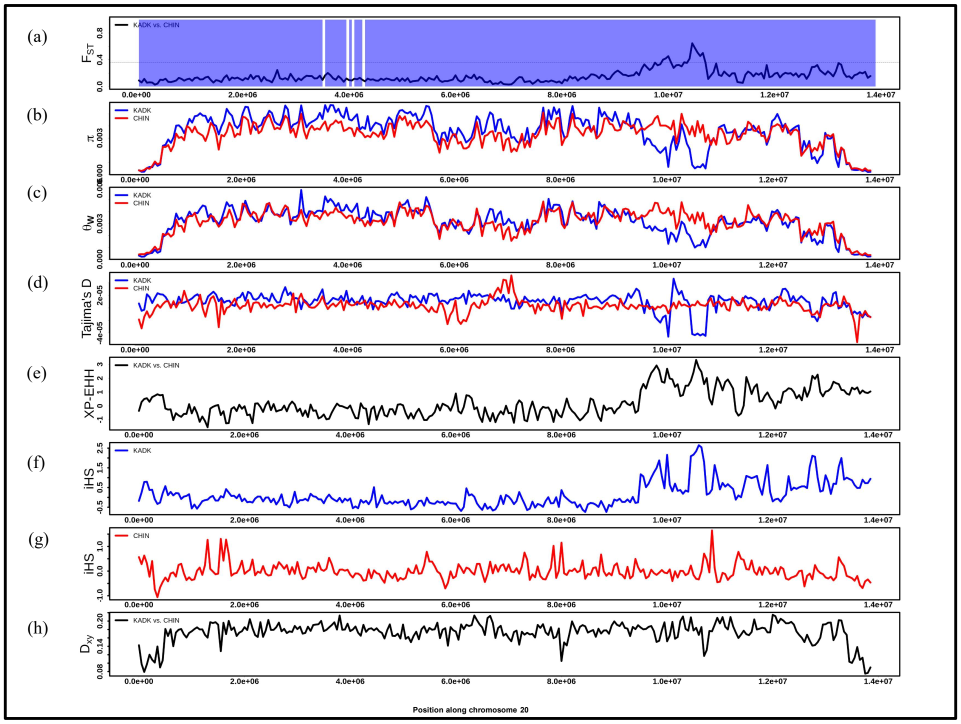

Supplementary Figure 81. (a) Pairwise F_{ST} comparison between KADK and CHIN population along chromosome 20 using 50Kb windows. A horizontal black dotted line represents the 99 percentile F_{ST} threshold. >80 percent callable region shown in transparent blue color while white color region represents <80 percent callable region. **b,c,d.** represents the π, Watterson theta, and Tajima`s D, respectively, where the solid blue line represents the KADK, and the solid red line represents the CHIN population. **(e)** Pairwise XP-EHH comparison between KADK and CHIN using 50Kb window. **f, g.** iHS results visualized in 50Kb window along the chromosome for KADK shown in solid blue color and CHIN shown in solid red color, respectively. **(h)** Dxy between KADK and CHIN.

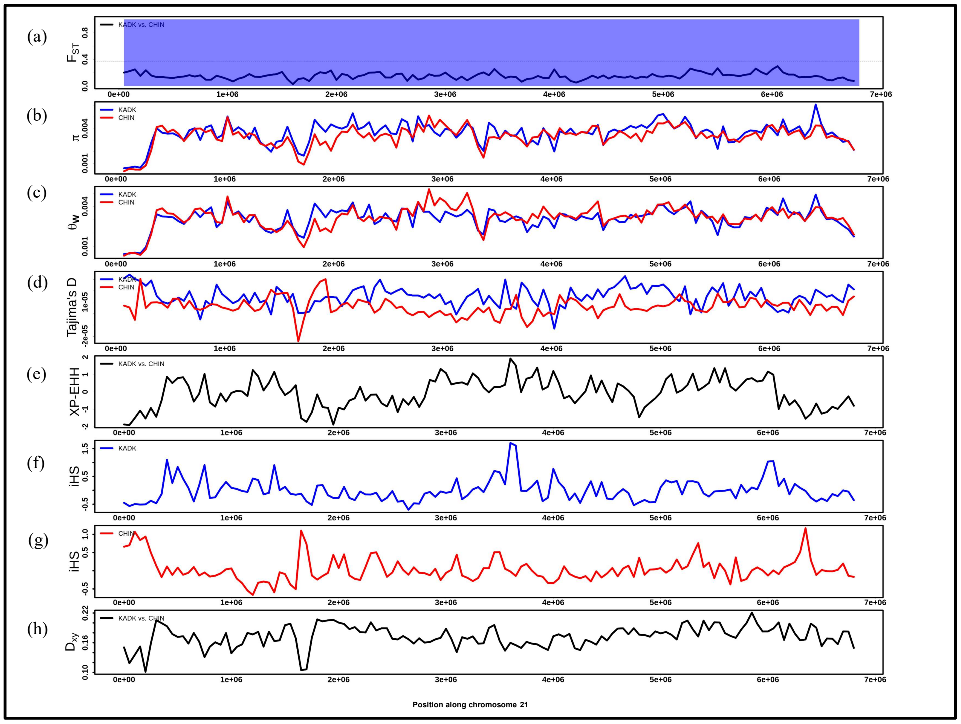

Supplementary Figure 82. (a) Pairwise F_{ST} comparison between KADK and CHIN population along chromosome 21 using 50Kb windows. A horizontal black dotted line represents the 99 percentile F_{ST} threshold. >80 percent callable region shown in transparent blue color while white color region represents <80 percent callable region. **b,c,d.** represents the π, Watterson theta, and Tajima`s D, respectively, where the solid blue line represents the KADK, and the solid red line represents the CHIN population. **(e)** Pairwise XP-EHH comparison between KADK and CHIN using 50Kb window. **f, g.** iHS results visualized in 50Kb window along the chromosome for KADK shown in solid blue color and CHIN shown in solid red color, respectively. **(h)** Dxy between KADK and CHIN.

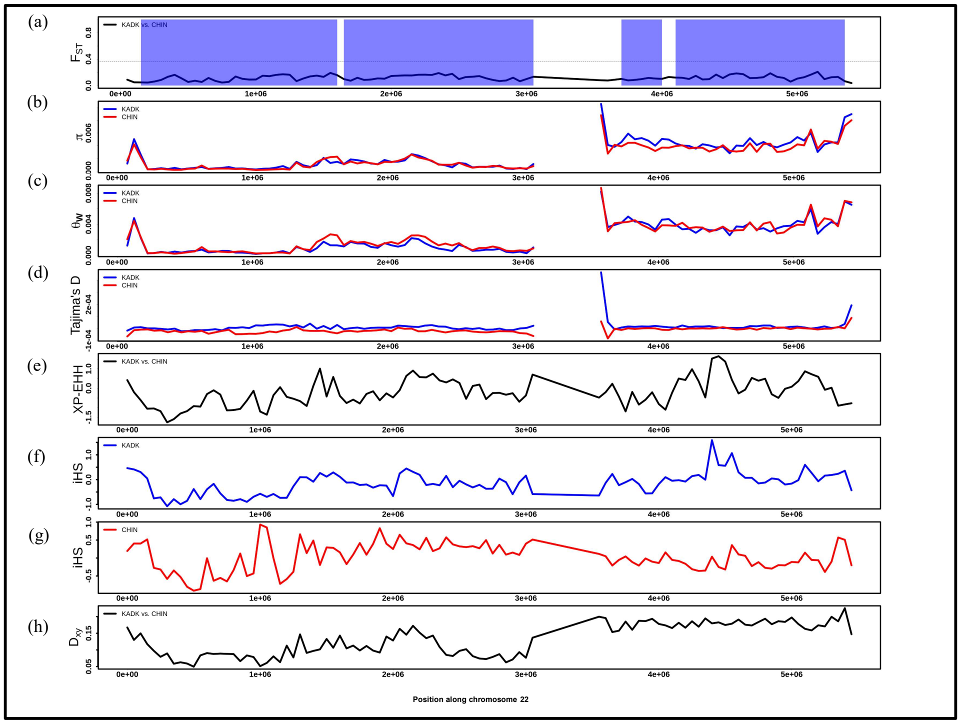

Supplementary Figure 83. (a) Pairwise F_{ST} comparison between KADK and CHIN population along chromosome 22 using 50Kb windows. A horizontal black dotted line represents the 99 percentile F_{ST} threshold. >80 percent callable region shown in transparent blue color while white color region represents <80 percent callable region. **b,c,d.** represents the π, Watterson theta, and Tajima`s D, respectively, where the solid blue line represents the KADK, and the solid red line represents the CHIN population. **(e)** Pairwise XP-EHH comparison between KADK and CHIN using 50Kb window. **f, g.** iHS results visualized in 50Kb window along the chromosome for KADK shown in solid blue color and CHIN shown in solid red color, respectively. **(h)** Dxy between KADK and CHIN.

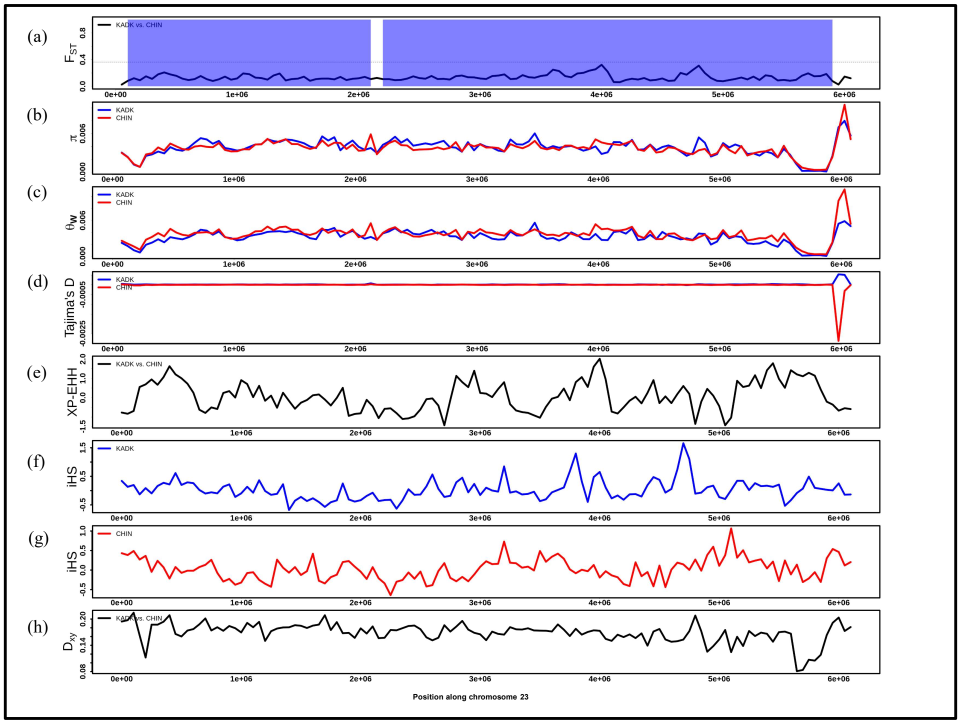

Supplementary Figure 84. (a) Pairwise F_{ST} comparison between KADK and CHIN population along chromosome 23 using 50Kb windows. A horizontal black dotted line represents the 99 percentile F_{ST} threshold. >80 percent callable region shown in transparent blue color while white color region represents <80 percent callable region. **b,c,d.** represents the π, Watterson theta, and Tajima`s D, respectively, where the solid blue line represents the KADK, and the solid red line represents the CHIN population. **(e)** Pairwise XP-EHH comparison between KADK and CHIN using 50Kb window. **f, g.** iHS results visualized in 50Kb window along the chromosome for KADK shown in solid blue color and CHIN shown in solid red color, respectively. **(h)** Dxy between KADK and CHIN.

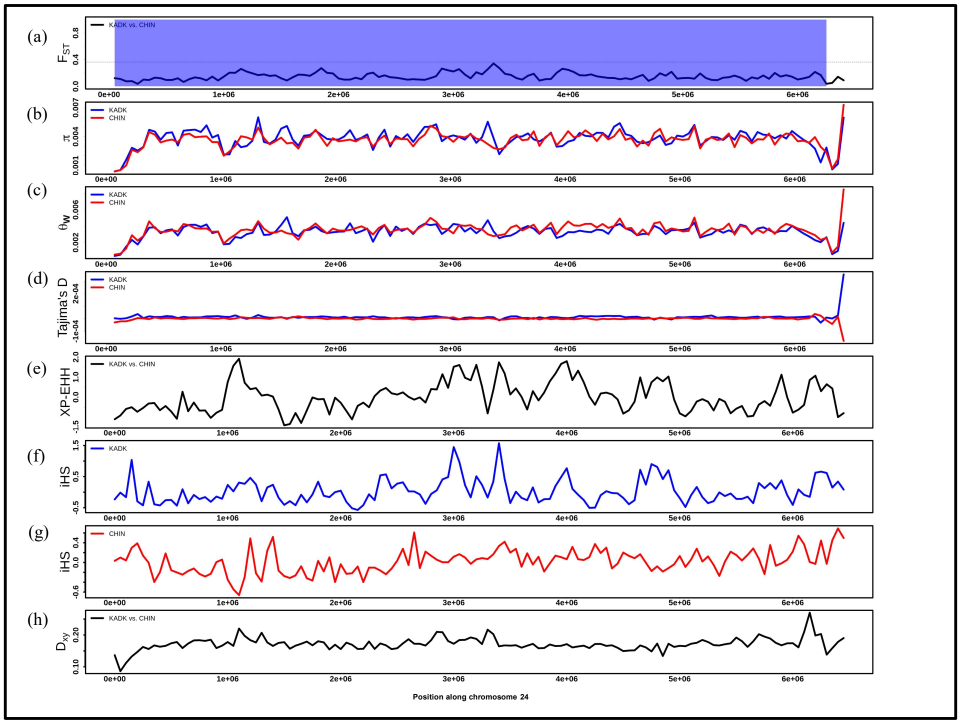

Supplementary Figure 85. (a) Pairwise F_{ST} comparison between KADK and CHIN population along chromosome 24 using 50Kb windows. A horizontal black dotted line represents the 99 percentile F_{ST} threshold. >80 percent callable region shown in transparent blue color while white color region represents <80 percent callable region. **b,c,d.** represents the π, Watterson theta, and Tajima`s D, respectively, where the solid blue line represents the KADK, and the solid red line represents the CHIN population. **(e)** Pairwise XP-EHH comparison between KADK and CHIN using 50Kb window. **f, g.** iHS results visualized in 50Kb window along the chromosome for KADK shown in solid blue color and CHIN shown in solid red color, respectively. **(h)** Dxy between KADK and CHIN.

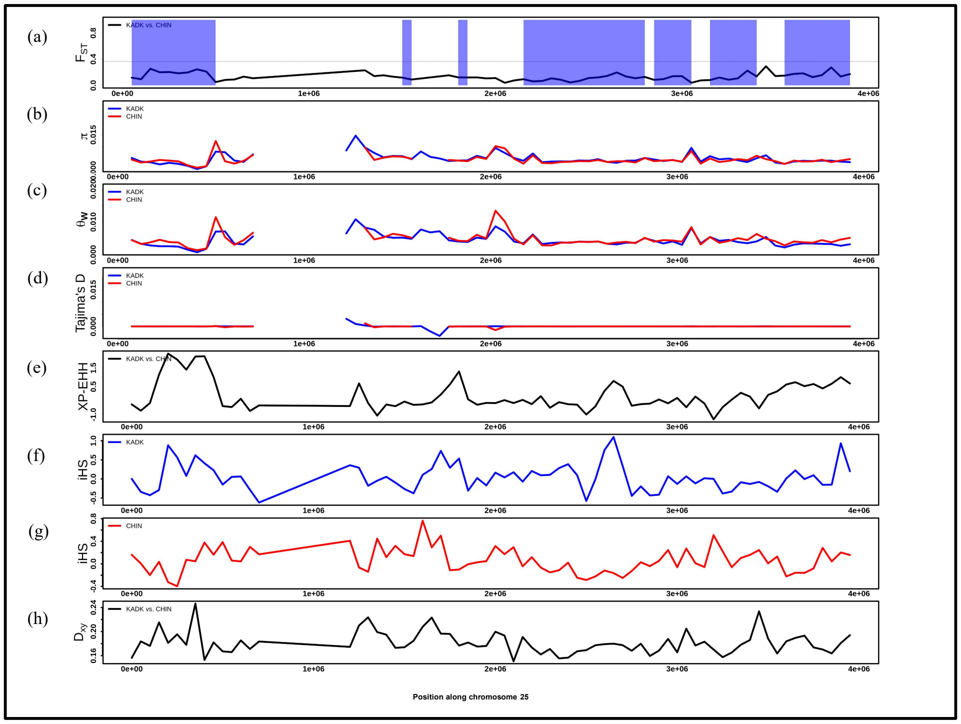

Supplementary Figure 86. (a) Pairwise F_{ST} comparison between KADK and CHIN population along chromosome 25 using 50Kb windows. A horizontal black dotted line represents the 99 percentile F_{ST} threshold. >80 percent callable region shown in transparent blue color while white color region represents <80 percent callable region. **b,c,d.** represents the π, Watterson theta, and Tajima`s D, respectively, where the solid blue line represents the KADK, and the solid red line represents the CHIN population. **(e)** Pairwise XP-EHH comparison between KADK and CHIN using 50Kb window. **f, g.** iHS results visualized in 50Kb window along the chromosome for KADK shown in solid blue color and CHIN shown in solid red color, respectively. **(h)** Dxy between KADK and CHIN.

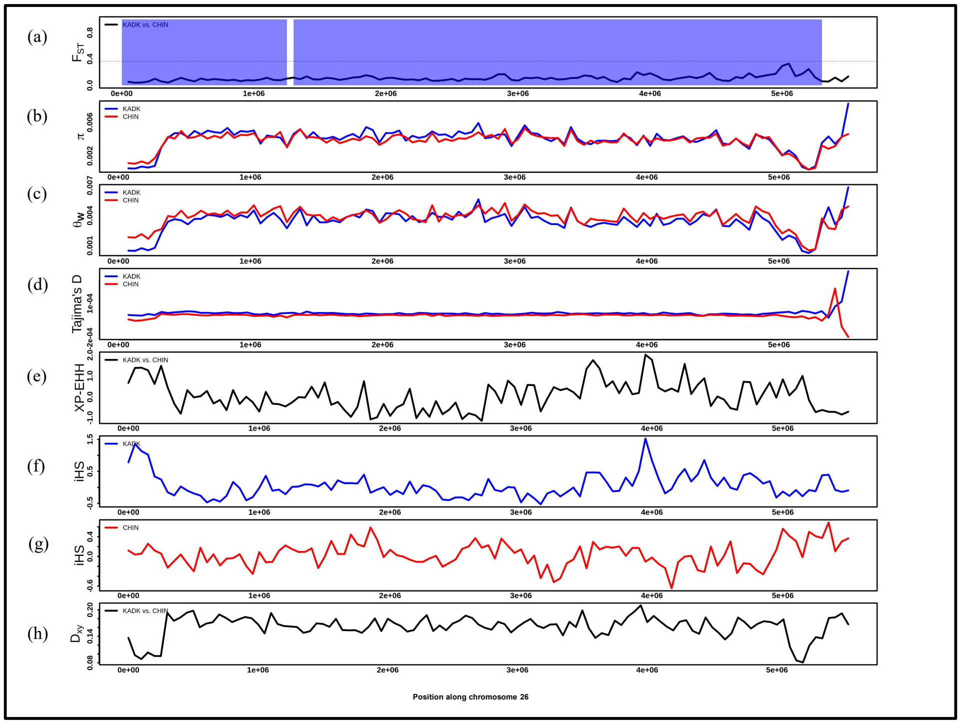

Supplementary Figure 87. (a) Pairwise F_{ST} comparison between KADK and CHIN population along chromosome 26 using 50Kb windows. A horizontal black dotted line represents the 99 percentile F_{ST} threshold. >80 percent callable region shown in transparent blue color while white color region represents <80 percent callable region. **b,c,d.** represents the π, Watterson theta, and Tajima`s D, respectively, where the solid blue line represents the KADK, and the solid red line represents the CHIN population. **(e)** Pairwise XP-EHH comparison between KADK and CHIN using 50Kb window. **f, g.** iHS results visualized in 50Kb window along the chromosome for KADK shown in solid blue color and CHIN shown in solid red color, respectively. **(h)** Dxy between KADK and CHIN.

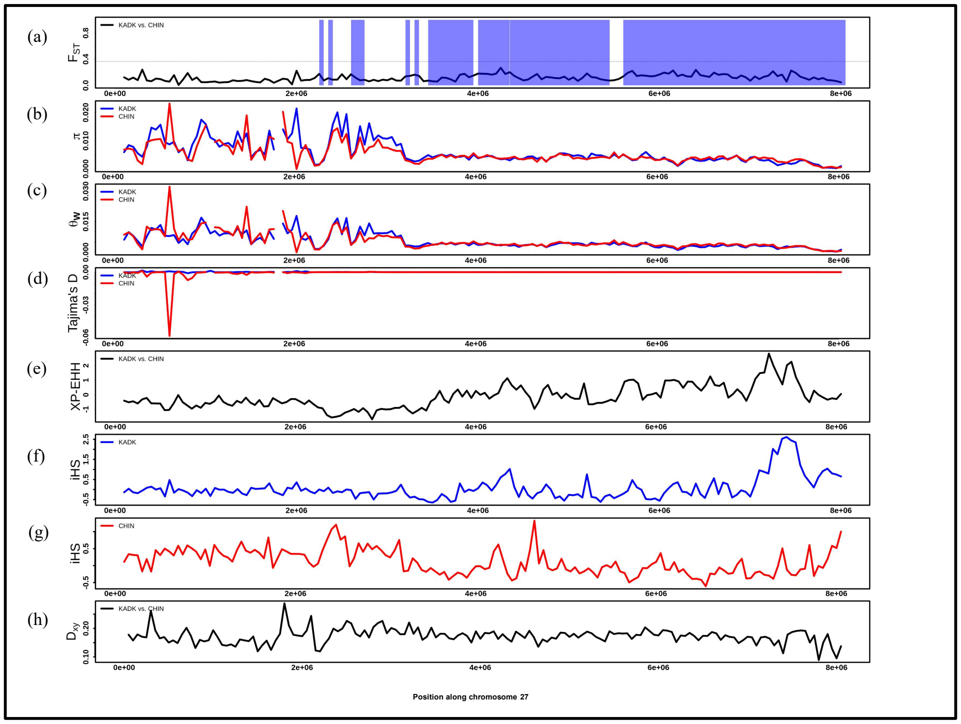

Supplementary Figure 88. (a) Pairwise F_{ST} comparison between KADK and CHIN population along chromosome 27 using 50Kb windows. A horizontal black dotted line represents the 99 percentile F_{ST} threshold. >80 percent callable region shown in transparent blue color while white color region represents <80 percent callable region. **b,c,d.** represents the π, Watterson theta, and Tajima`s D, respectively, where the solid blue line represents the KADK, and the solid red line represents the CHIN population. **(e)** Pairwise XP-EHH comparison between KADK and CHIN using 50Kb window. **f, g.** iHS results visualized in 50Kb window along the chromosome for KADK shown in solid blue color and CHIN shown in solid red color, respectively. **(h)** Dxy between KADK and CHIN.

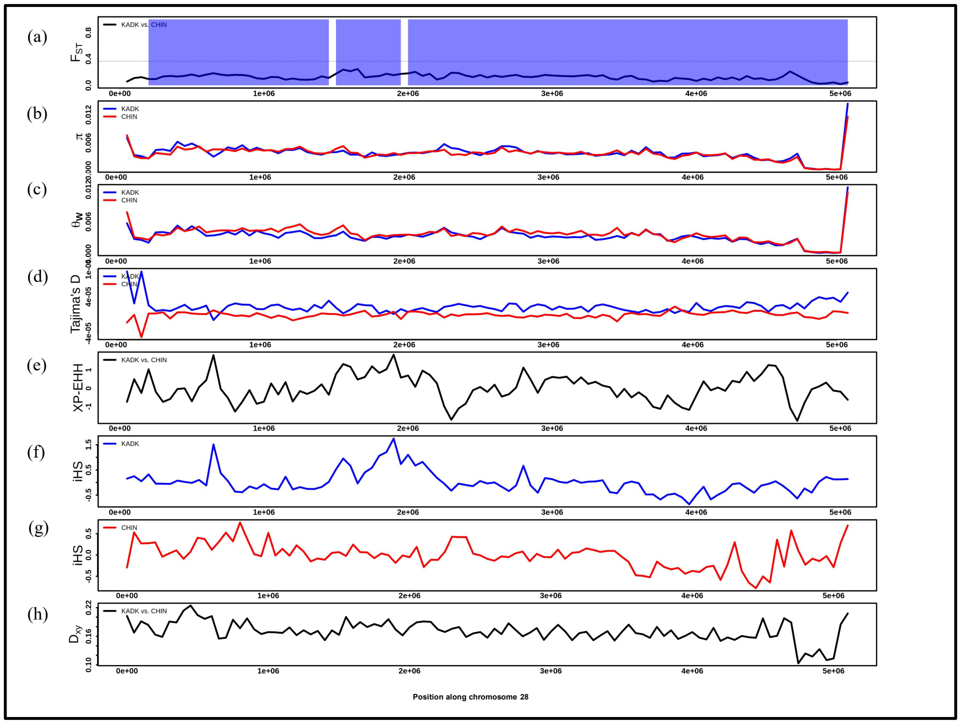

Supplementary Figure 89. (a) Pairwise F_{ST} comparison between KADK and CHIN population along chromosome 28 using 50Kb windows. A horizontal black dotted line represents the 99 percentile F_{ST} threshold. >80 percent callable region shown in transparent blue color while white color region represents <80 percent callable region. **b,c,d.** represents the π, Watterson theta, and Tajima`s D, respectively, where the solid blue line represents the KADK, and the solid red line represents the CHIN population. **(e)** Pairwise XP-EHH comparison between KADK and CHIN using 50Kb window. **f, g.** iHS results visualized in 50Kb window along the chromosome for KADK shown in solid blue color and CHIN shown in solid red color, respectively. **(h)** Dxy between KADK and CHIN.

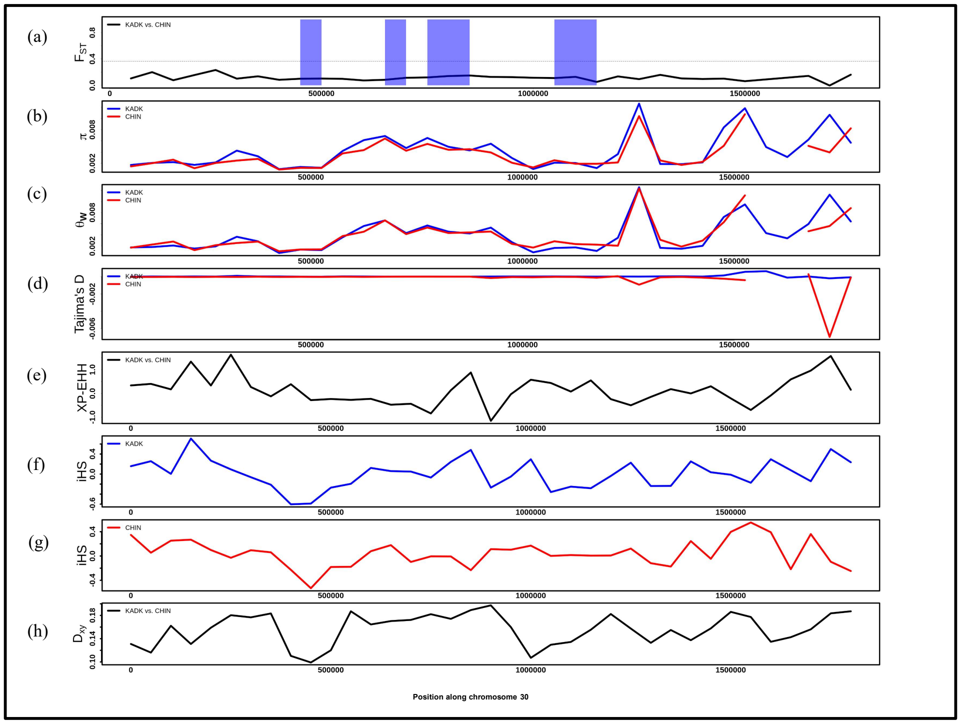

Supplementary Figure 90. (a) Pairwise F_{ST} comparison between KADK and CHIN population along chromosome 30 using 50Kb windows. A horizontal black dotted line represents the 99 percentile F_{ST} threshold. >80 percent callable region shown in transparent blue color while white color region represents <80 percent callable region. **b,c,d.** represents the π, Watterson theta, and Tajima`s D, respectively, where the solid blue line represents the KADK, and the solid red line represents the CHIN population. **(e)** Pairwise XP-EHH comparison between KADK and CHIN using 50Kb window. **f, g.** iHS results visualized in 50Kb window along the chromosome for KADK shown in solid blue color and CHIN shown in solid red color, respectively. **(h)** Dxy between KADK and CHIN.

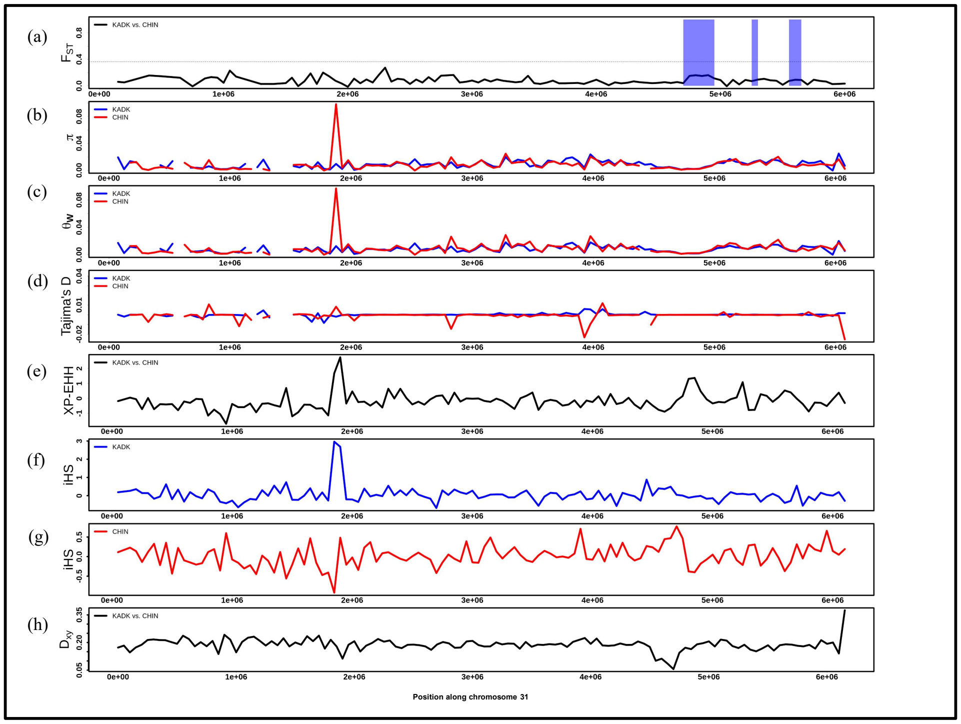

Supplementary Figure 91. (a) Pairwise F_{ST} comparison between KADK and CHIN population along chromosome 31 using 50Kb windows. A horizontal black dotted line represents the 99 percentile F_{ST} threshold. >80 percent callable region shown in transparent blue color while white color region represents <80 percent callable region. **b,c,d.** represents the π, Watterson theta, and Tajima`s D, respectively, where the solid blue line represents the KADK, and the solid red line represents the CHIN population. **(e)** Pairwise XP-EHH comparison between KADK and CHIN using 50Kb window. **f, g.** iHS results visualized in 50Kb window along the chromosome for KADK shown in solid blue color and CHIN shown in solid red color, respectively. **(h)** Dxy between KADK and CHIN.

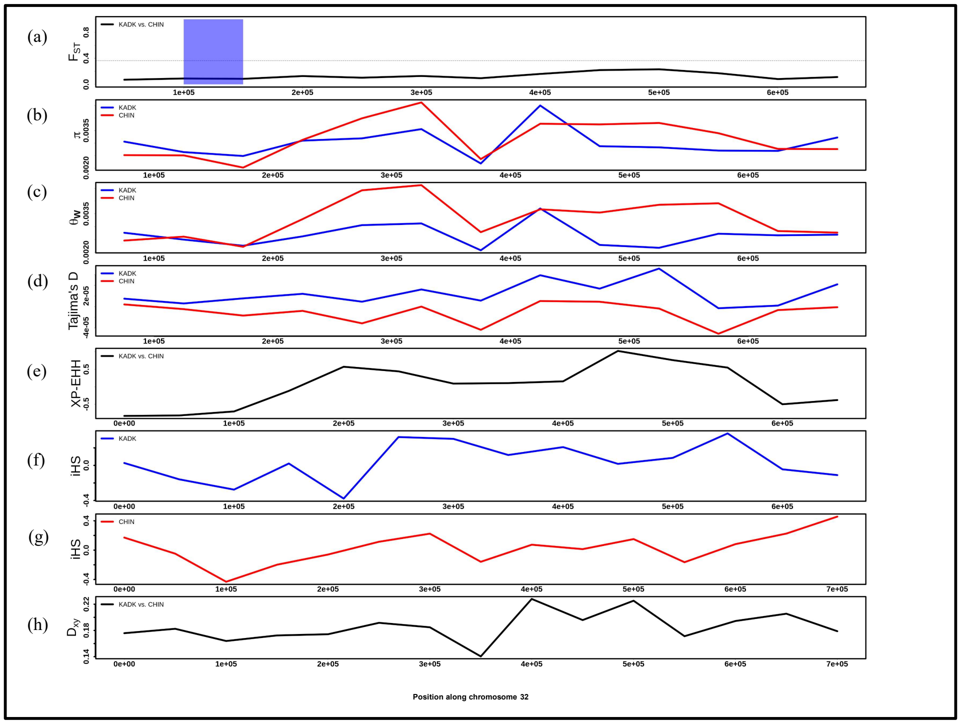

Supplementary Figure 92. **(a)** Pairwise F_{ST} comparison between KADK and CHIN population along chromosome 32 using 50Kb windows. A horizontal black dotted line represents the 99 percentile F_{ST} threshold. >80 percent callable region shown in transparent blue color while white color region represents <80 percent callable region. **b,c,d.** represents the π, Watterson theta, and Tajima`s D, respectively, where the solid blue line represents the KADK, and the solid red line represents the CHIN population. **(e)** Pairwise XP-EHH comparison between KADK and CHIN using 50Kb window. **f, g.** iHS results visualized in 50Kb window along the chromosome for KADK shown in solid blue color and CHIN shown in solid red color, respectively. **(h)** Dxy between KADK and CHIN.

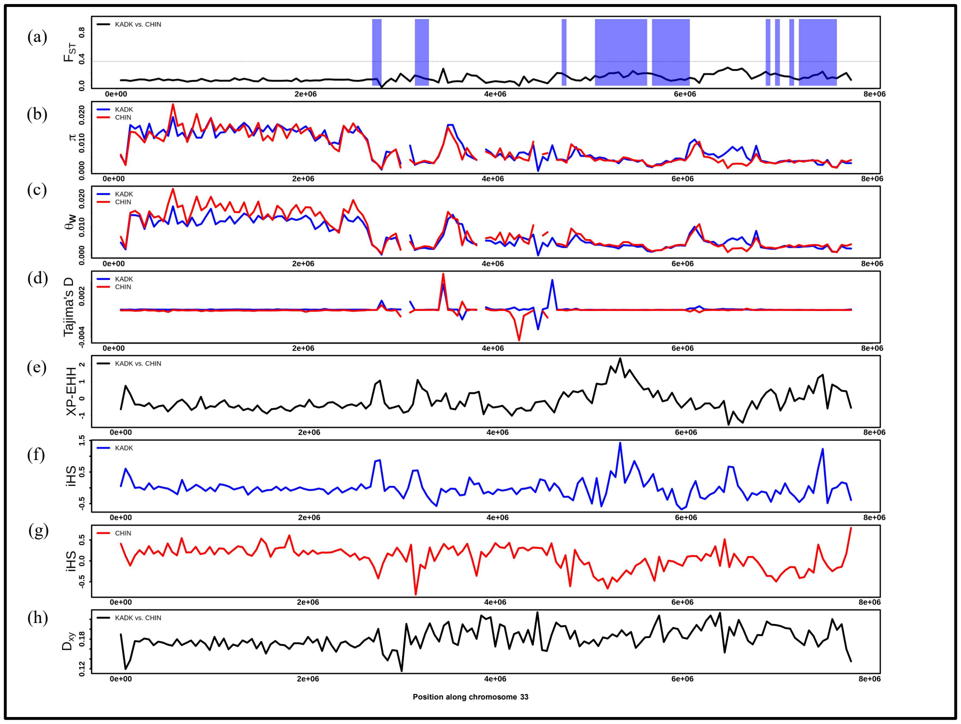

Supplementary Figure 93. (a) Pairwise F_{ST} comparison between KADK and CHIN population along chromosome 33 using 50Kb windows. A horizontal black dotted line represents the 99 percentile F_{ST} threshold. >80 percent callable region shown in transparent blue color while white color region represents <80 percent callable region. **b,c,d.** represents the π, Watterson theta, and Tajima`s D, respectively, where the solid blue line represents the KADK, and the solid red line represents the CHIN population. **(e)** Pairwise XP-EHH comparison between KADK and CHIN using 50Kb window. **f, g.** iHS results visualized in 50Kb window along the chromosome for KADK shown in solid blue color and CHIN shown in solid red color, respectively. **(h)** Dxy between KADK and CHIN.

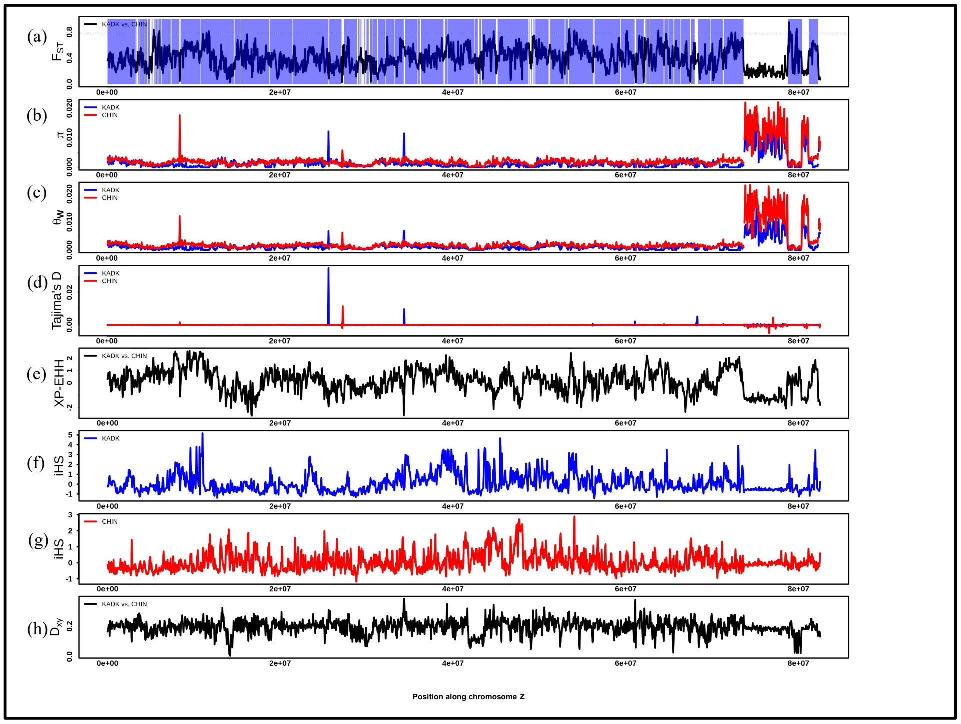

Supplementary Figure 94. (a) Pairwise F_{ST} comparison between KADK and CHIN population along chromosome Z using 50Kb windows. A horizontal black dotted line represents the 99 percentile F_{ST} threshold. >80 percent callable region shown in transparent blue color while white color region represents <80 percent callable region. **b,c,d.** represents the π, Watterson theta, and Tajima`s D, respectively, where the solid blue line represents the KADK, and the solid red line represents the CHIN population. **(e)** Pairwise XP-EHH comparison between KADK and CHIN using 50Kb window. **f, g.** iHS results visualized in 50Kb window along the chromosome for KADK shown in solid blue color and CHIN shown in solid red color, respectively. **(h)** Dxy between KADK and CHIN.

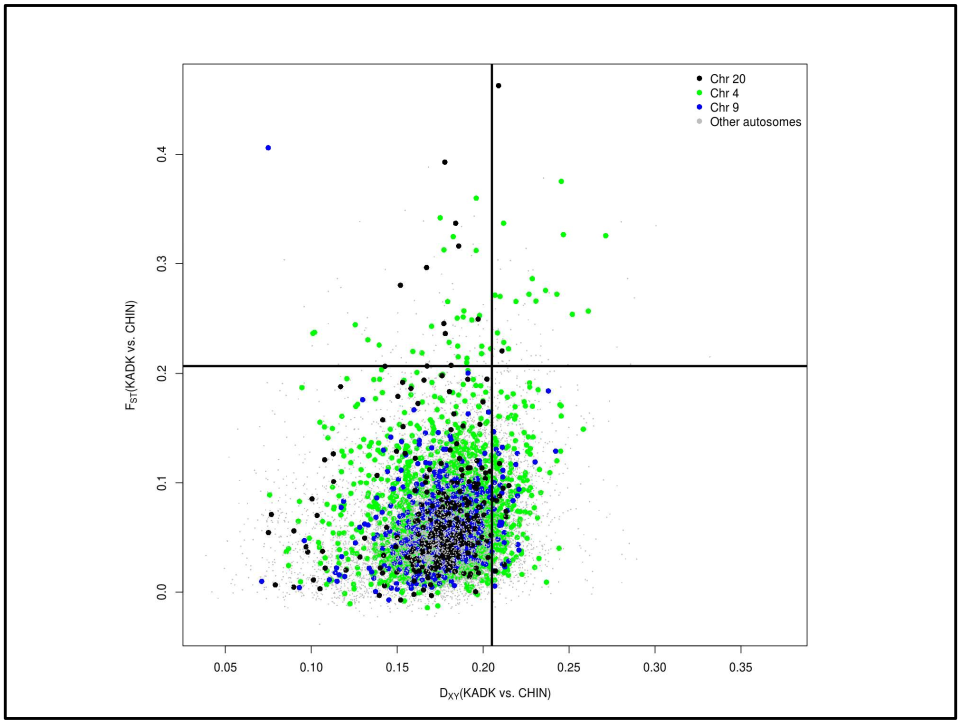

Supplementary Figure 95. F_{ST} at Y-axis and Dxy at X-axis between KADK and CHIN population has been shown. Black color dots represent Chr 20, green color dots represents Chr 4, blue color dots represents Chr 9, and grey color dots represent other autosomes. The horizontal black color solid line represents the top1% of F_{ST}, while the vertical black color solid line represents the top10% of Dxy.

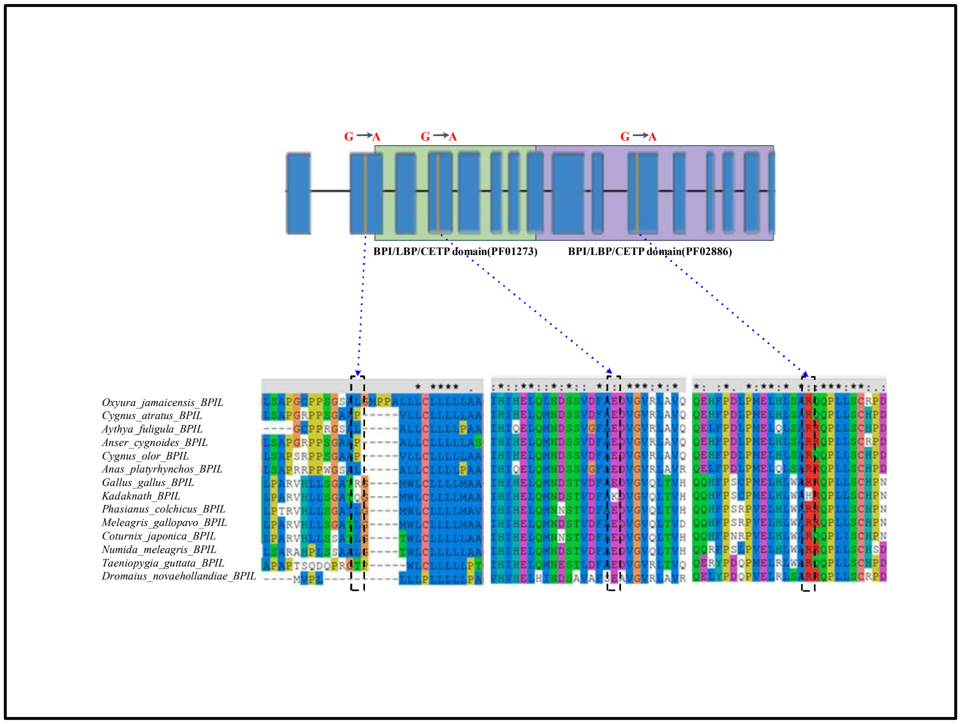

Supplementary Figure 96. Three nonsynonymous changes in *BPIL* gene. Rectangle boxes in blue color represent exons connected by a thin black line which represents introns. BPI superfamily domains are shown in light green and light purple colors. The zoomed view of amino acid change is shown by the blue color dotted arrow. The black color dotted rectangle represents the position of nonsynonymous changes**.**

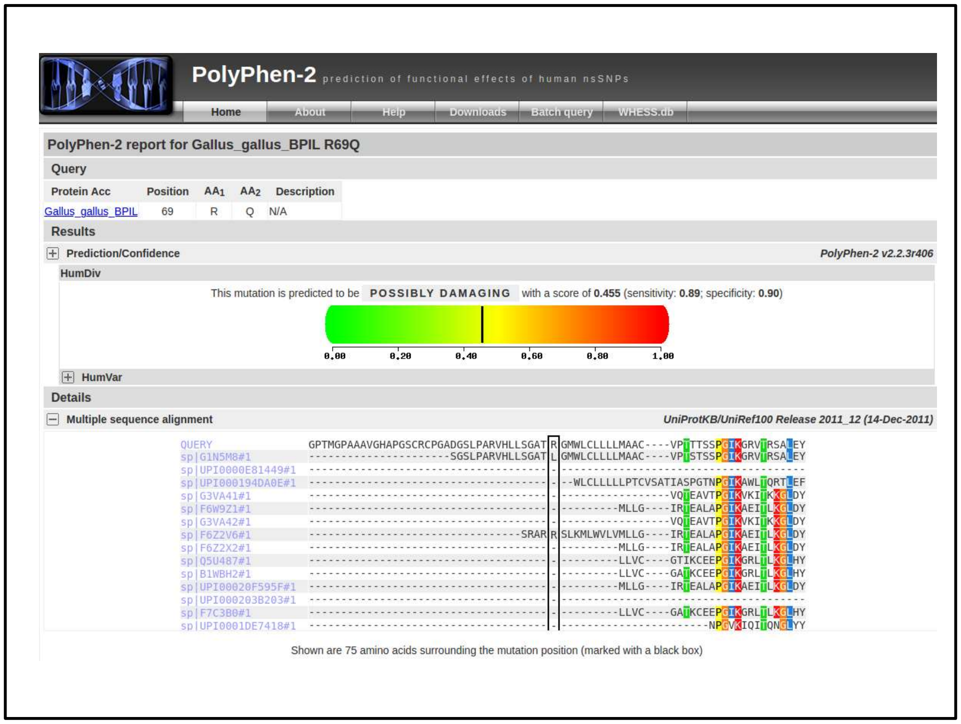

Supplementary Figure 97. Prediction of the possible impact of non-synonymous change R69Q in *BPIL* on the protein structure and function using by PolyPhen-2(Polymorphism Phenotyping v2) tool.

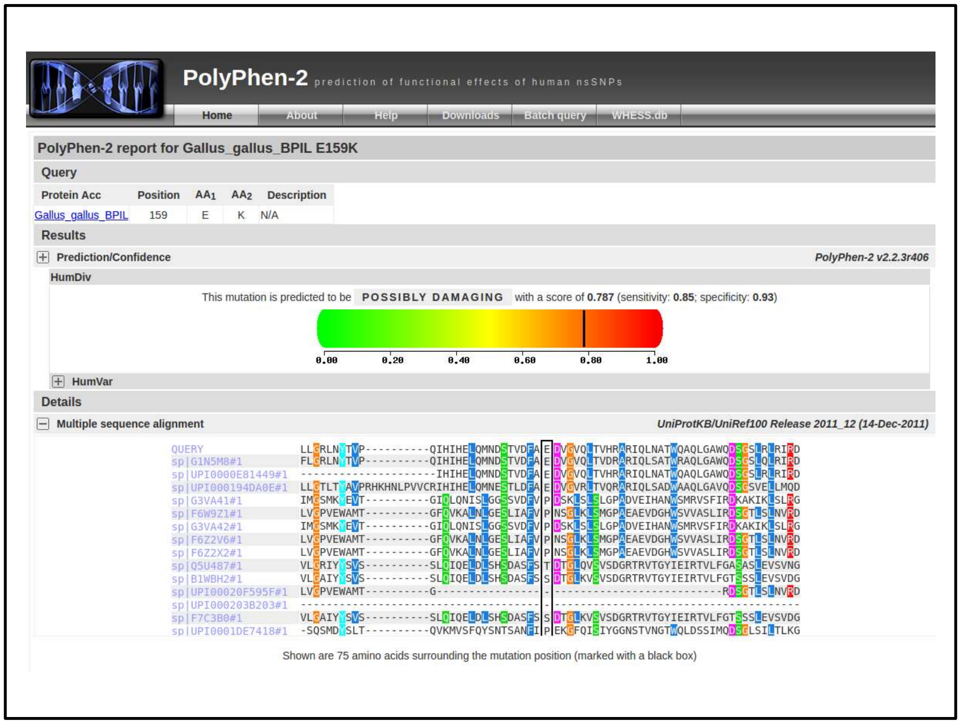

Supplementary Figure 98. Prediction of the possible impact of non-synonymous change E159K in *BPIL* on the protein structure and function using by PolyPhen-2(Polymorphism Phenotyping v2) tool.

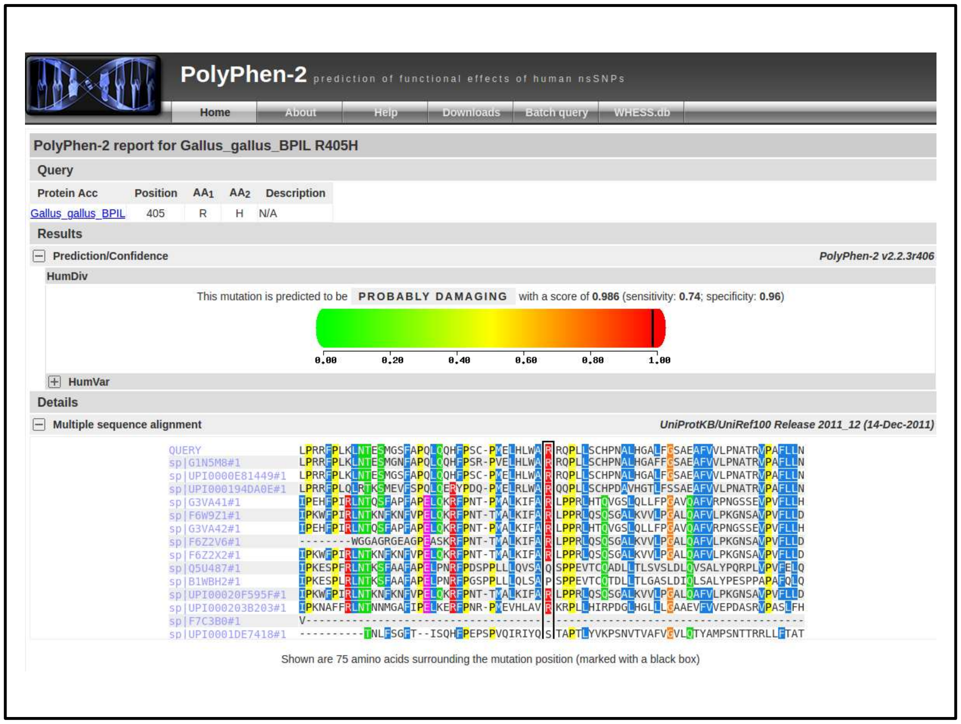

Supplementary Figure 99. Prediction of the possible impact of non-synonymous change R405H in *BPIL* on the protein structure and function using the PolyPhen-2(Polymorphism Phenotyping v2) tool.

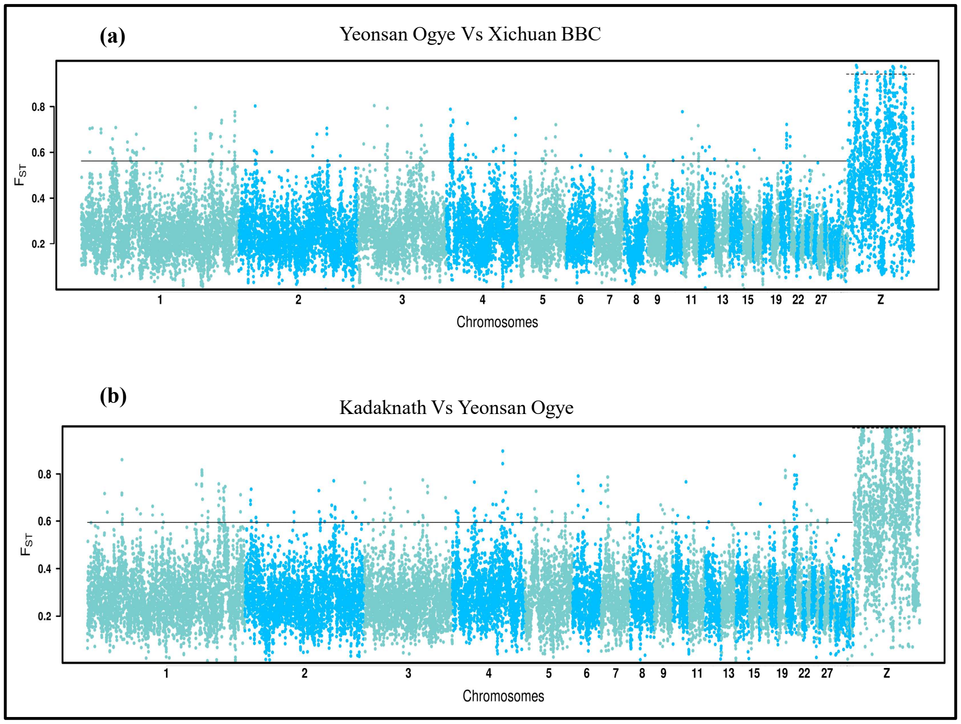

Supplementary Figure 100. Genome-wide landscape of pairwise genetic differentiation (F_{ST}) **(a)** between Yeonsan Ogye and Xichuan black-bone chicken **(b)**. between Kadaknath and Yeonsan Ogye in 50Kb non-overlapping windows. The dark slate gray and deep sky blue colors represent the alternative chromosomes. The dotted horizontal black line marks the 99th percentile outlier of estimated F_{ST} for autosome and the Z chromosome, respectively.

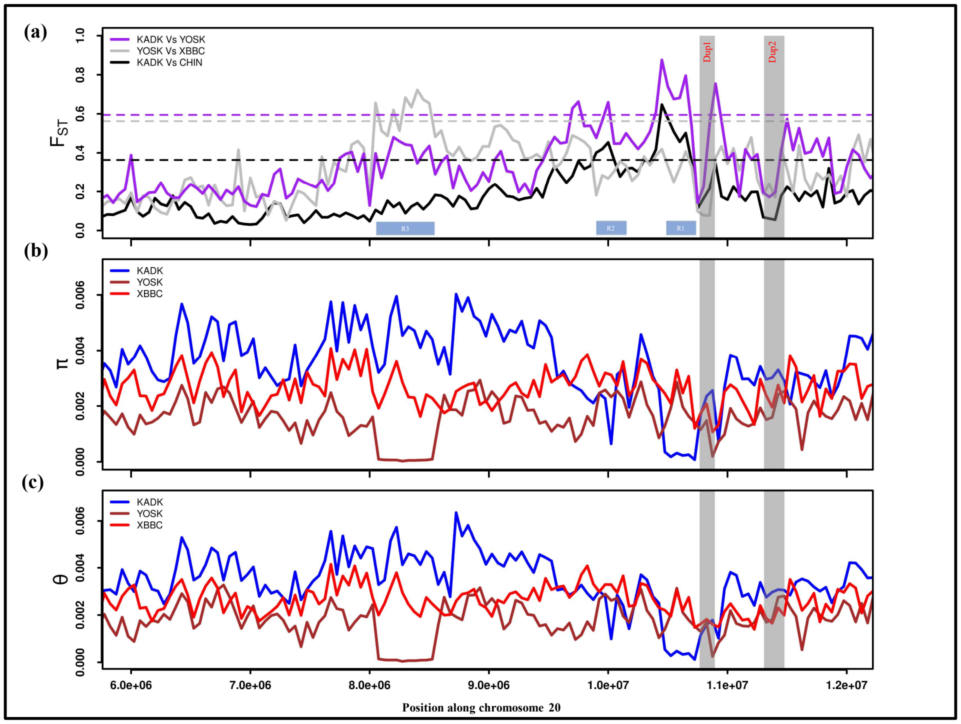

Supplementary Figure 101. (a).Pairwise F_{ST} comparisons between three population pairs along chromosome 20, where KADK vs. CHIN is in the solid black line, YOSK vs. XBBC in the solid grey line, and KADK vs. YOSK in the purple color solid line. The dotted horizontal lines of the same color represent the 99 percentile outlier for each population comparison. The Blue boxes represent the regions(R1 and R2) with high F_{ST} identities, while the vertical transparent grey color represents the Dup1 and Dup2 regions in all panes. **(b)** within population pairwise nucleotide diversity (π) and **(c)** genetic diversity. The blue line represents the KADK population, the brown represents the YOSK population, and the red represents the XBBC population.

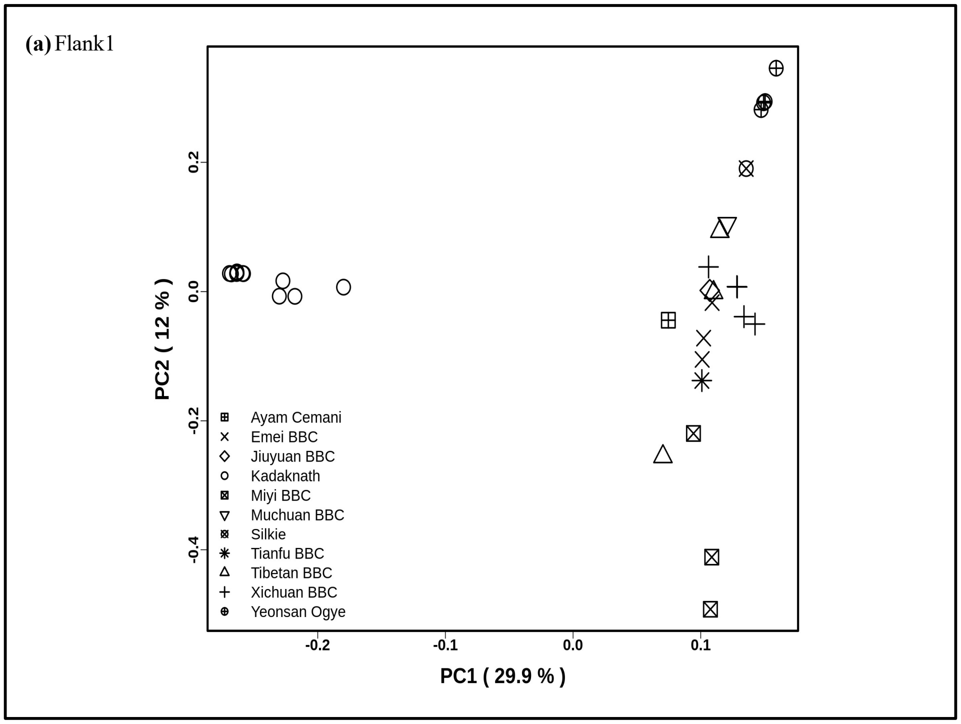

Supplementary Figure 102a. Principal component analysis (PCA) of *Fm* region for 34 black-bone chickens with the first two principal groups (PC1 and PC2) of Flank1. Each breed is shown in black color with different shapes for each breed.

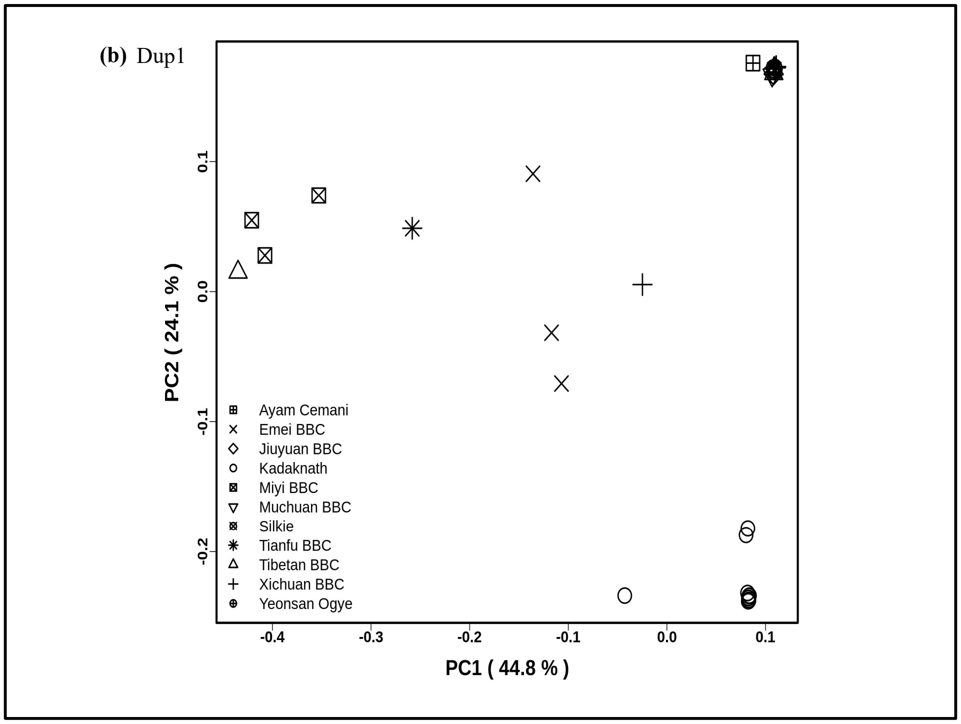

Supplementary Figure 102b. Principal component analysis (PCA) of *Fm* region for 34 black-bone chickens with the first two principal groups (PC1 and PC2) of Dup1. Each breed is shown in black color with different shapes for each breed.

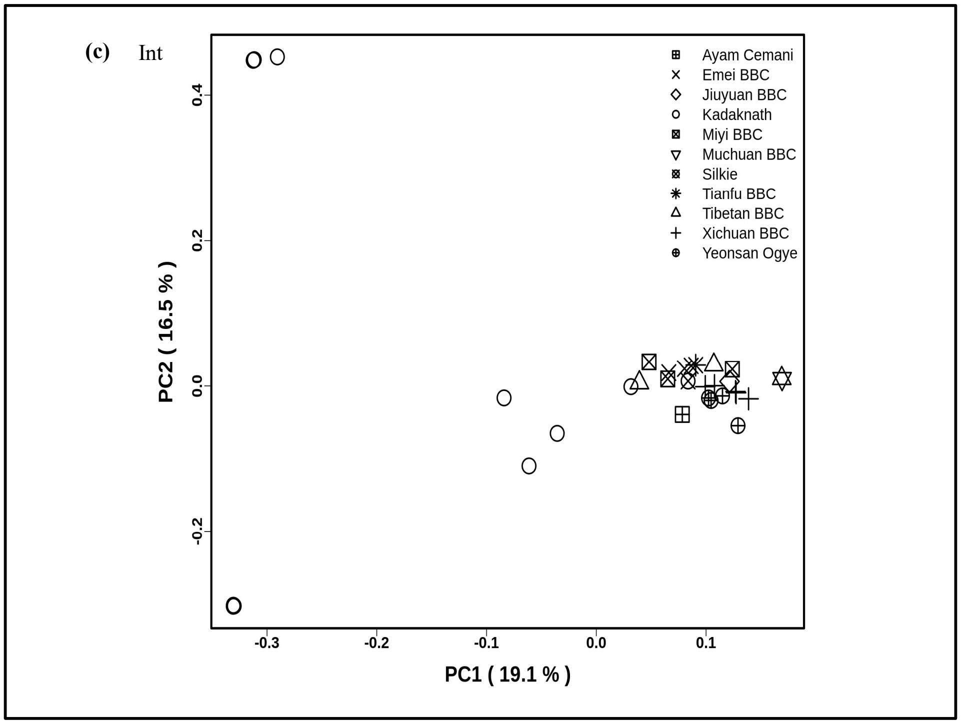

Supplementary Figure 102c. Principal component analysis (PCA) of *Fm* region for 34 black-bone chickens with the first two principal groups (PC1 and PC2) of Int. Each breed is shown in black color with different shapes for each breed.

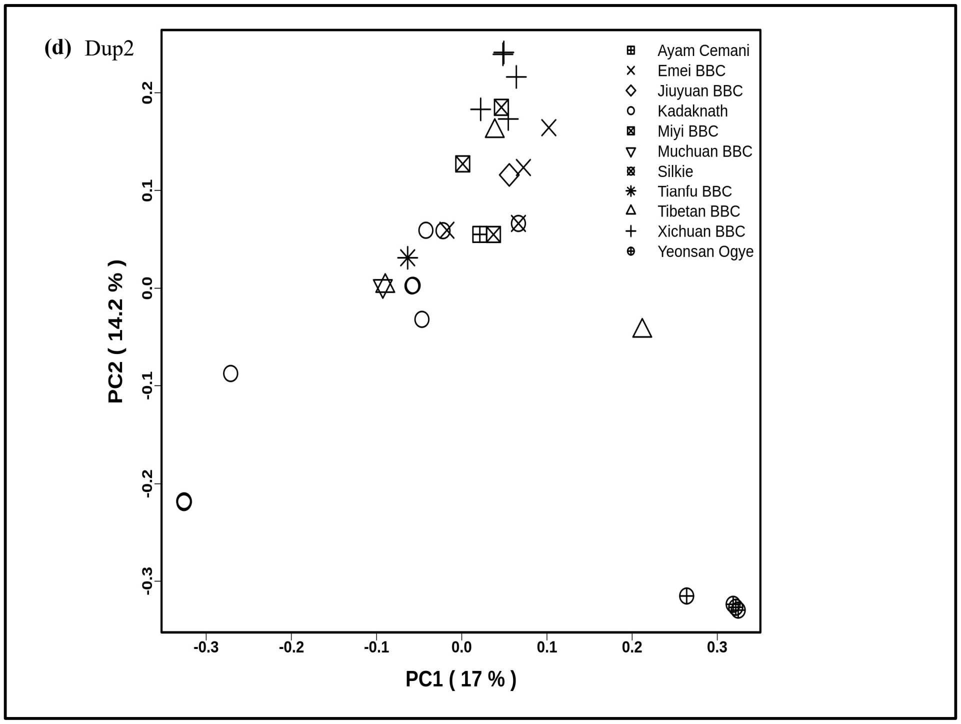

Supplementary Figure 102d. Principal component analysis (PCA) of *Fm* region for 34 black-bone chickens with the first two principal groups (PC1 and PC2) of Dup2 . Each breed is shown in black color with different shapes for each breed.

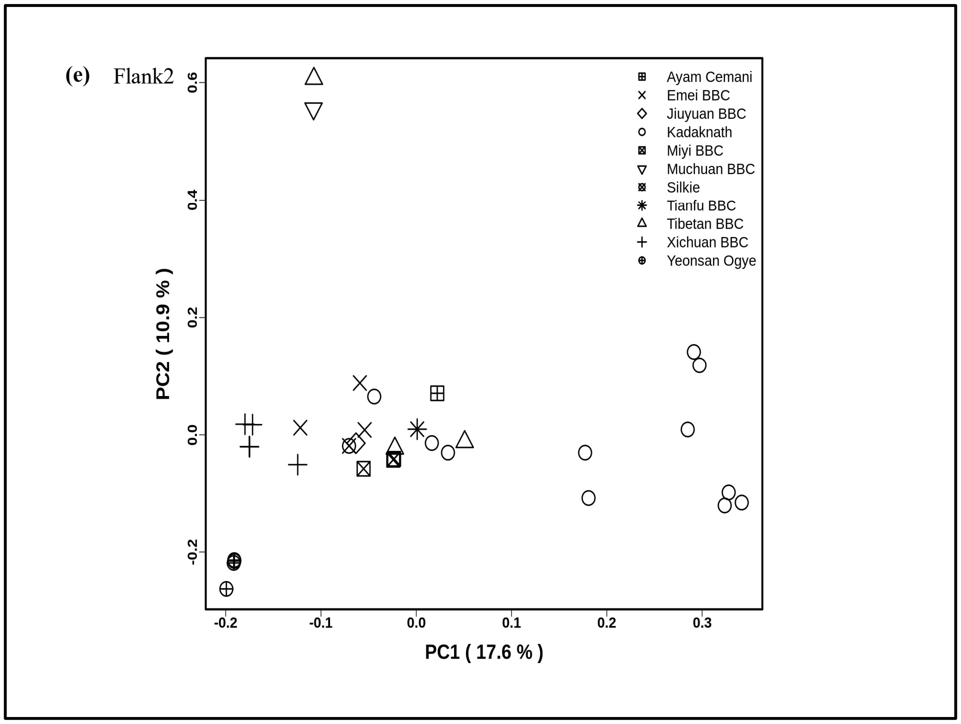

Supplementary Figure 102e. Principal component analysis (PCA) of *Fm* region for 34 black-bone chickens with the first two principal groups (PC1 and PC2) of Flank2. Each breed is shown in black color with different shapes for each breed.

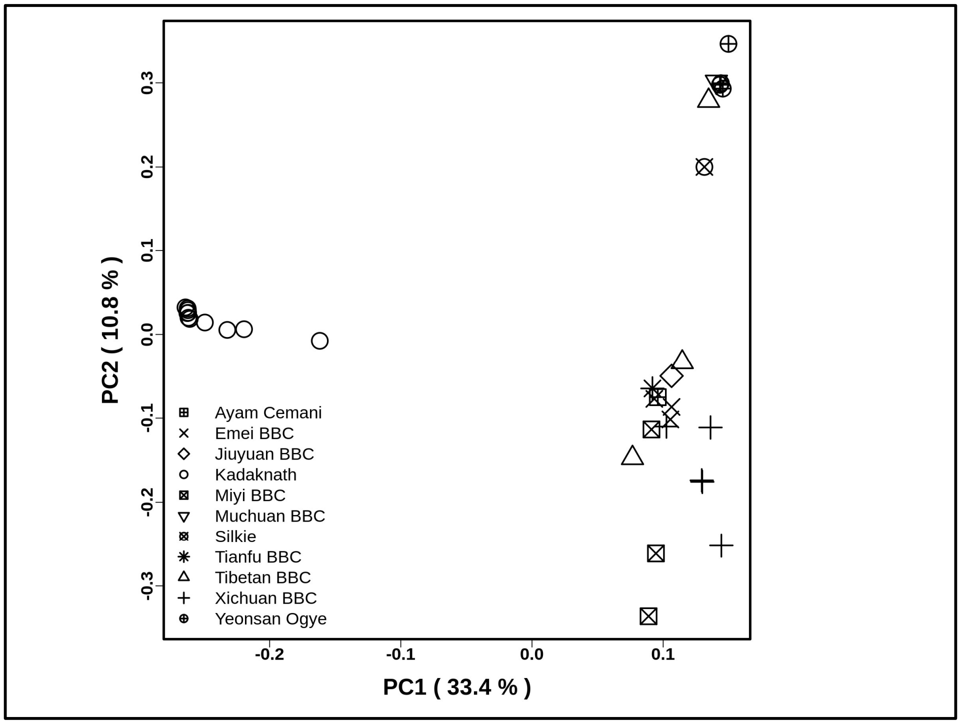

Supplementary Figure 103. Principal component analysis (PCA) of R1 region for 34 black-bone chickens with the first two principal groups (PC1 and PC2). Each breed is shown in black color with different shapes for each breed.

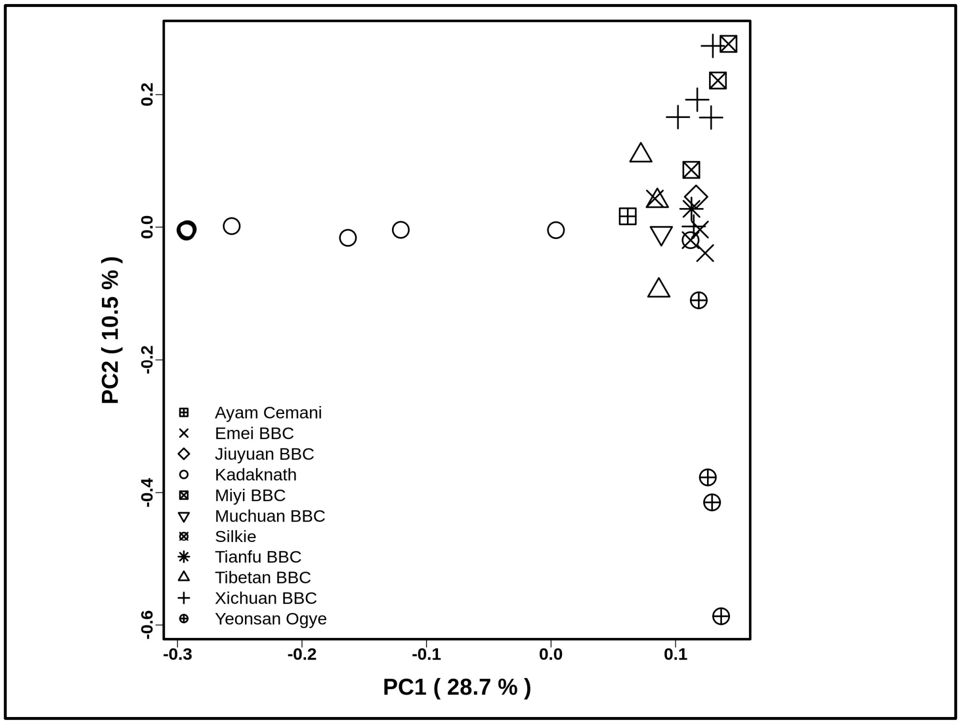

Supplementary Figure 104. Principal component analysis (PCA) of R2 region for 34 black-bone chickens with the first two principal groups (PC1 and PC2). Each breed is shown in black color with different shapes for each breed.

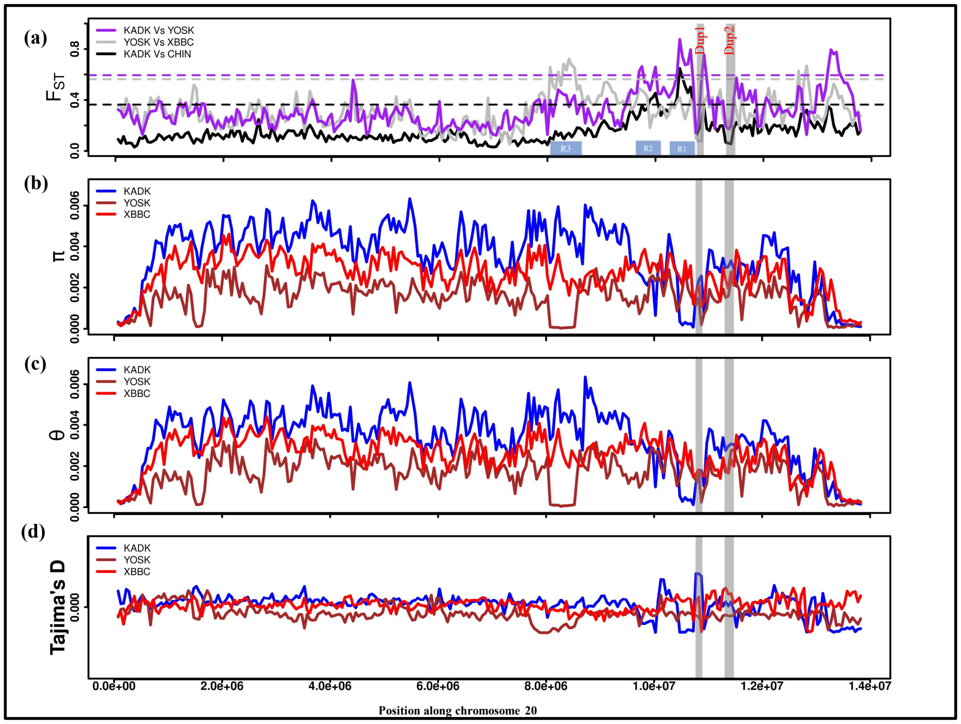

Supplementary Figure 105. (a).Pairwise F_{ST} comparisons between three population pairs along complete chromosome 20, where KADK vs. CHIN is in the solid black line, YOSK vs. XBBC in the solid grey line, and KADK vs. YOSK in the purple color solid line. The dotted horizontal lines of the same color represent the 99 percentile outlier for each population comparison. The Blue boxes represent the regions (R1, R2, R3) with high F_{ST} identities, while the vertical transparent grey color represents the Dup1 and Dup2 regions in all panes. **(b)** within population pairwise nucleotide diversity (π),**(c)** genetic diversity and **(d)** Tajima's D . The blue line represents the KADK population, the brown represents the YOSK population, and the red represents the XBBC population.
