# Supplementary material for: Decoding the fibromelanosis locus complex chromosomal rearrangement of black-bone chicken: genetic differentiation, selective sweeps and protein-coding changes in Kadaknath chicken
Source: Front Genet. 2023 Jun 22;14:1180658. doi: 10.3389/fgene.2023.1180658 (PMC10325862; doi:10.3389/fgene.2023.1180658)
Supplement: Supplementary file 2 [file DataSheet1.pdf]

Decoding the Fibromelanosis Locus Complex Chromosomal Rearrangement of Black-Bone Chicken: Genetic differentiation, selective sweeps and protein-coding changes in Kadaknath Chicken

Sagar Sharad Shinde[1], Ashutosh Sharma[1], Nagarjun Vijay[1]

[1]Computational Evolutionary Genomics Lab, Department of Biological Sciences, IISER Bhopal, Bhauri, Madhya Pradesh, India

*Correspondence: nagarjun@iiserb.ac.in

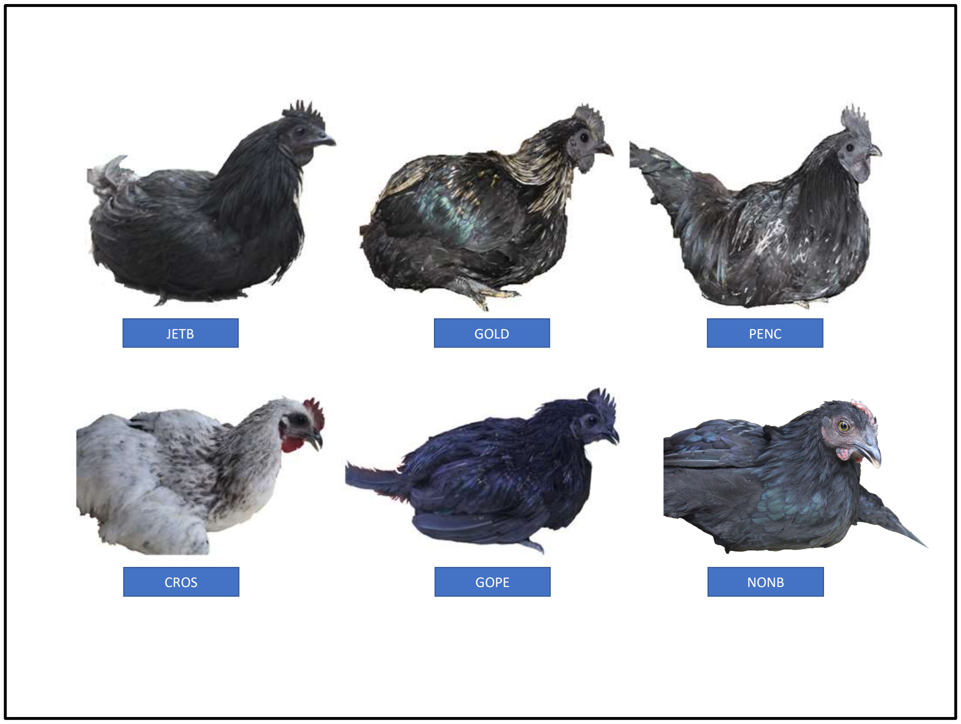

Supplementary Figure 1. The first row has photographs of the Kadaknath morphs Jetblack (JETB), Golden (GOLD), and Pencil (PENC). The second row has photographs of CROS, which has black internal organs while feathers are white with a red comb, Golden-Pencil-like phenotype (GOPE) and non-black individual (NONB) which have internal organs are non-black while the feathers are black with red comb color.

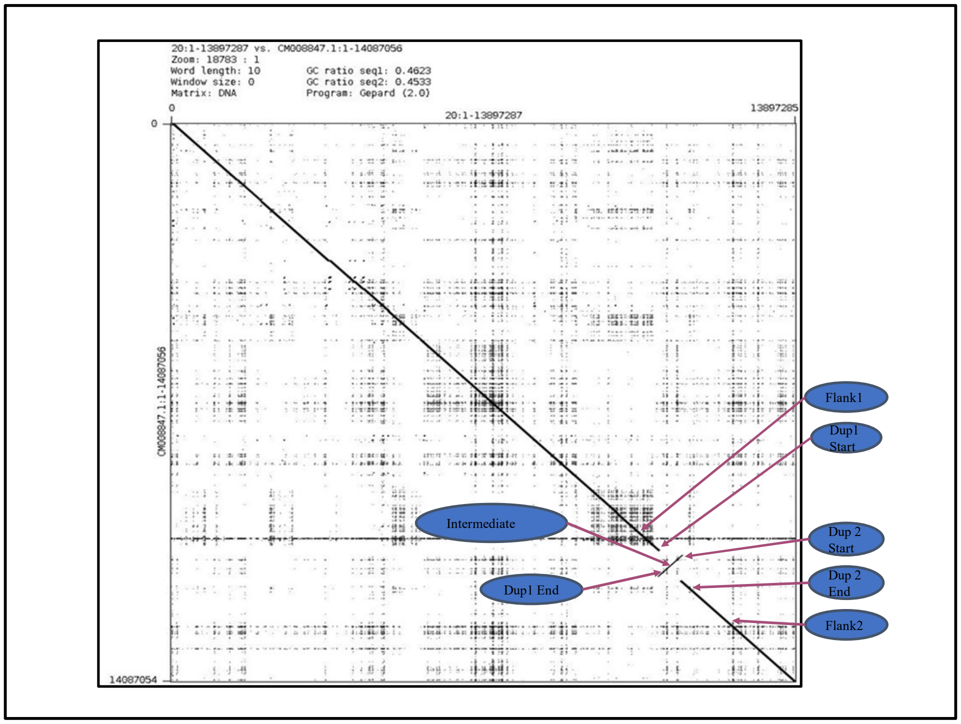

Supplementary Figure 2. Dot plot between chromosome 20 of chicken genome assembly (Gallus_gallus.GRCg6a) with chromosome 20 (CM008847.1) Yeonsan ogye genome. The figure was generated using Gepard2.1. At *Fm* locus region in the dot plot the rearranged region can be seen.

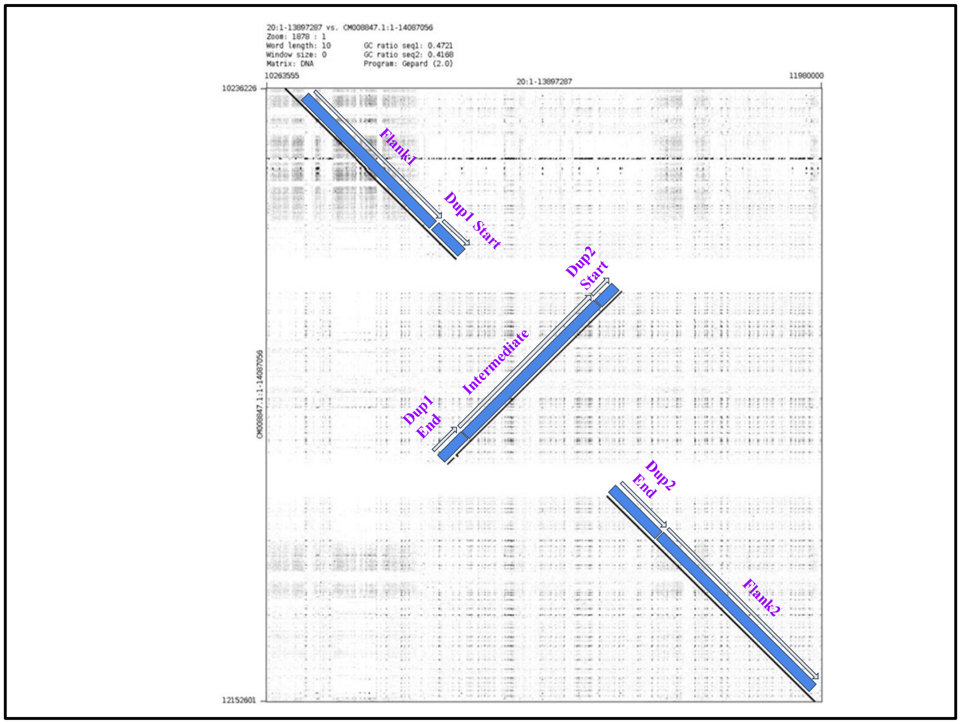

Supplementary Figure 3. Zoomed view of dot plot between chromosome 20 chicken genome assembly (Gallus_gallus.GRCg6a) with chromosome 20 (CM008847.1) Yeonsan ogye genome. The figure was generated using Gepard2.1. Zoomed view of dot plot at *Fm* region.

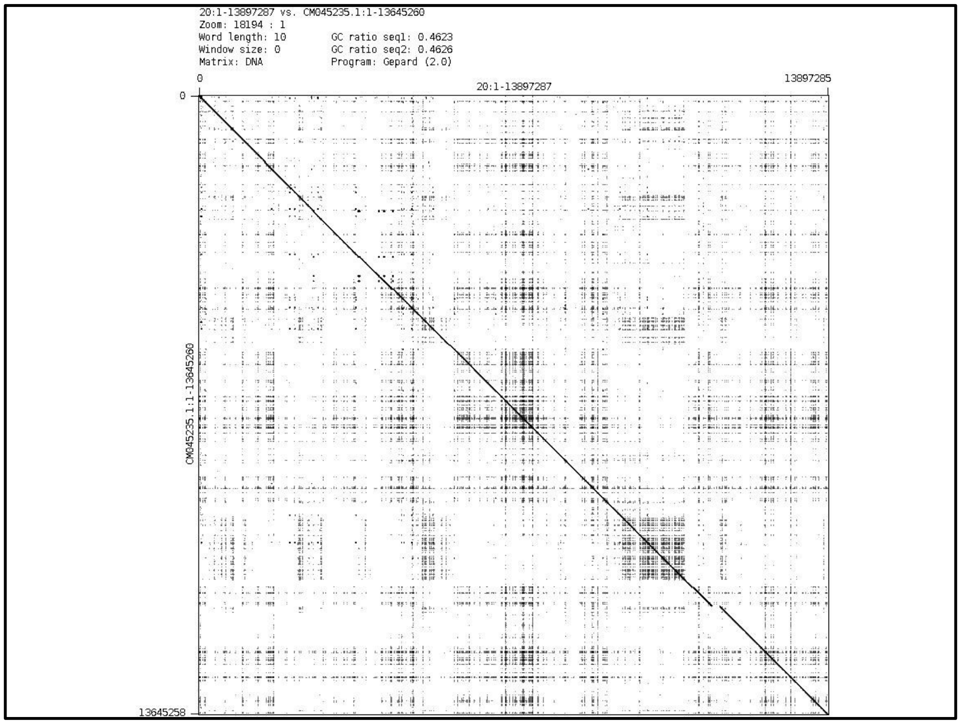

Supplementary Figure 4. Dot plot between chromosome 20 of chicken genome assembly (Gallus_gallus.GRCg6a) with chromosome 20 (CM045235.1) of silkie3 genome assembly (GCA_024653025.1). The figure was generated using Gepard2.1.

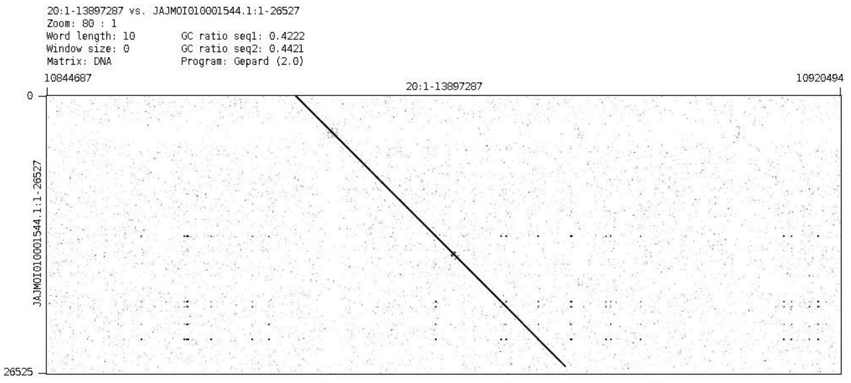

Supplementary Figure 5. Dot plot between chromosome 20 of chicken genome assembly (Gallus_gallus.GRCg6a) with unplaced scaffold JAJMOI010001544.1 of silkie3 genome assembly (GCA_024653025.1). The figure was generated using Gepard2.1.

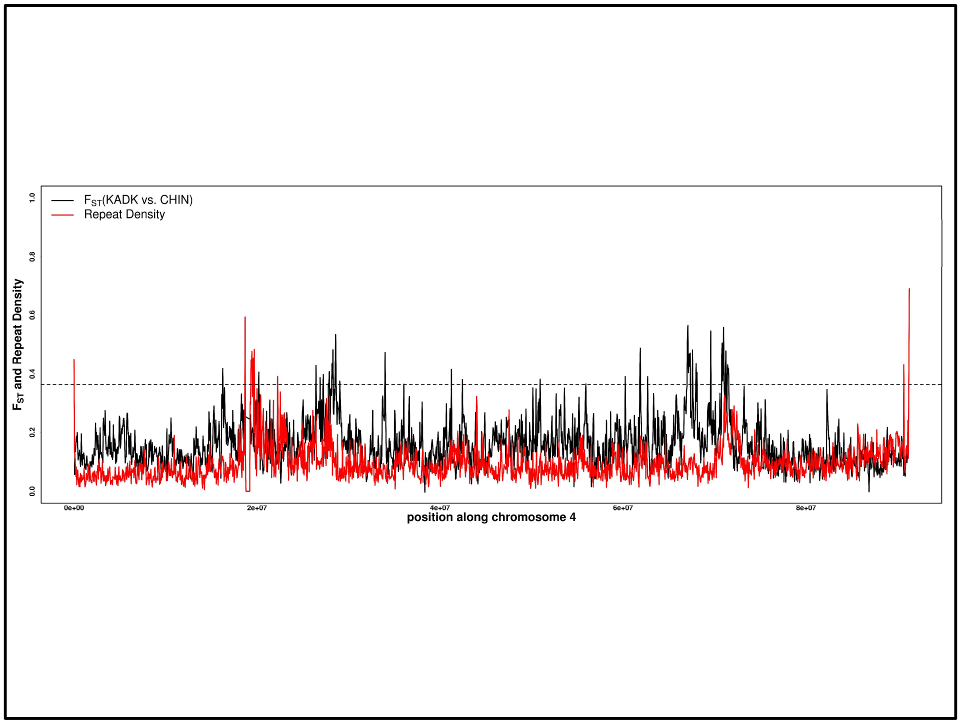

Supplementary Figure 6. Repeat density and F_{ST} along with chromosome 4 are shown. A horizontal black dotted line represents the 99 percentile F_{ST} threshold. The solid red line represents repeat density in Galgal6 genome assembly calculated in 50Kb windows, and the solid black line represents a pairwise F_{ST} comparison between KADK and CHIN population using 50Kb windows.

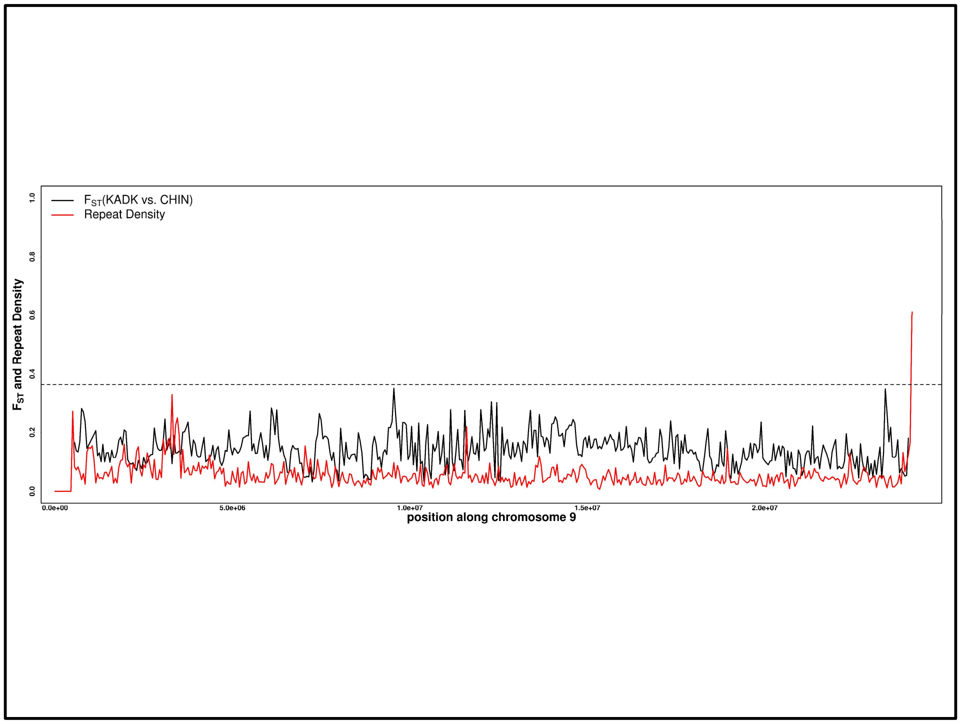

Supplementary Figure 7. Repeat density and F_{ST} along with chromosome 9 are shown. A horizontal black dotted line represents the 99 percentile F_{ST} threshold. The solid red line represents repeat density in Galgal6 genome assembly calculated in 50Kb windows, and the solid black line represents a pairwise F_{ST} comparison between KADK and CHIN population using 50Kb windows.

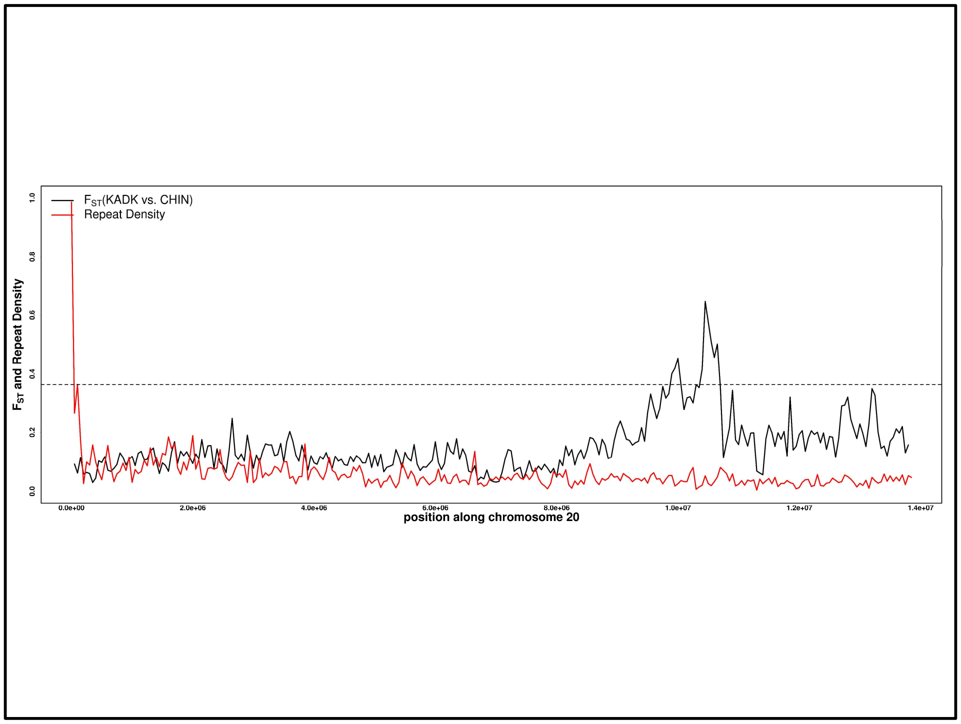

Supplementary Figure 8. Repeat density and F_{ST} along with chromosome 20 are shown. A horizontal black dotted line represents the 99 percentile F_{ST} threshold. The solid red line represents repeat density in Galgal6 genome assembly calculated in 50Kb windows, and the solid black line represents a pairwise F_{ST} comparison between KADK and CHIN population using 50Kb windows.

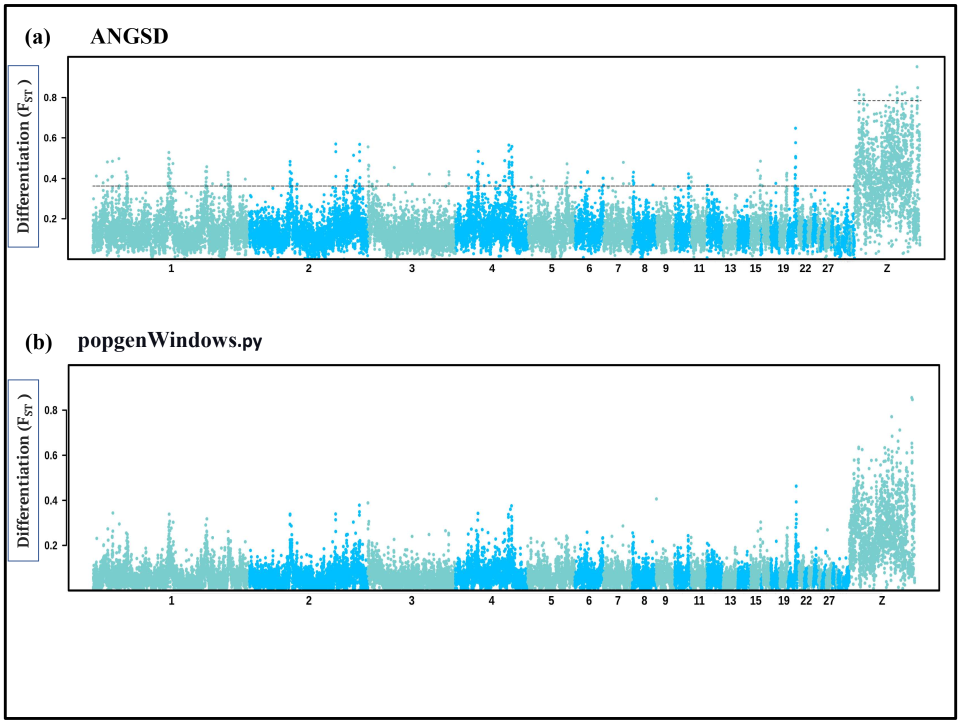

Supplementary Figure 9. Pairwise F_{ST} between KADK and CHIN population calculated using **(a)** ANGSD **(b)** popgenWindows.py. The dark slate gray and deep sky blue colors represent the alternative chromosomes, dotted horizontal black line marks the 99th percentile outlier F_{ST} estimated for autosome and Z chromosome.

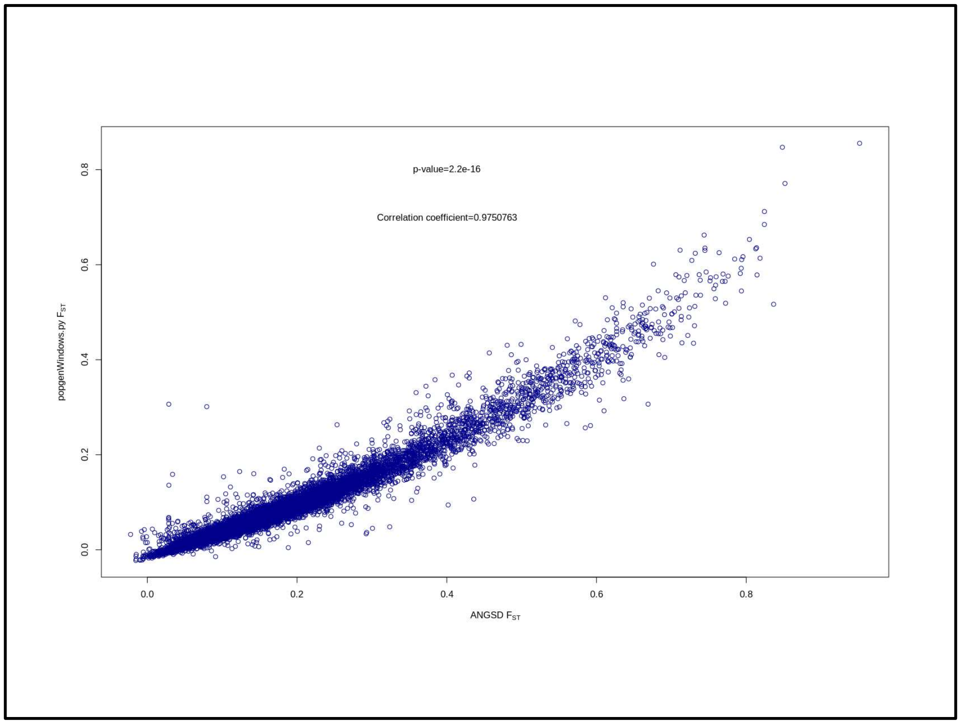

Supplementary Figure 10. Pearson correlation coefficient test on the F_{ST} comparison from ANGSD and popgenWindows.py method. F_{ST} from popgenWindows.py at Y-axis and F_{ST} from ANGSD at X-axis has a significant positive correlation with a correlation coefficient= 0.975 shown in blue circles.

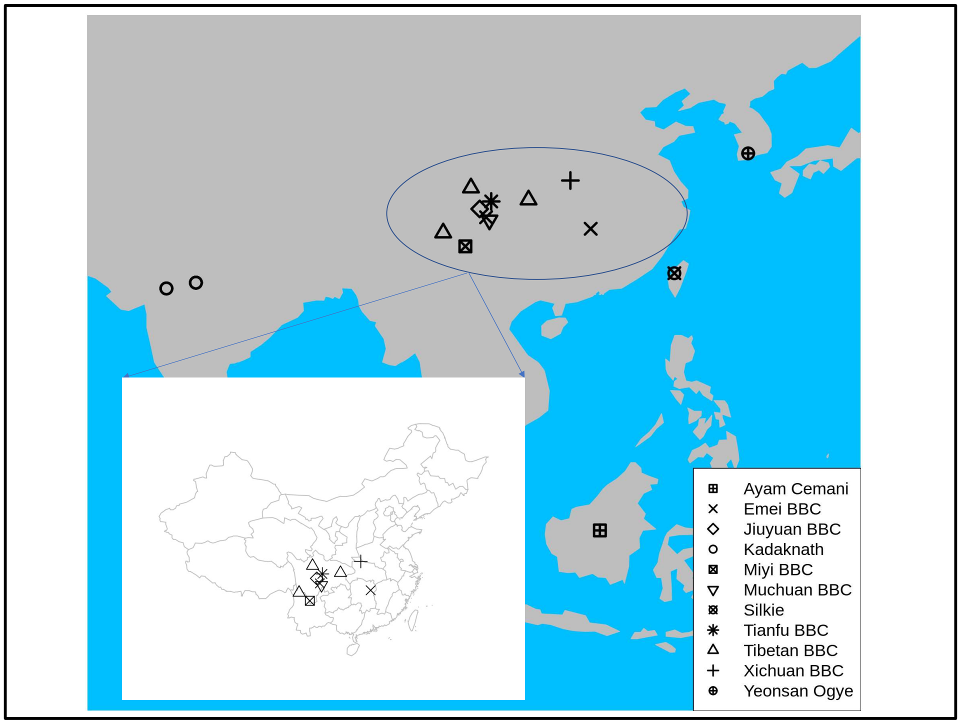

Supplementary Figure 11. Geographical locations of black-bone chicken breeds used in this study. The map was generated using rworldmap, map, and mapdata packages implemented in R. Different shapes represent different breeds. All breeds in oval shape circles belong to China, and the zoomed view of the China map represents the sample location of each breed.

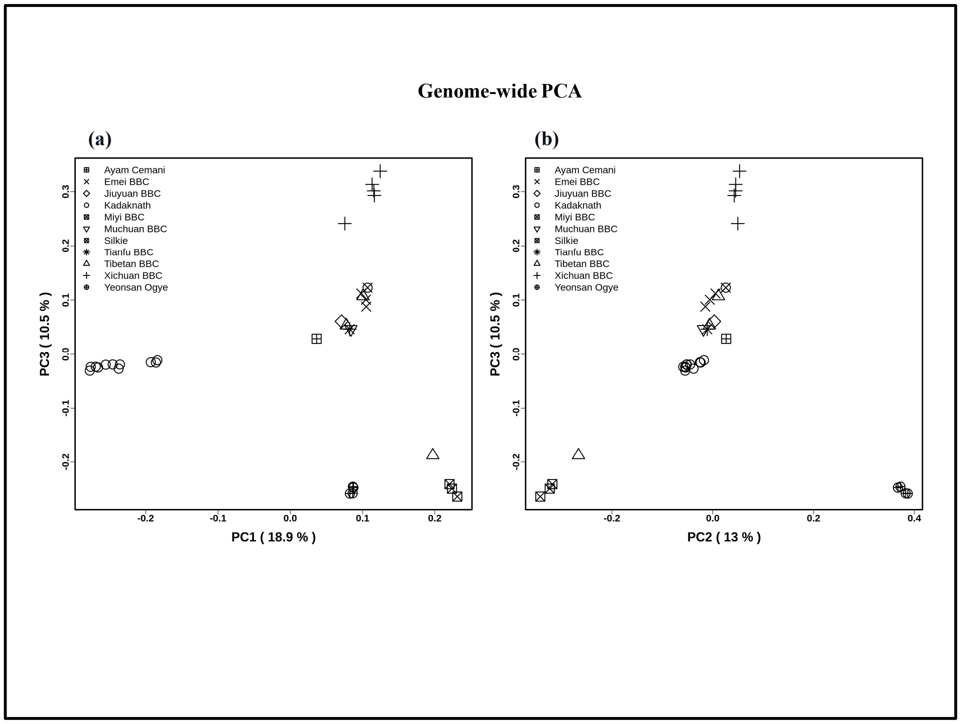

Supplementary Figure 12. Genome-wide principal component analysis (PCA) 34 black-bone chickens with the two principal groups **(a)** PC1 and PC3 and **(b)** PC2 and PC3 . Each breed is shown in black color with different shapes for each breed.

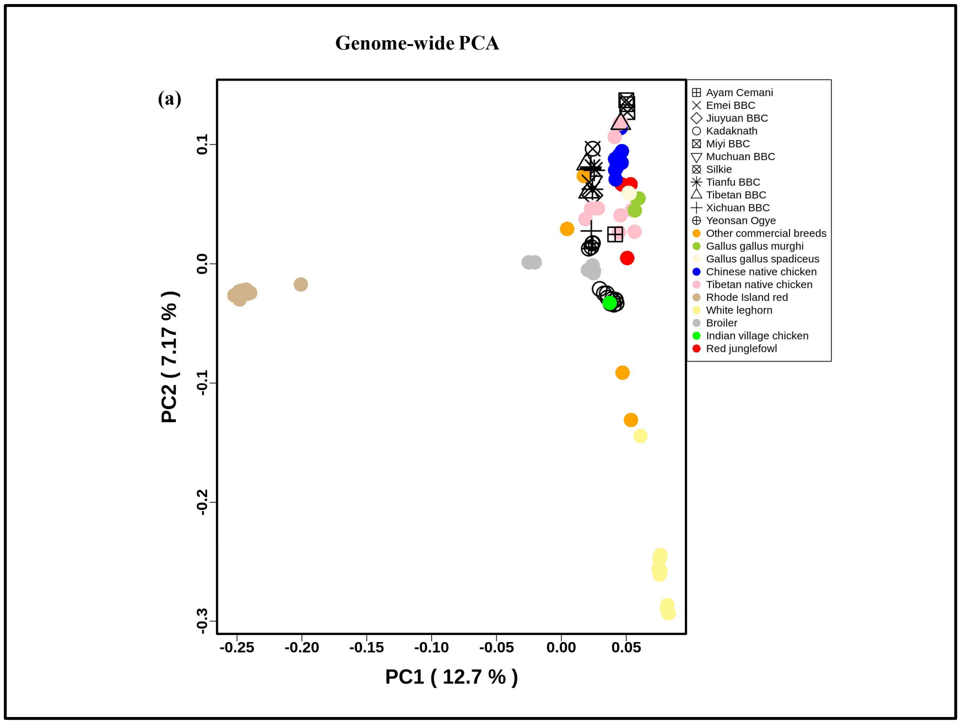

Supplementary Figure 13a. Genome-wide PCA plot generated for 101 chicken individuals: PC1 and PC2 explained 12.7% and 7.17 % variance, respectively. Non-black-bone breeds are shown by different colors. All black-bone breeds are shown in black color with different shapes for each breed.

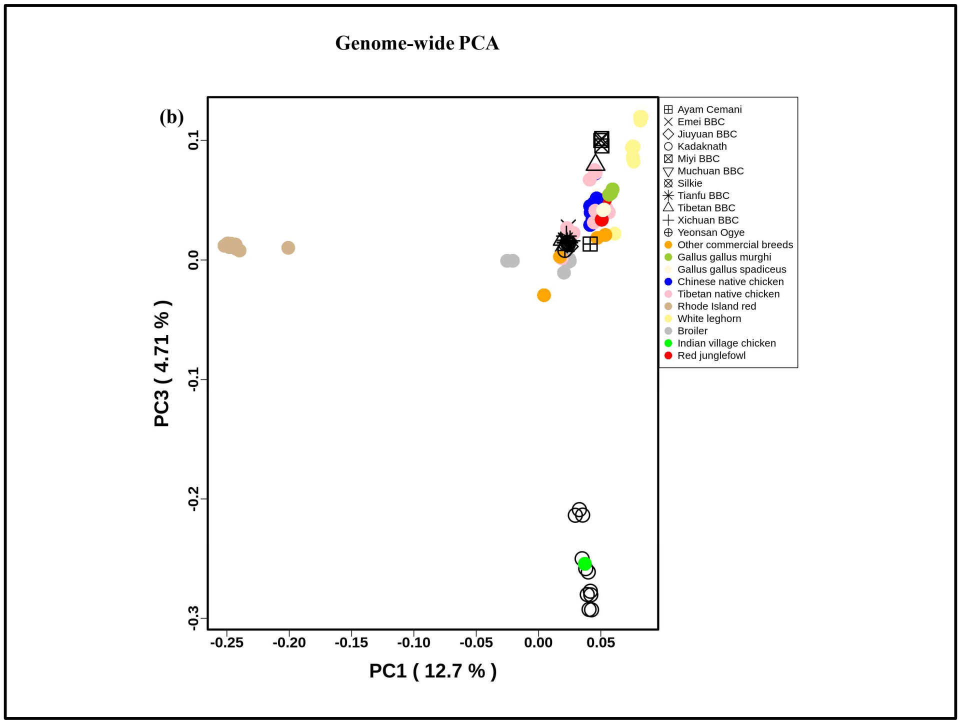

Supplementary Figure 13b. Genome-wide PCA plot generated for 101 chicken individuals: PC1 and PC3 explained 12.7% and 4.71% variance, respectively. Non-black-bone breeds are shown by different colors. All black-bone breeds are shown in black color with different shapes for each breed.

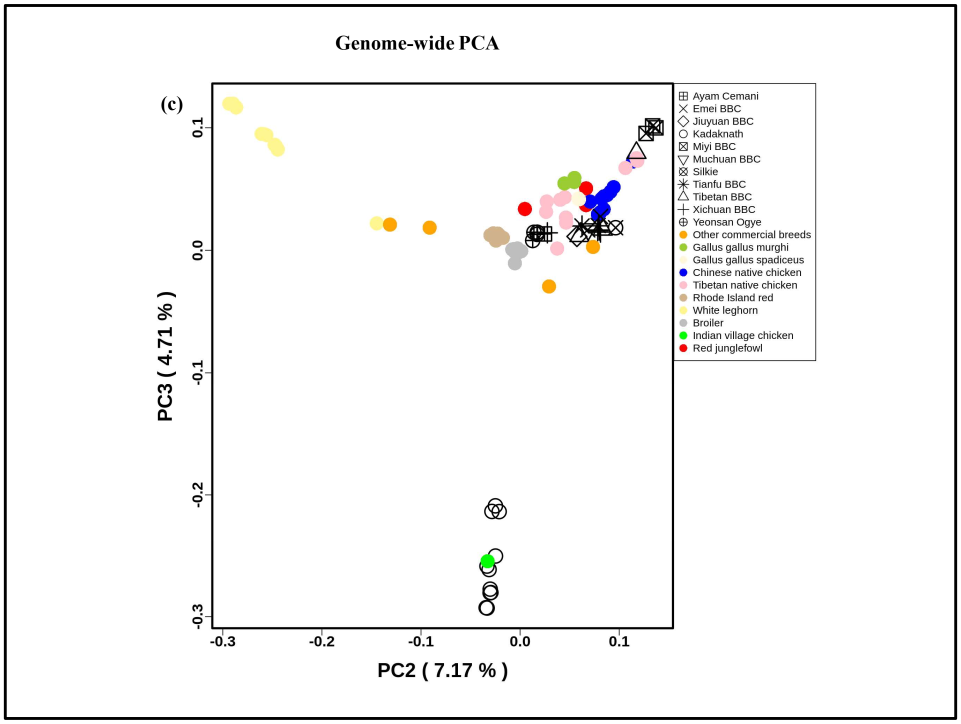

Supplementary Figure 13c. Genome-wide PCA plot generated for 101 chicken individuals: PC2 and PC3 explained 7.17% and 4.71% variance, respectively. Non-black-bone breeds are shown by different colors. All black-bone breeds are shown in black color with different shapes for each breed.

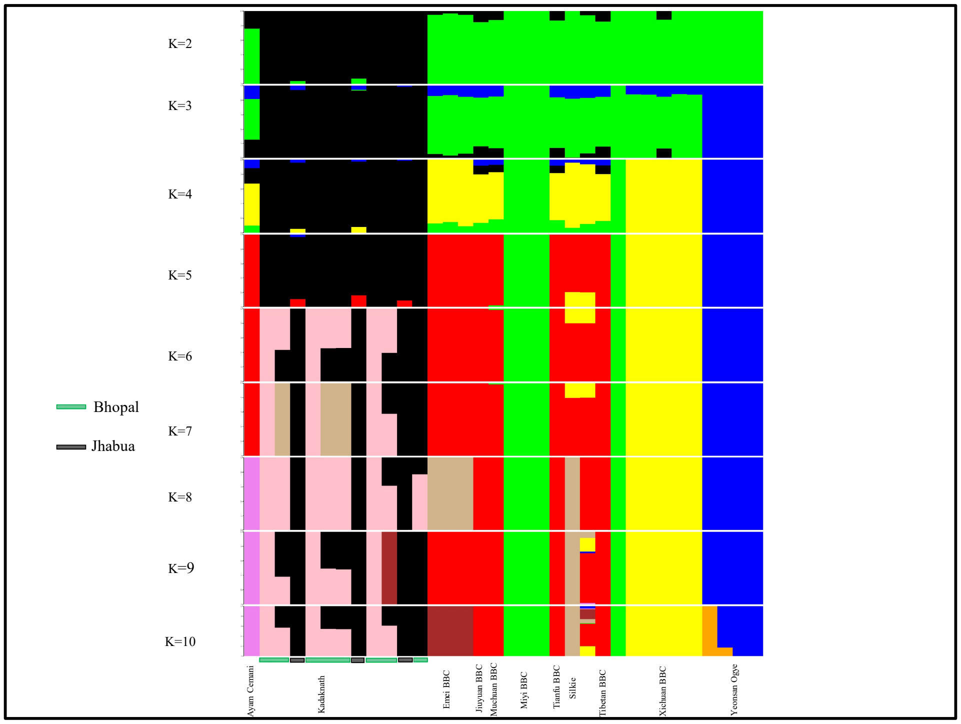

Supplementary Figure 14a. Genome-wide admixture plot of 34 black-bone individuals using NGSadmix from K=2 to K=10. Within the Kadaknath, two sub-clusters (Bhopal and Jhabua) are shown in green and black rectangle boxes, respectively.

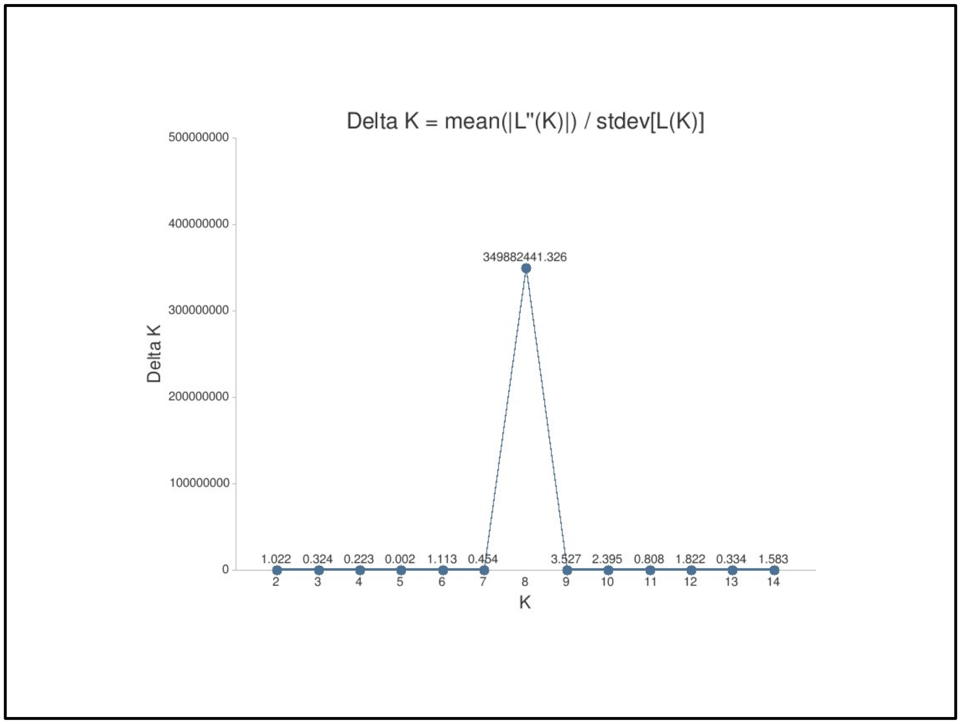

Supplementary Figure 14b. Delta K value plot for the admixture analysis of 34 BBC individuals generated by CLUMPAK web server.

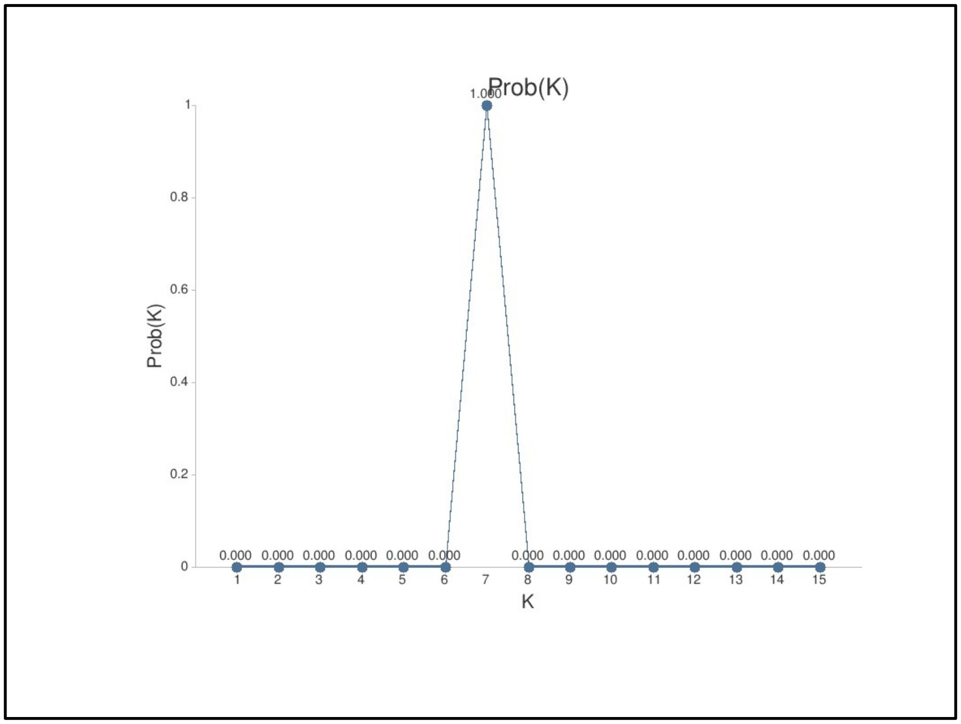

Supplementary Figure 14c. Log probability of the data referred to as ln Pr(X|K) value plot for the admixture analysis of 101 individuals generated by CLUMPAK web server.

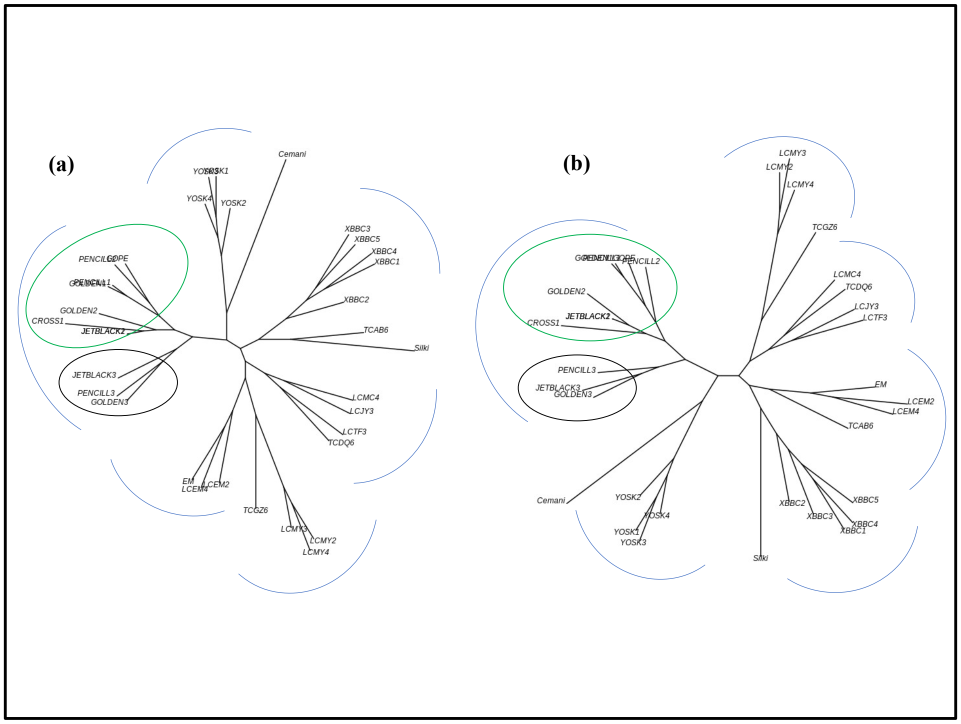

Supplementary Figure 15. Genome-wide phylogeny of 34 back-bone chicken inferred using the maximum likelihood based SNPhylo method. **(a)** From the bcftools SNP data **(b)** From the freebayes SNP data. Blue curve-shaped lines represent the respective breed individuals. The black color oval shape circle represents KADK individuals from Jhabua, while the green color oval shape circle represents KADK individuals from Bhopal.

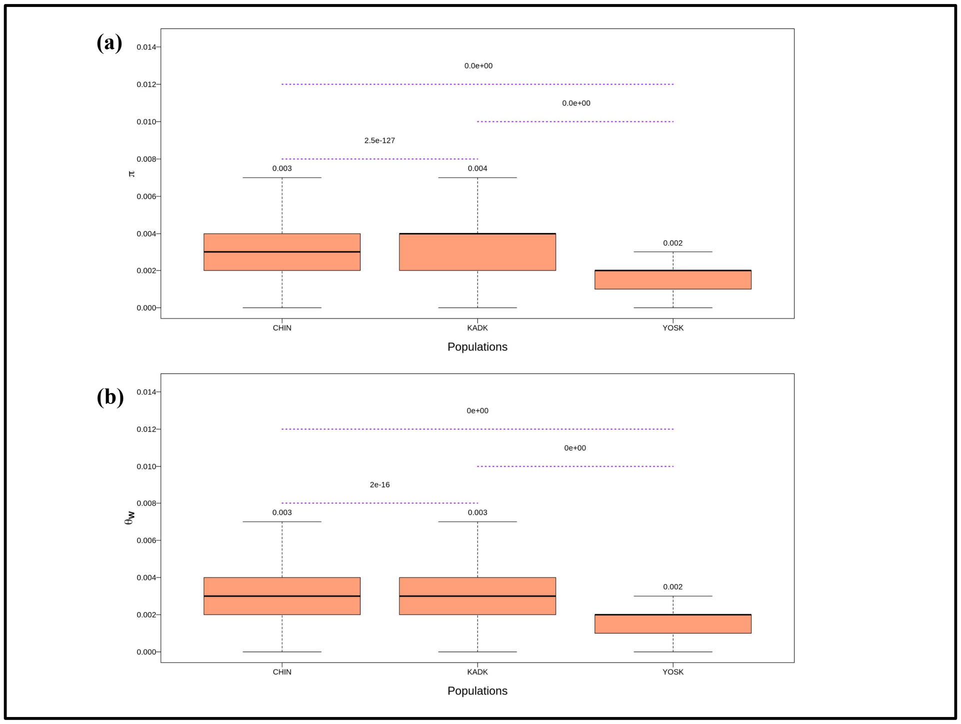

Supplementary Figure 16. (a) Nucleotide diversity (π) and **(b)** genetic diversity (θ) for three populations (CHIN, KADK, YOSK) shown using a Light salmon color boxplot with the mean of each population represented at the top of the box plot. We calculated the pairwise two-tailed Wilcox test representing the purple dotted lines between pairs and the significant p-values mentioned on the top of the dotted line.

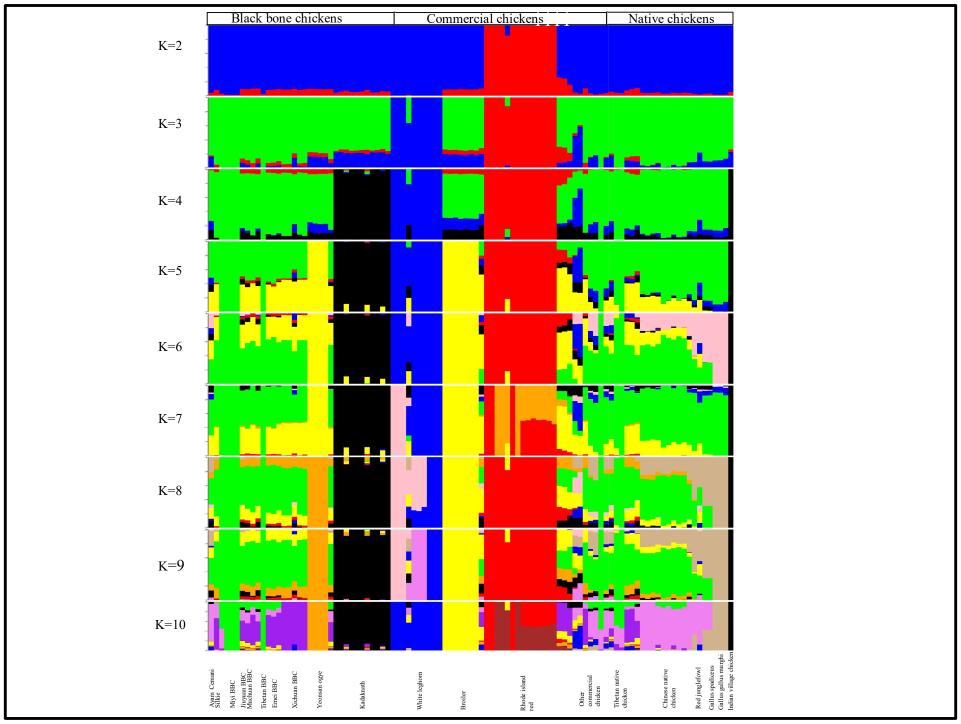

Supplementary Figure 17a. Genome-wide population genetic structure and individual ancestry were estimated using NGSadmix for 101 chickens individuals of different breeds from K=2 to K=10.

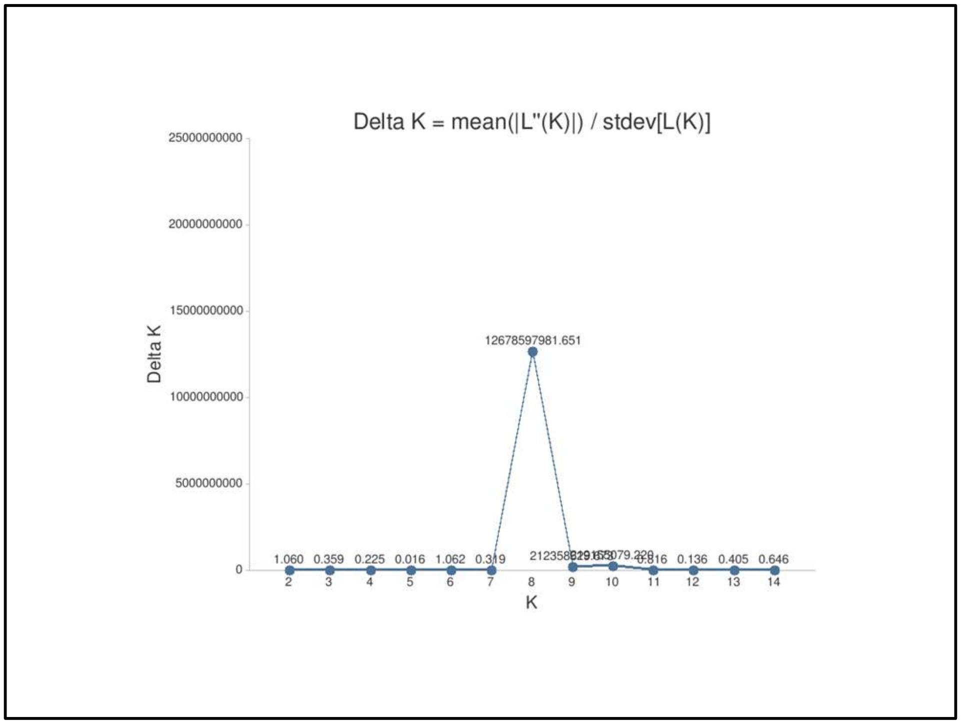

Supplementary Figure 17b. Delta K value plot for the admixture analysis of 101 individuals generated by CLUMPAK web server.

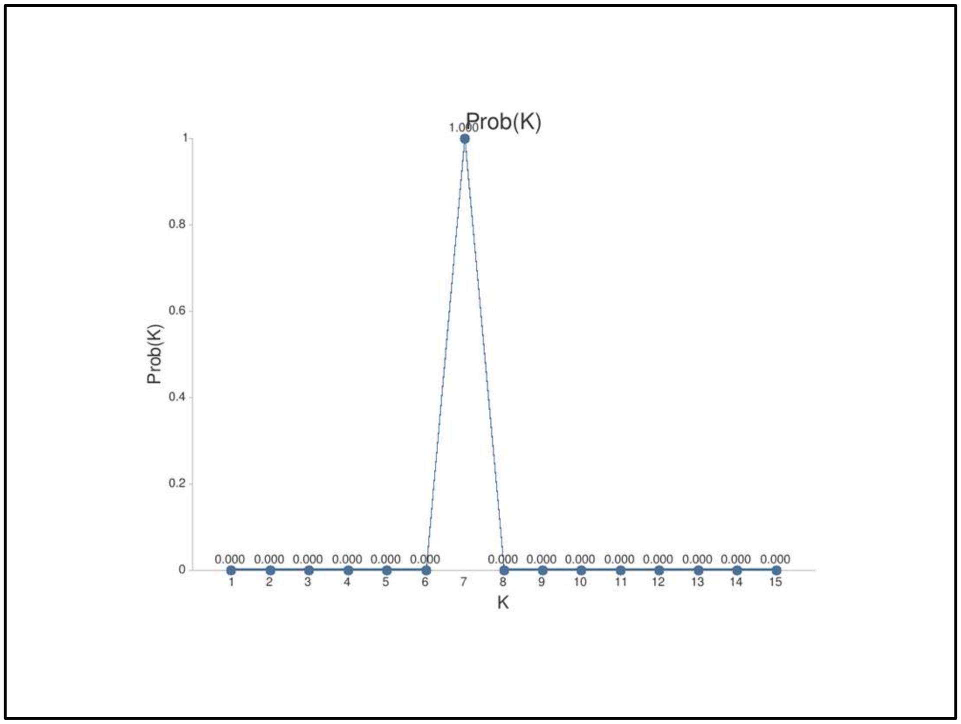

Supplementary Figure 17c. Log probability of the data referred to as ln Pr(X|K)) value plot for the admixture analysis results of 101 individuals generated by CLUMPAK web server.

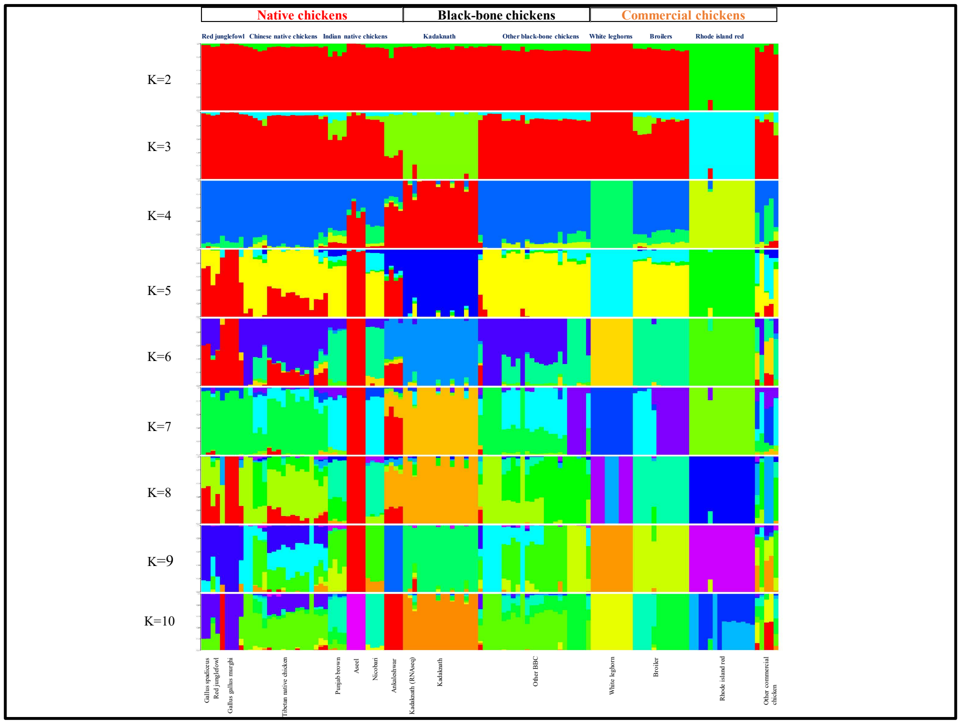

Supplementary Figure 18a. Population genetic structure and individual ancestry of 123 chicken individuals from different breeds were estimated using AdmixPipe v3.

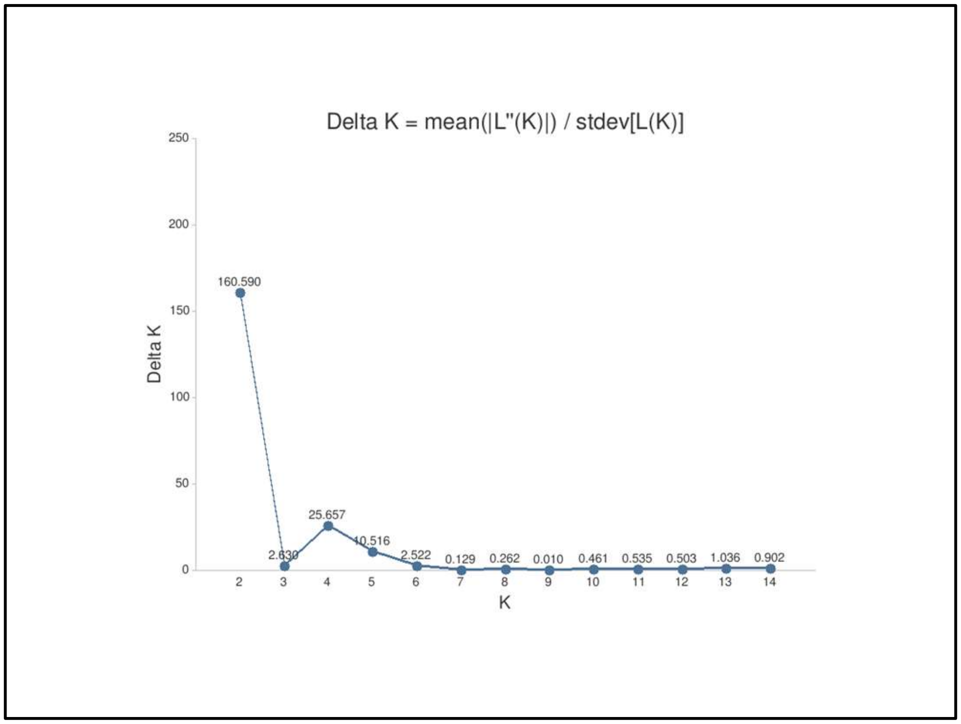

Supplementary Figure 18b. Delta K value plot for the admixture analysis of 123 individuals generated by CLUMPAK web server.

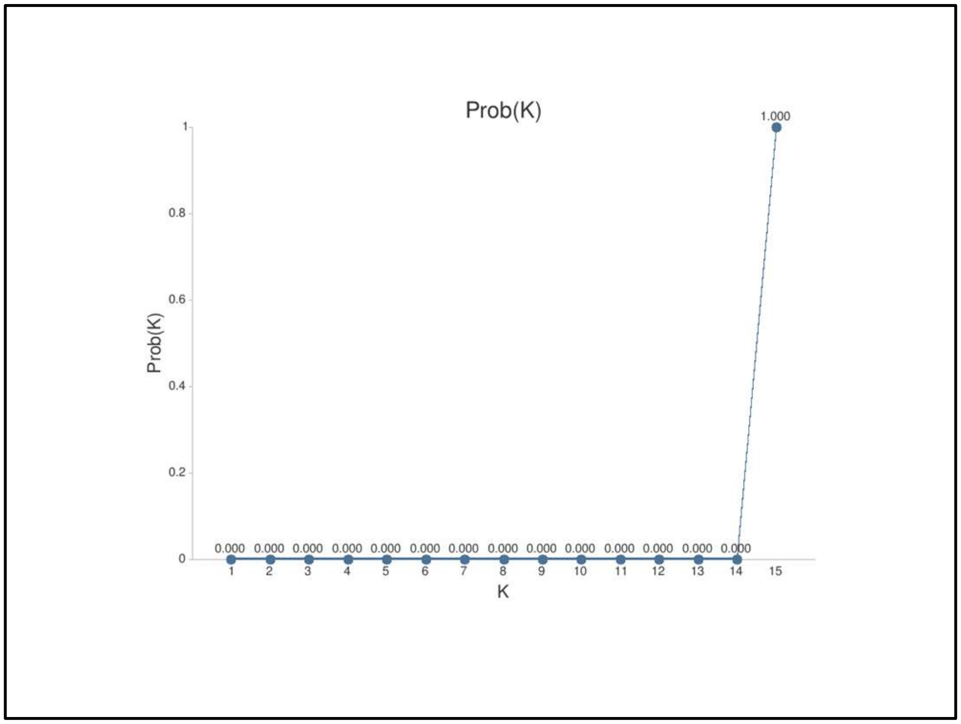

Supplementary Figure 18c. Log probability of the data referred to as ln Pr(X|K) value plot for the admixture analysis results of 123 individuals generated by CLUMPAK web server.

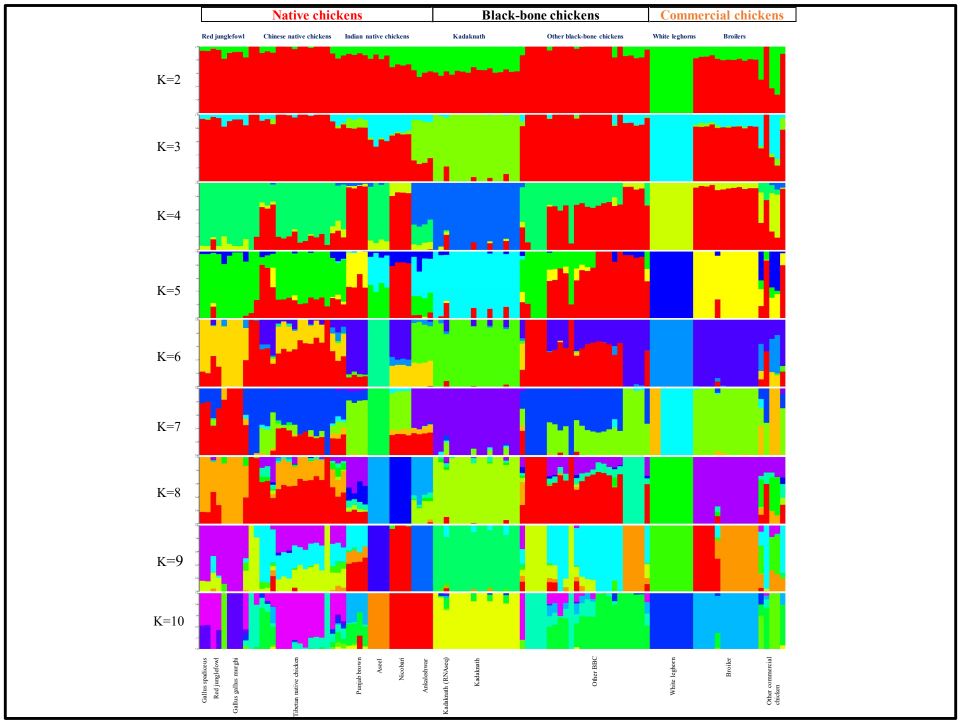

Supplementary Figure 19a. Population genetic structure and individual ancestry of 109 chicken individuals (after removing the 14 individuals of Rhode Island Red) from different breeds were estimated using AdmixPipe v3.

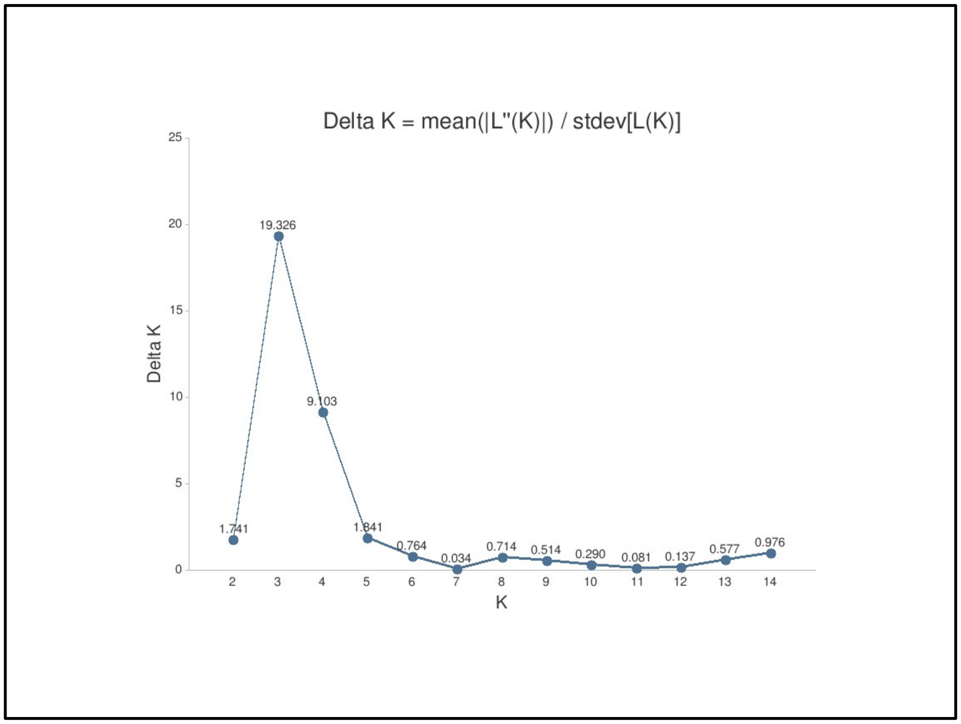

Supplementary Figure 19b. Delta K value plot for the admixture analysis of 109 individuals generated by CLUMPAK web server.

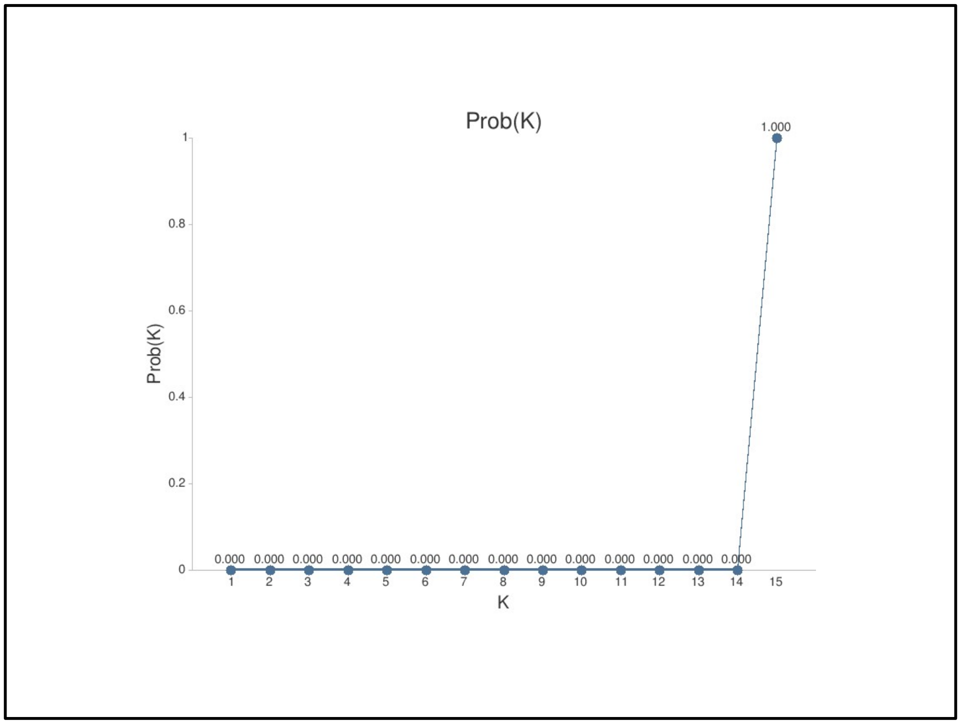

Supplementary Figure 19c. Log probability of the data referred to as ln Pr(X|K) value plot for the admixture analysis results of 109 individuals generated by CLUMPAK web server.

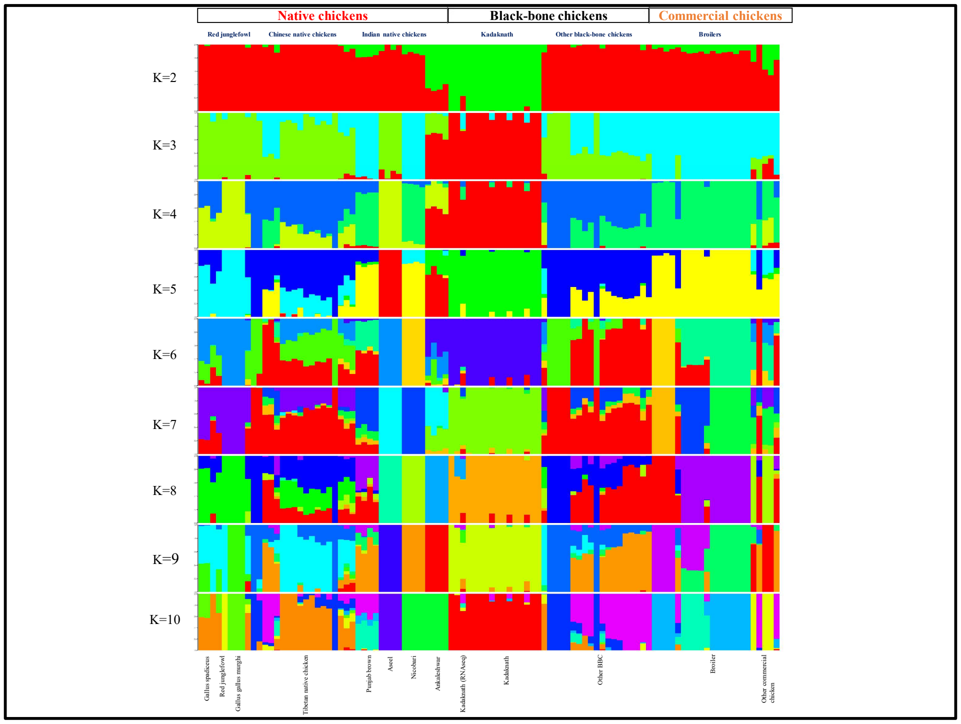

Supplementary Figure 20a. Population genetic structure and individual ancestry of 100 chicken individuals (after removing the 14 individuals of Rhode Island Red and 9a individuals of white leghorn) from different breeds were estimated using AdmixPipe v3.

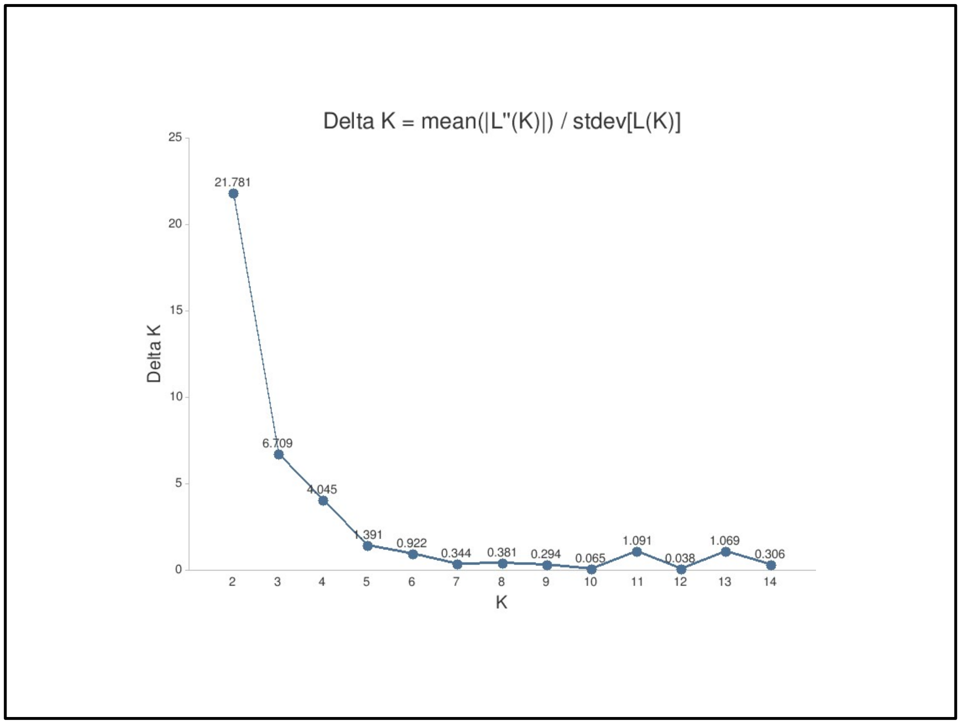

Supplementary Figure 20b. Delta K value plot for the admixture analysis of 100 individuals generated by CLUMPAK web server.

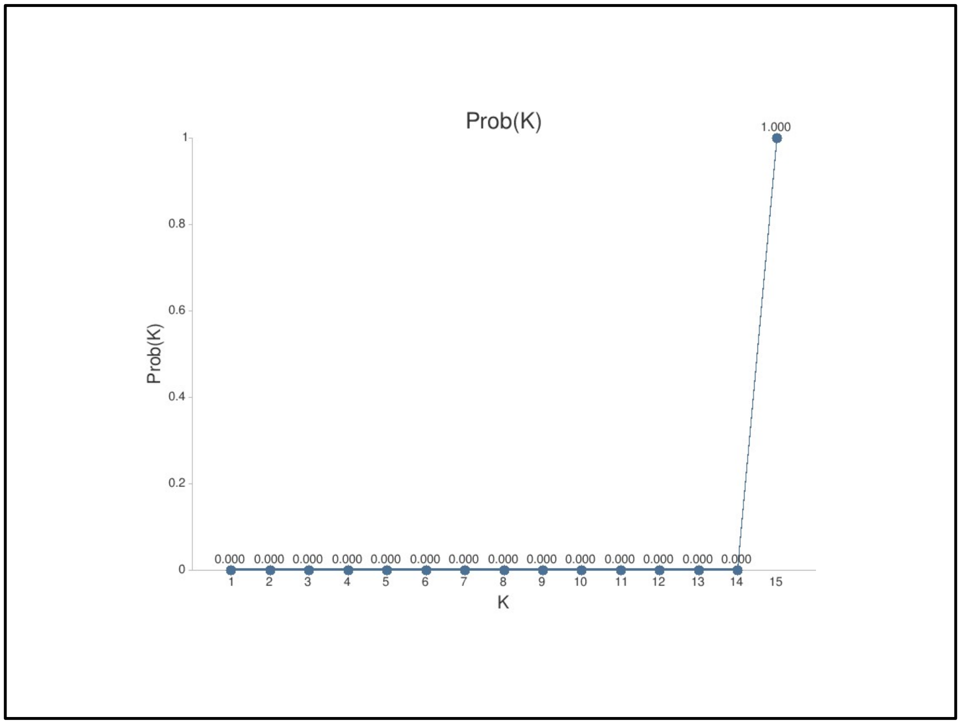

Supplementary Figure 20c. Log probability of the data referred to as ln Pr(X|K) value plot for the admixture analysis results of 100 individuals generated by CLUMPAK web server.

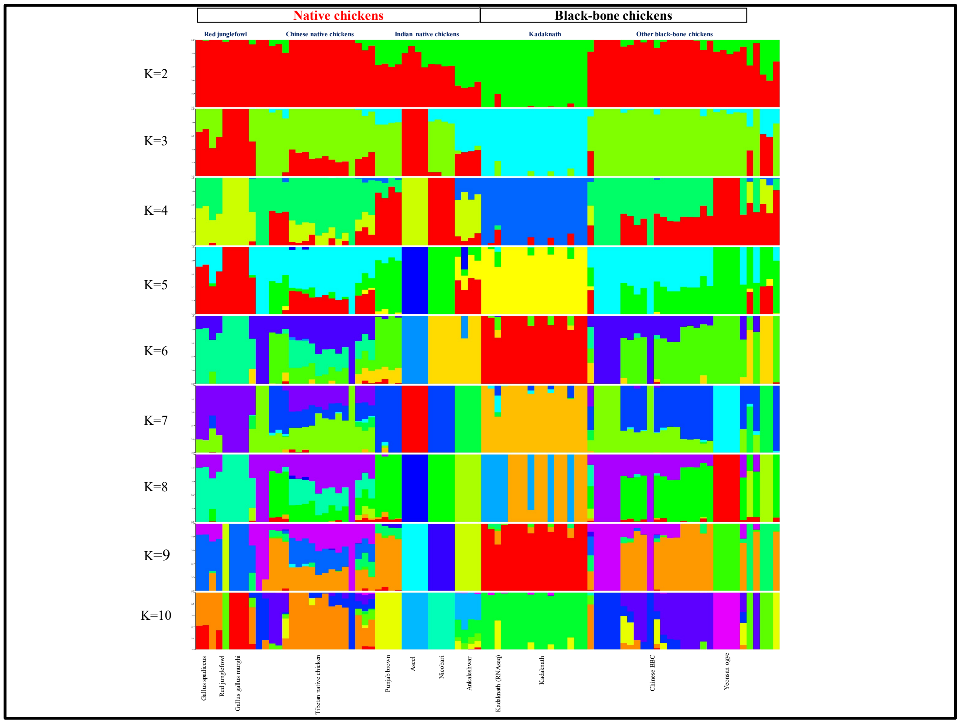

Supplementary Figure 21a. Population genetic structure and individual ancestry of 100 chicken individuals (after removing the 14 individuals of Rhode Island Red, 9 individuals of white leghorn, and 12 individuals of broiler) from different breeds were estimated using AdmixPipe v3.

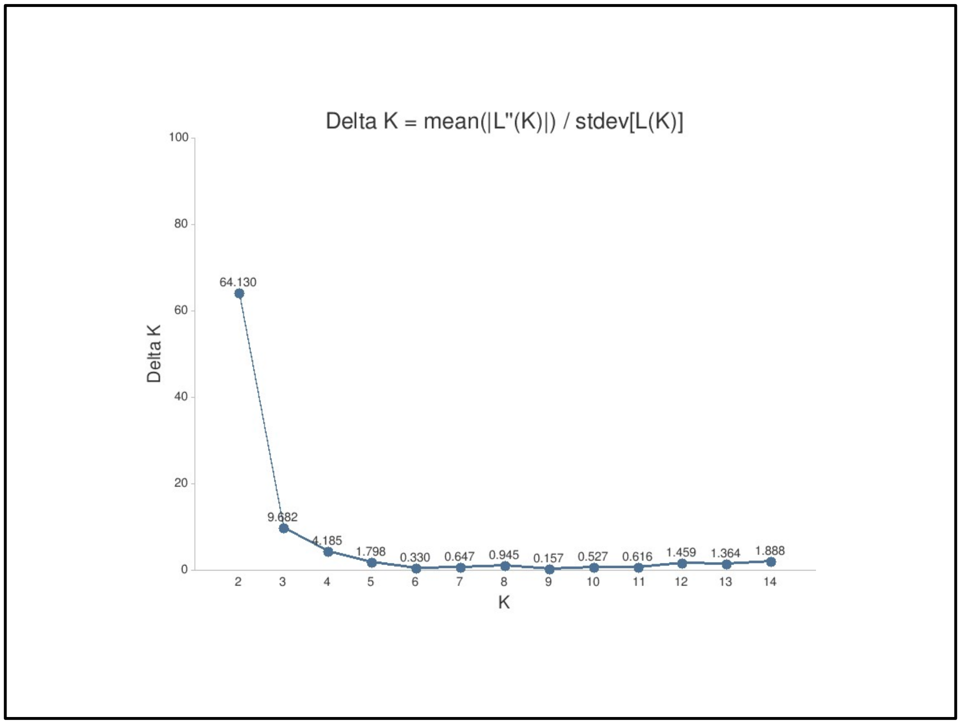

Supplementary Figure 21b. Delta K value plot for the admixture analysis of 88 individuals generated by CLUMPAK web server.

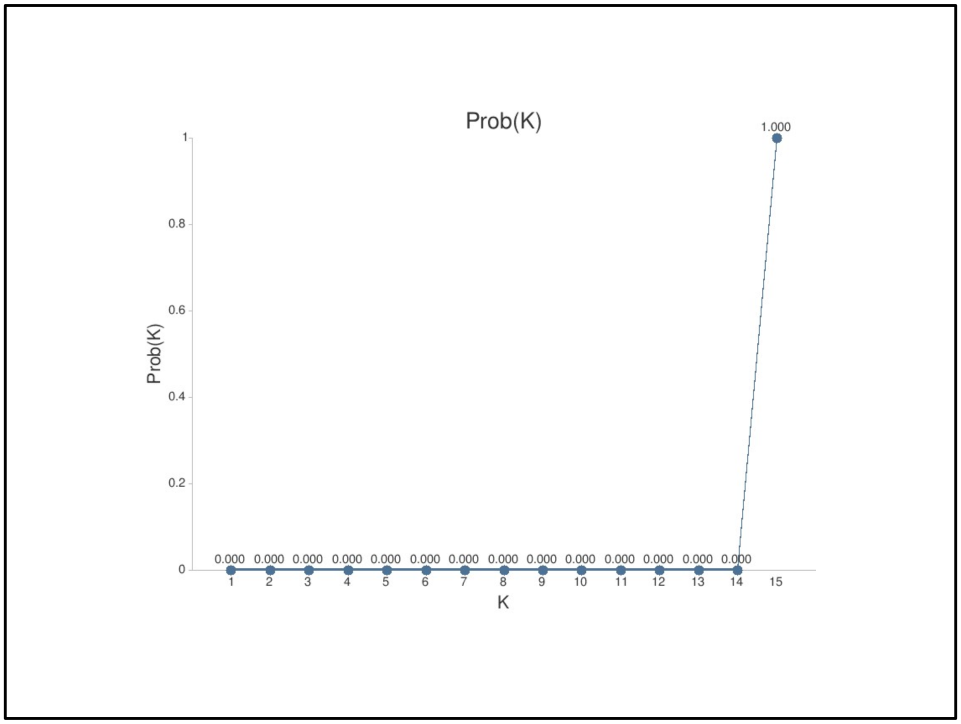

Supplementary Figure 21c. Log probability of the data referred to as ln Pr(X|K) value plot for the admixture analysis results of 88 individuals generated by CLUMPAK web server.

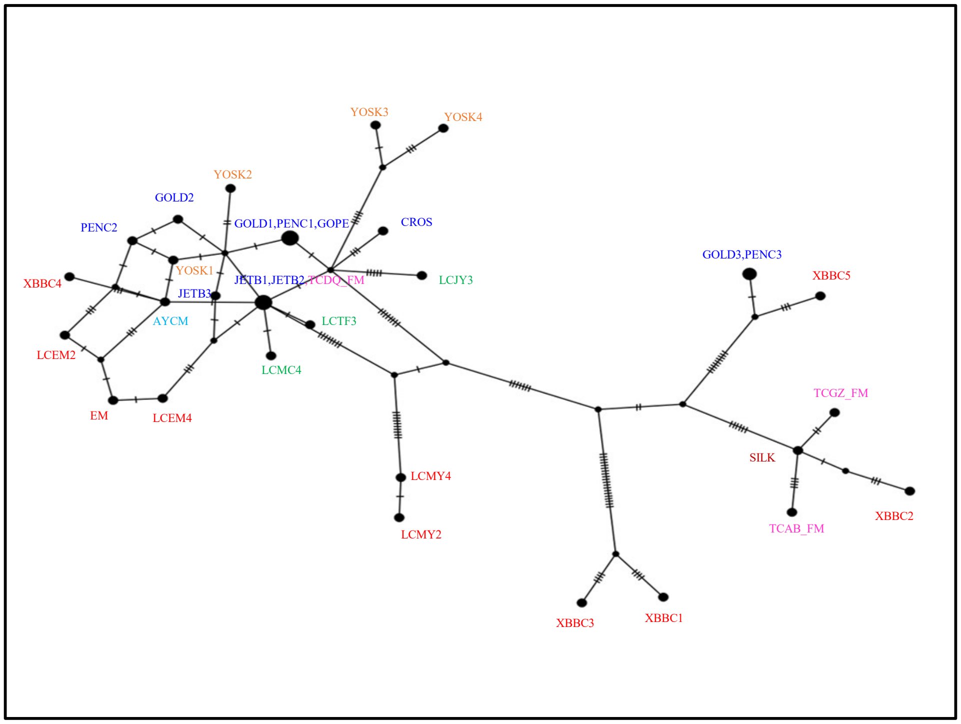

Supplementary Figure 22. PopART software is used for 33 black-bone chicken individuals for the whole mitochondrial network. Network constructed using the median-joining method.

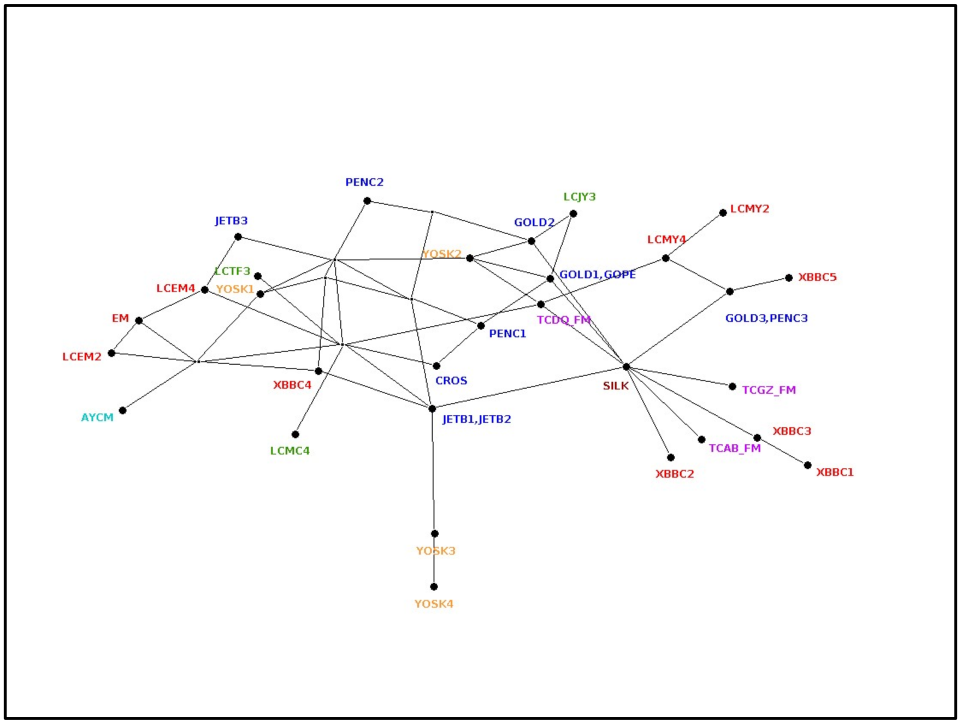

Supplementary Figure 23. SplitsTree software is used for 33 black-bone chicken individuals for the whole mitochondrial sequence. Network constructed using the median-joining method.

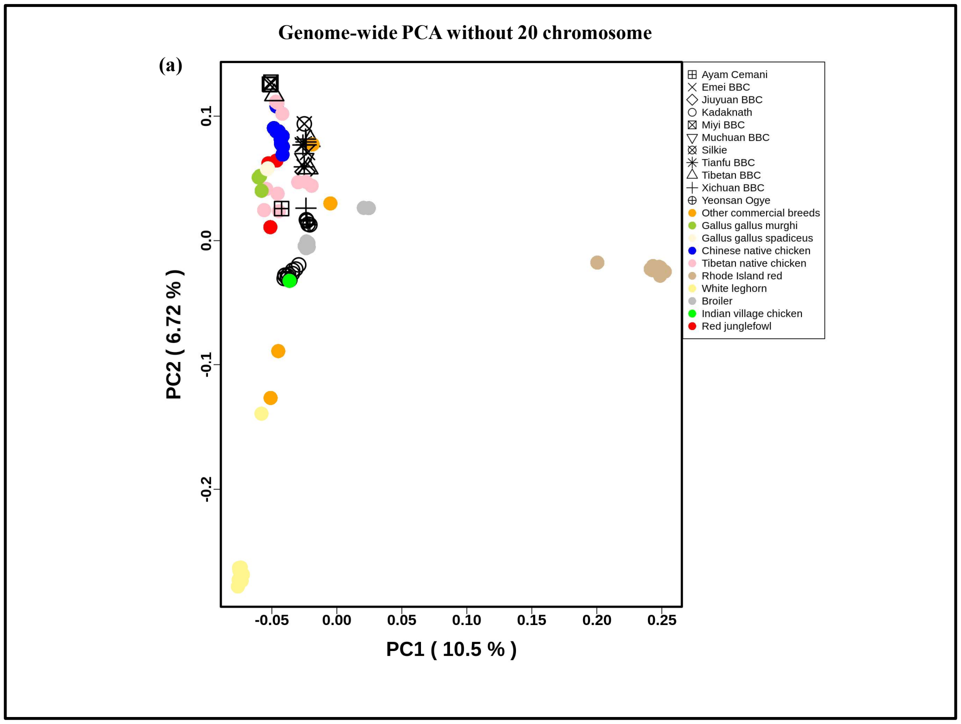

Supplementary Figure 24a. Genome-wide PCA plot generated for 101 chicken individuals: **(a)** PC1 and PC2 explained 10.5% and 6.72 % variance, respectively. Non-black-bone breeds are shown by different colors. All black-bone breeds are shown in black color with different shapes for each breed.

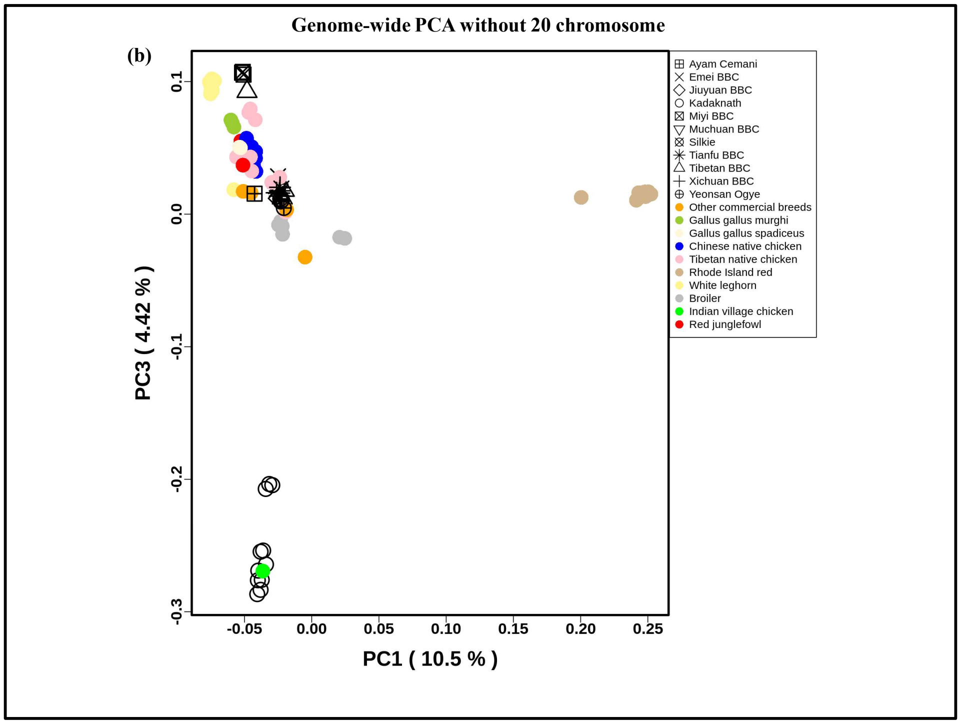

Supplementary Figure 24b. Genome-wide PCA plot generated for 101 chicken individuals: PC1 and PC3 explained 10.5% and 4.42% variance, respectively. Non-black-bone breeds are shown by different colors. All black-bone breeds are shown in black color with different shapes for each breed.

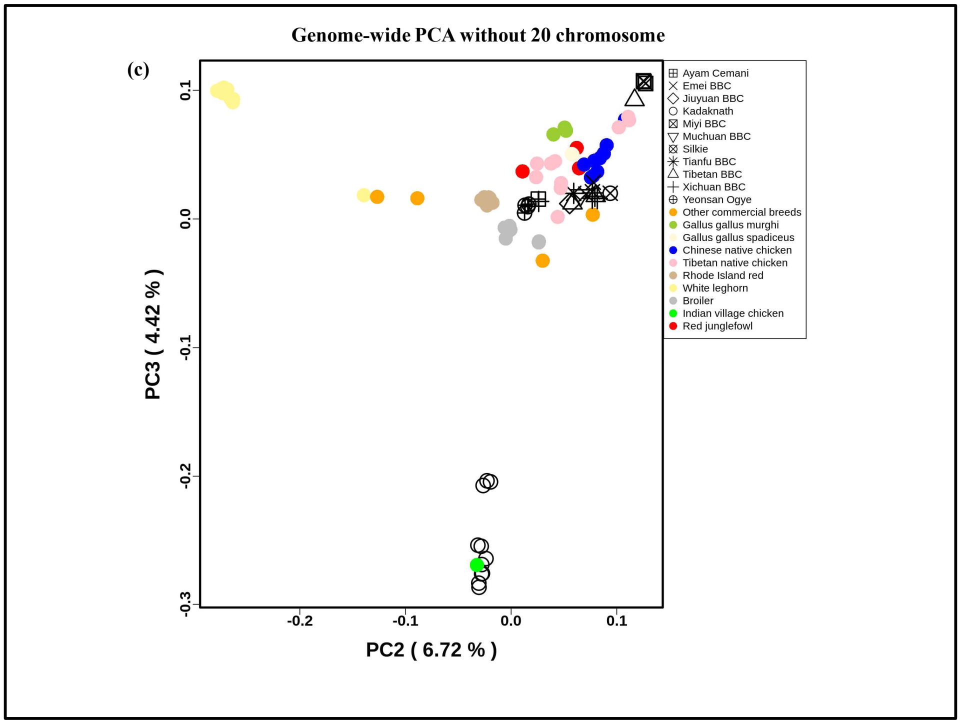

Supplementary Figure 24c. Genome-wide PCA plot generated for 101 chicken individuals: **(c)** PC2 and PC3 explained 6.72% and 4.42% variance, respectively. Non-black-bone breeds are shown by different colors. All black-bone breeds are shown in black color with different shapes for each breed.

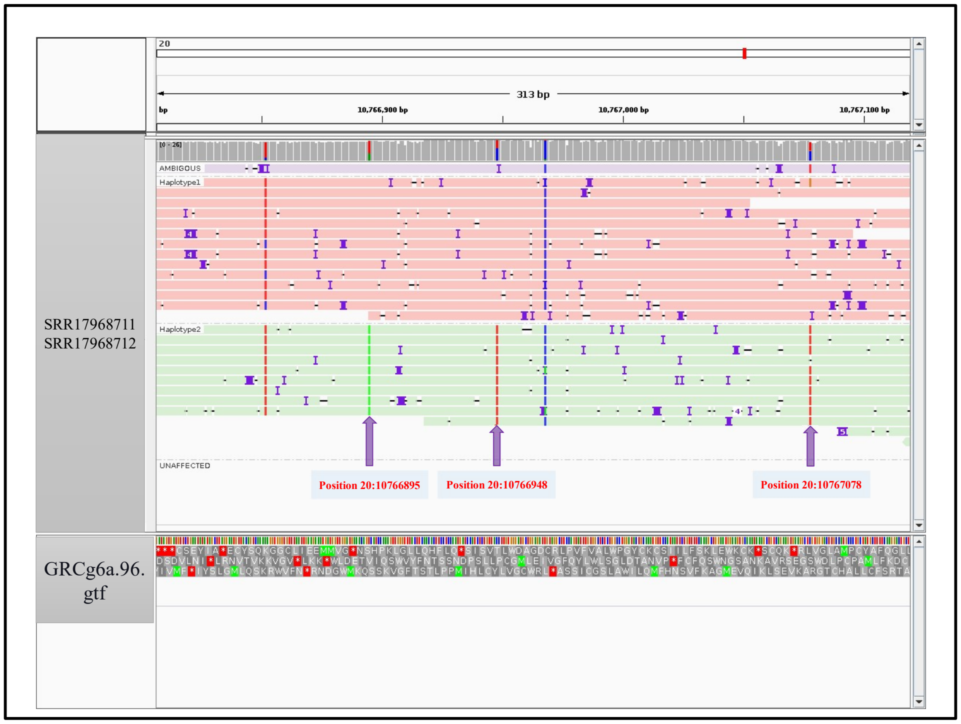

Supplementary Figure 25. IGV screenshot of haplotype-1 and haplotype-2 of Dup1 at position 10766895, 10766948, and 10767078 at chromosome 20. The long-read Nanopore data(SRR17968711, SRR17968712) of Silkie black bone chicken has been mapped to Gallus_gallus.GRCg6a genome assembly. Both haplotypes were differentiated based on the read group labeling using biostar214299, a program implemented in the jvarkit tool. Horizontal reads in light red represent haplotype-1 while reads in light green represent Haplotype2.

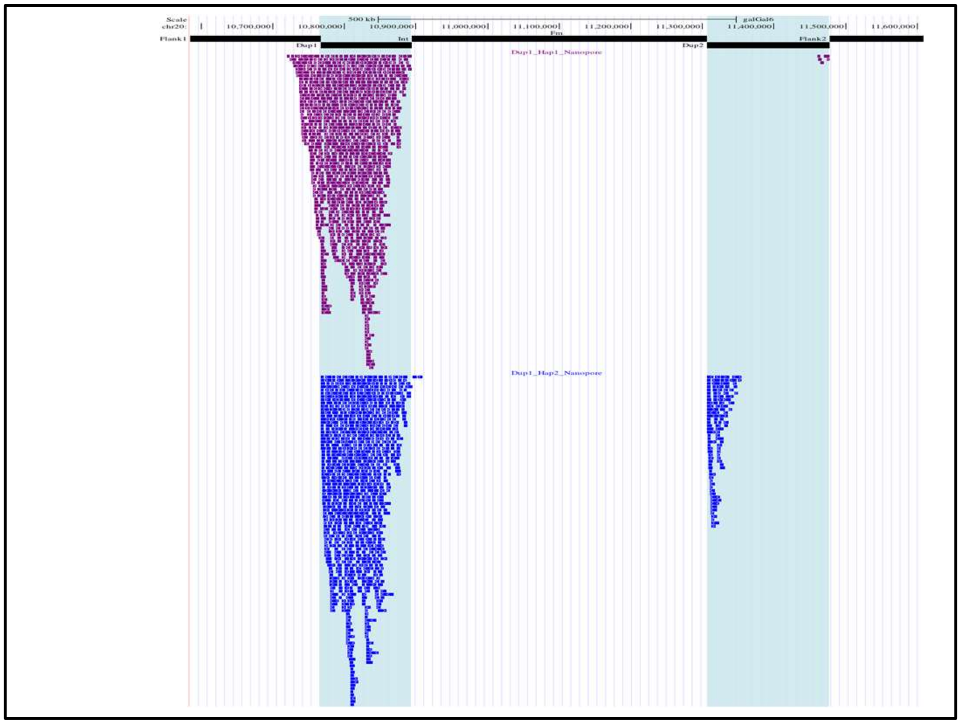

Supplementary Figure 26a. Screenshot of UCSC genome browser of two haplotype alleles at 24 positions of Dup1 region, which were identified using Nanopore long reads. Haplotype-1 of Dup1 spans from Flank1 to inverted Dup2 (i.e., Flank-1 + Dup1 + (inverted Dup2)). The purple lines represent overlapping reads of Nanopore containing Dup1 haplotype-1 alleles. The same purple lines represents the read from the end of haplotype-1 of Dup1 to the end of haplotype-2 of Dup2. Haplotype-2 spans Dup1 from haplotype-2 of Dup2 to the Int region (i.e., (inverted Dup2) + Dup1 + Int). The blue lines represent the overlapping reads of Nanopore containing haplotype-2 alleles. The same blue line represents the same read from the start of haplotype-2 of Dup1 to the start of haplotype-2 of Dup2. Both haplotypes of Dup1 are connected to the single haplotype of Dup2 (i.e., haplotype-2 of Dup2) but at different ends.

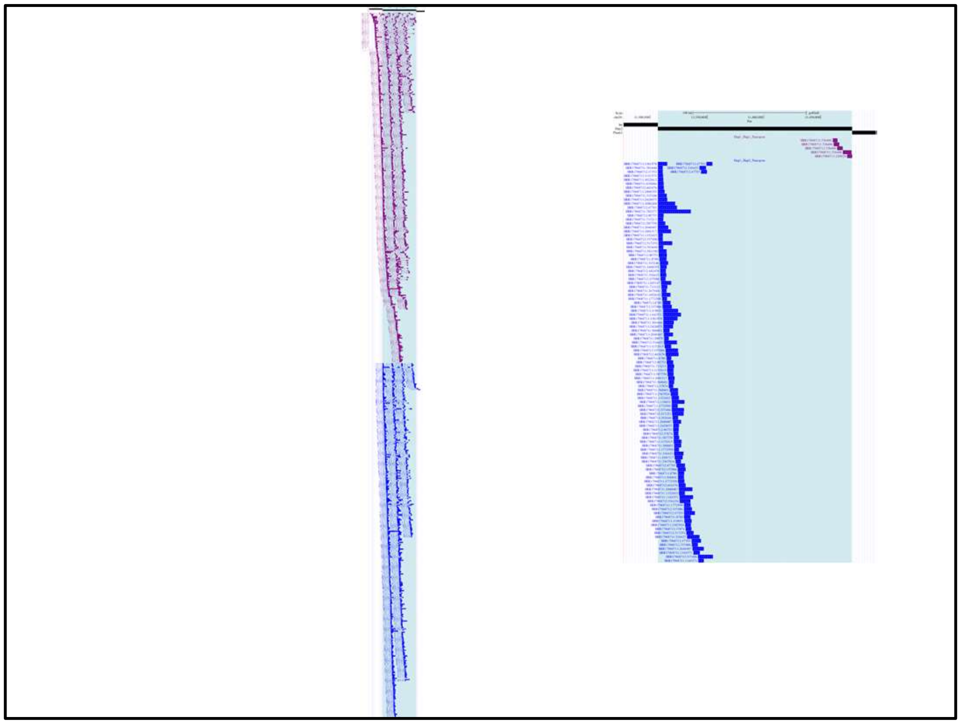

Supplementary Figure 26b. Zoomed view of Dup1 region haplotypes 1 and 2 with reads ids and same read at Dup2 region spans shown in Supplementary Figure 26a.

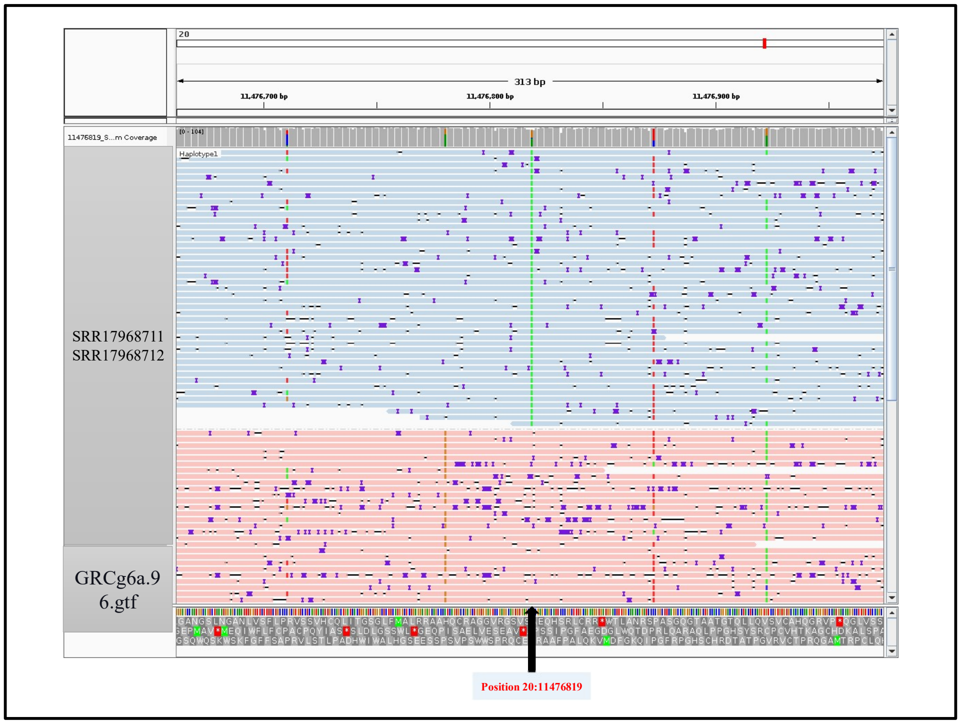

Supplementary Figure 27. IGV screenshot of haplotype-1 and haplotype-2 of Dup2 at position 11476819 at chromosome 20. The long-read Nanopore data(SRR17968711, SRR17968712) of Silkie black bone chicken has been mapped to Gallus_gallus.GRCg6a genome assembly. Both haplotypes were differentiated based on the read group labeling using biostar214299, a program implemented in the jvarkit tool. Horizontal reads in light blue represent haplotype-1 while reads in light red represent Haplotype2.

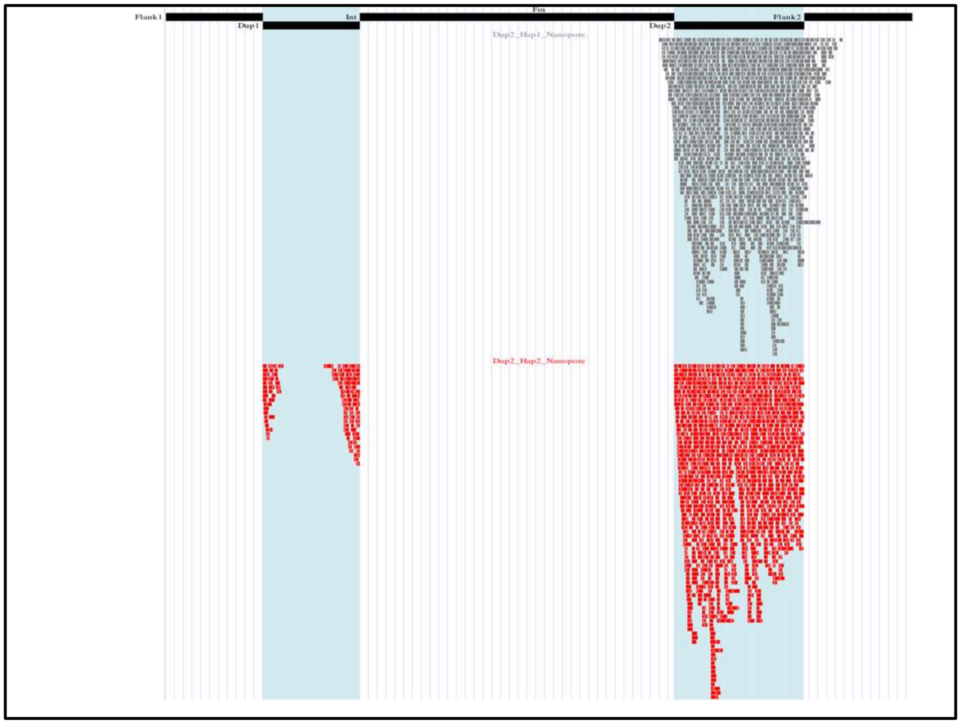

Supplementary Figure 28. Screenshot of UCSC genome browser of two haplotype alleles at 25 positions of Dup2 region, which were identified using Nanopore long reads. Haplotype-1 of Dup2 spans from Int to Flank2 (i.e., Int + Dup2 + Flank2). The grey lines represent overlapping reads of Nanopore containing Dup2 haplotype-1 alleles. Haplotype-2 of Dup2 spans from the start of haplotype-2 of Dup1 to the end of haplotype-1 of Dup1 region (i.e., Dup1 +(inverted Dup2)+ Dup1). The red lines represent the overlapping reads of Nanopore containing Dup2 haplotype-2 alleles. The same red line represents the span of the same reads from the start of haplotype-2 of Dup1 to the start of haplotype-2 of Dup2. Similarly, the same reads span the end of haplotype-1 of Dup1 to the end of haplotype-2 of Dup2.

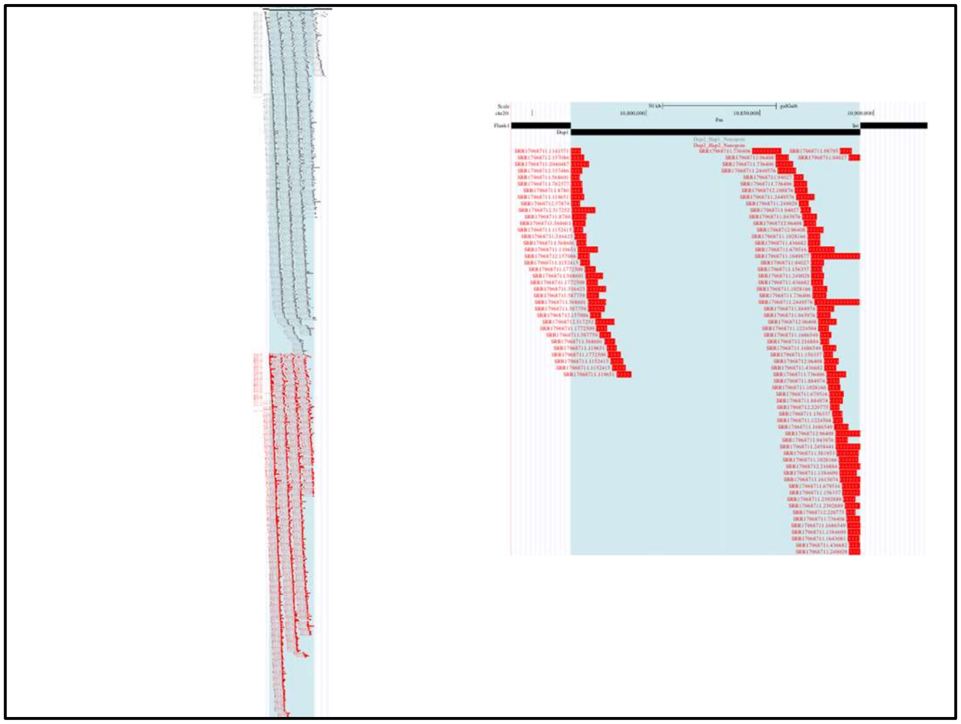

Supplementary Figure 29. Zoomed view of Dup2 region haplotypes 1 and 2 with reads ids and same read at Dup1 region spans shown in Supplementary Figure 28.

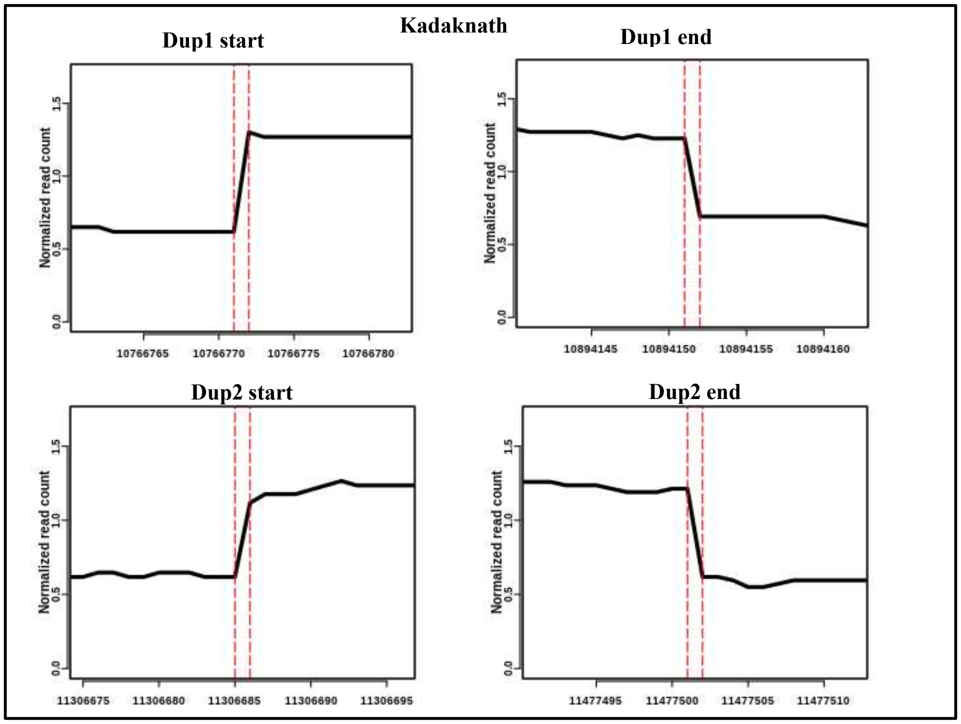

Supplementary Figure 30. Normalized read coverage at Dup1 and Dup2 region: Normalized read coverage at the base-pair level junction in Kadaknath (ERR9560156) for Dup1 start, Dup1 end, Dup2 start, and Dup2 end shown in the figure. The vertical red dotted line represents the exact position of the start and end of Dup1 and Dup2.

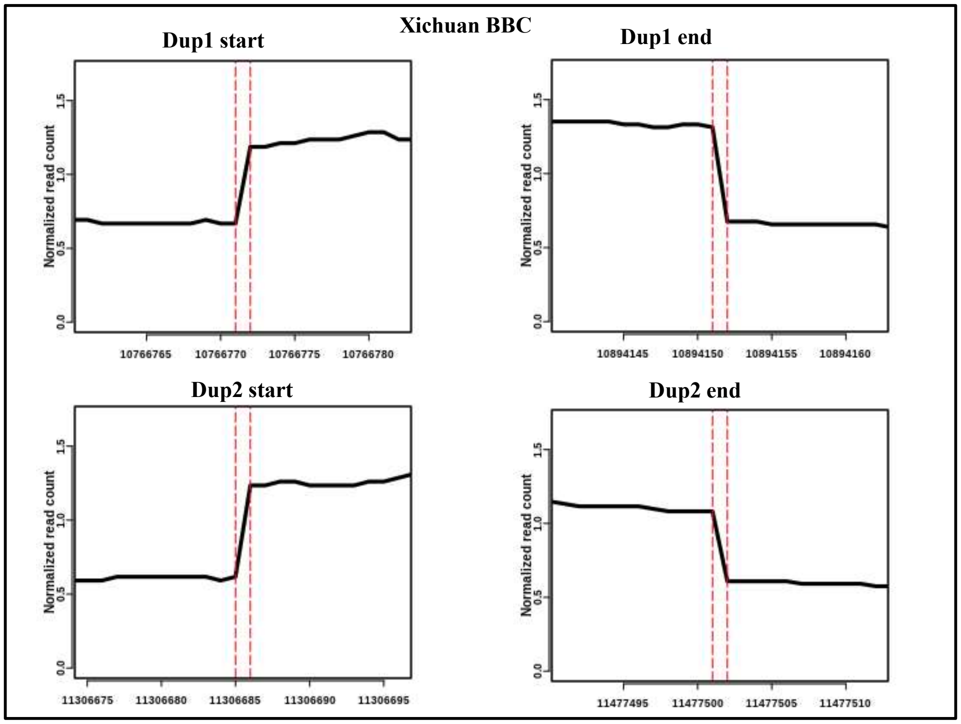

Supplementary Figure 31. Normalized read coverage at Dup1 and Dup2 region: Normalized read coverage at the base-pair level junction in Xichuan BBC (SRR12103812) for Dup1 start, Dup1 end, Dup2 start, and Dup2 end shown in the figure. The vertical red dotted line represents the exact position of the start and end of Dup1 and Dup2.

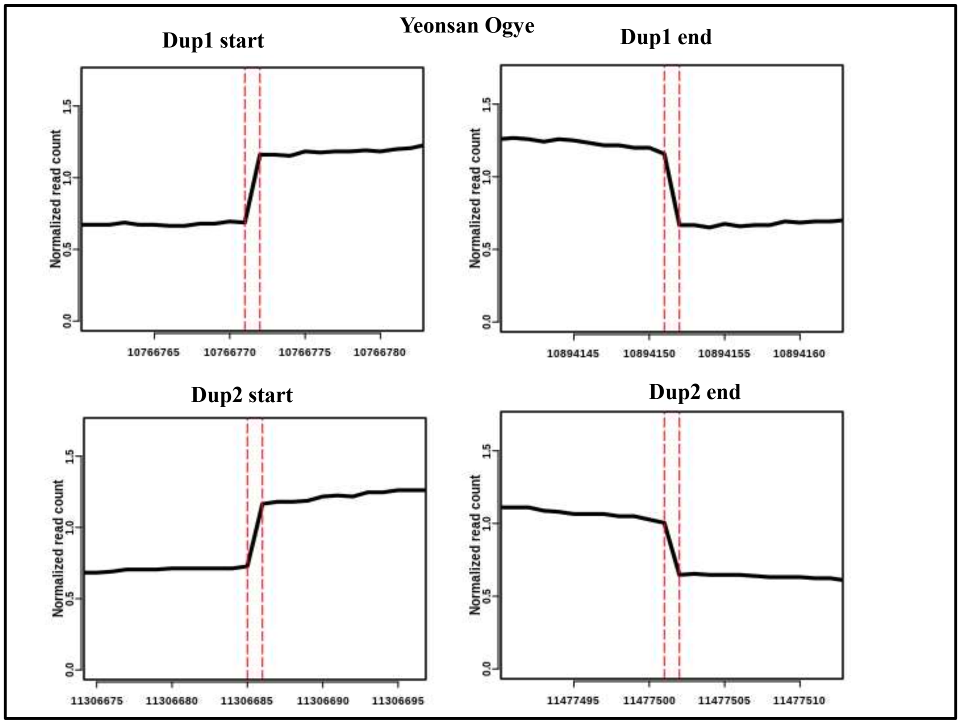

Supplementary Figure 32. Normalized read coverage at Dup1 and Dup2 region: Normalized read coverage at the base-pair level junction in Yeonsan Ogye (SRR6189094, SRR6189095, SRR6189096, SRR6189098, SRR6189082, SRR6189084 and SRR6189087) for Dup1 start, Dup1 end, Dup2 start, and Dup2 end shown in the figure. The vertical red dotted line represents the exact position of the start and end of Dup1 and Dup2.

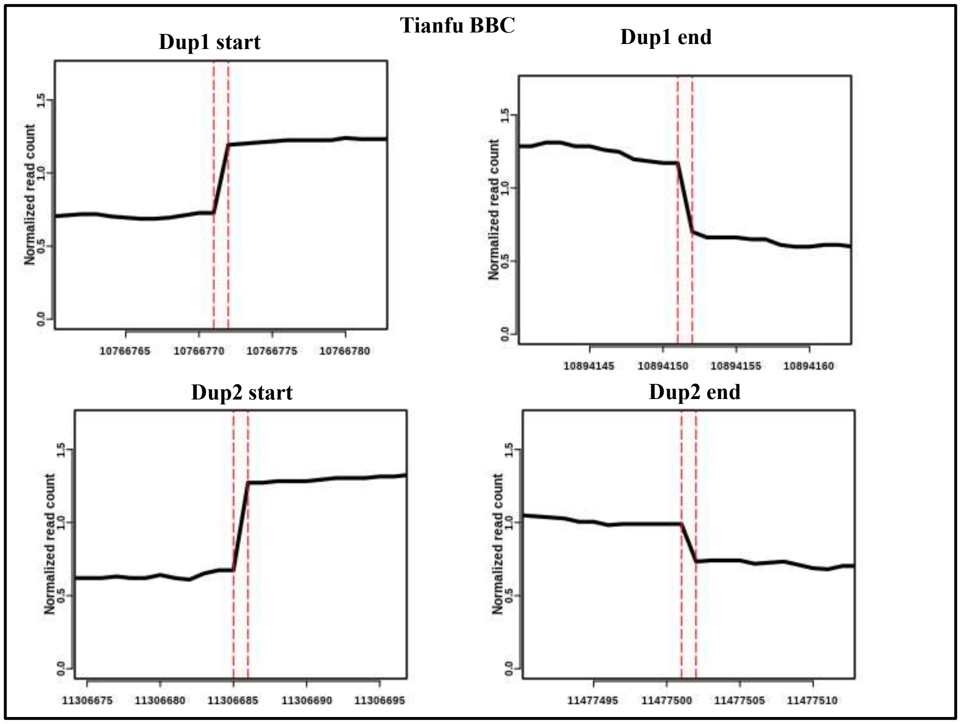

Supplementary Figure 33. Normalized read coverage at Dup1 and Dup2 region: Normalized read coverage at the base-pair level junction in Tianfu BBC (SRR3041425) for Dup1 start, Dup1 end, Dup2 start, and Dup2 end shown in the figure. The vertical red dotted line represents the exact position of the start and end of Dup1 and Dup2.

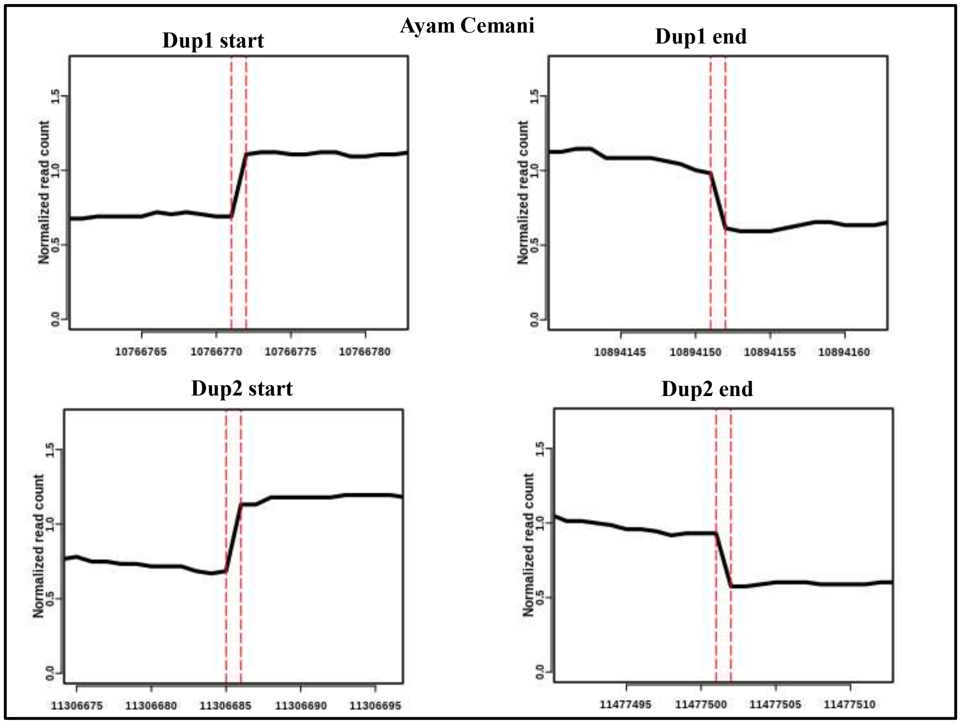

Supplementary Figure 34. Normalized read coverage at Dup1 and Dup2 region: Normalized read coverage at the base-pair level junction in Ayam Cemani (SRR17916231) for **(A)** Dup1 start, Dup1 end, Dup2 start, and Dup2 end shown in the figure. The vertical red dotted line represents the exact position of the start and end of Dup1 and Dup2.

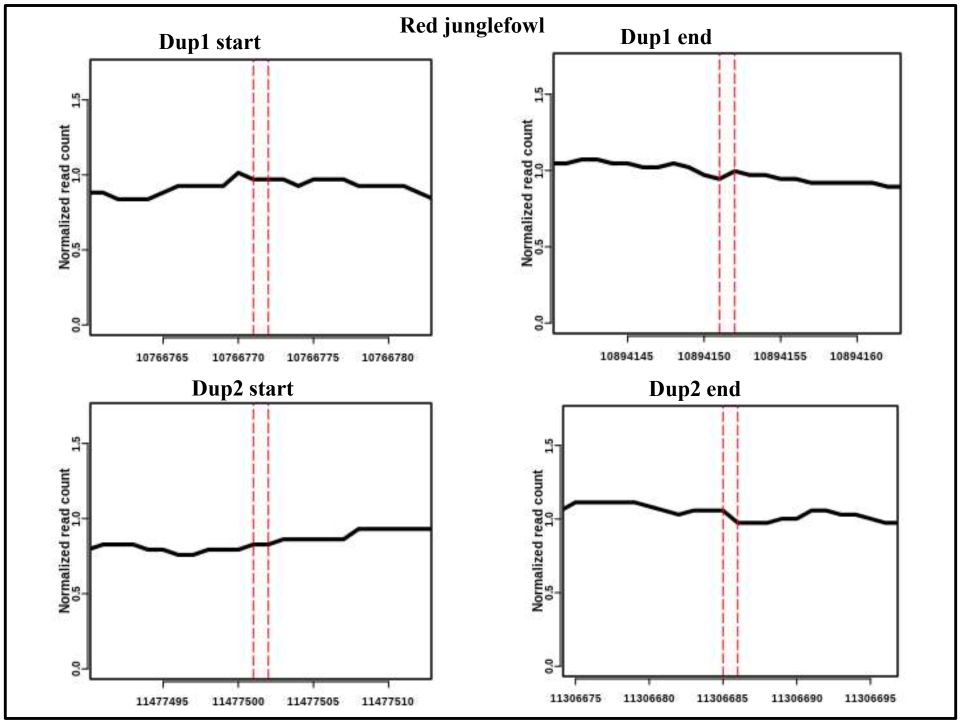

Supplementary Figure 35. Normalized read coverage at Dup1 and Dup2 region: Normalized read coverage at the base-pair level junction in Red junglefowl (SRR3954707) for Dup1 start, Dup1 end, Dup2 start, and Dup2 end shown in the figure. The vertical red dotted line represents the exact position of the start and end of Dup1 and Dup2.

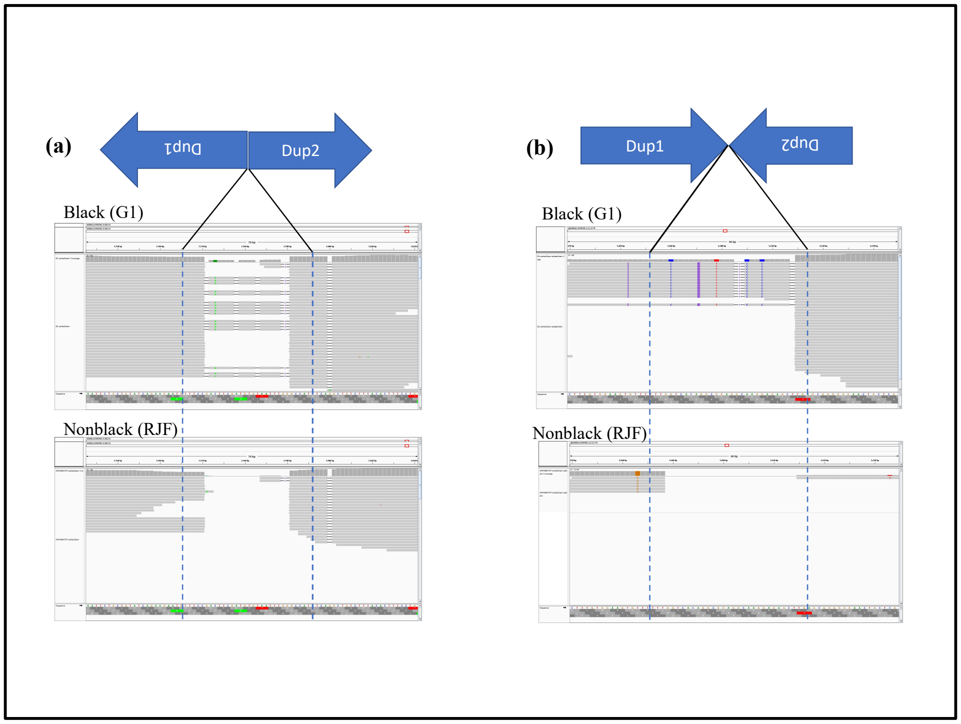

Supplementary Figure 36. Two rearranged junctions in black chicken: **(a)** inverted_Dup1 Dup2 junction where the blue arrows show the direction of rearrangement, the Dup1 is inverted and Dup2 without inversion. In this region, we mentioned the black and nonblack chicken. On the top panel, a black Kadaknath (Golden1) IGV screenshot of rearranged junction supported reads, while the bottom nonblack (Red junglefowl) chicken without any reads support. The blue dotted line represents the junction region. **(b)** dup1 dup2_inverted in the second junction where the arrows show rearrangement directions, dup1 is without inversion, whereas dup2 is inverted. The top panel defines a black chicken IGV screenshot of reads support to the junction. A bottom panel of nonblack chicken with no read support for the junction in the IGV screenshot.

(a) **Dup1 Dup2_inverted junction in BBC**

Kadaknath
SRA read id :A00609:130:HHL7CDSXY:4:2451:11948:27571 1:N:0:GTGACGAT+TCTGAAGG
TACCCAGCGCTTTGTGGAAGTGGTTTTTTGCTCACAGGGGCTGAGCAGCAGAGCTGAGCG

Yeonsan Ogye
SRA read id :SRR6189084.3191401 3191401/1
TACCCAGCGCTTTGTGGAAGTGGTTTTTTGCTCACAGGGGCTGAGCAGCAGAGCTGAGCG

Xichuan BBC
SRA read id :SRR12103812.63579191 63579191/1
TACCCAGCGCTTTGTGGAAGTGGTTTTTTGCTCACAGGGGCTGAGCAGCAGAGCTGAGCG

Ayam Cemani
SRA read id :SRR17916231.85870834 85870834/1
TACCCAGCGCTTTGTGGAAGTGGTTTTTTGCTCACAGGGGCTGAGCAGCAGAGCTGAGCG

Tianfu BBC
SRA read id :SRR3041425.14780543 14780543/1
TACCCAGCGCTTTGTGGAAGTGGTTTTTTGCTCACAGGGGCTGAGCAGCAGAGCTGAGCG

(b) **Start of Dup1 and Dup2 in Non-BBC breeds**

Red junglefowl Dup1
SRA read id :SRR3954707.49036966 49036966/1
TACCCAGCGCTTTGTGGAAGTGGTTTTTTGCCTCCTTTCTGGCCCTCCCTAAGAGCGCTT

Red junglefowl inverted Dup2
SRA read id :SRR3954707.63570588 63570588/1
CCCGCTGTCAGACCGTGGGGACAGCAGGGACTCACAGGGGCTGAGCAGCAGAGCTGAGCG

Broiler Dup1
SRA read id :A00129:525:HMHT2DSXX:1:1429:26946:5102 1:N:0:TTCCTGGA+CAGACGTA
TACCCAGCGCTTTGTGGAAGTGGTTTTTTGCCTCCTTTCTGGCCCTCCCTAAGAGCGCTT

Broiler inverted Dup2
SRA read id :A00129:525:HMHT2DSXX:1:1224:23863:3521 1:N:0:TTCCTGGA+CAGACGTA
CCCGCTGTCAGACCGTGGGGACAGCAGGGACTCACAGGGGCTGAGCAGCAGAGCTGAGCA

Gallus gallus murghi Dup1
SRA read id :SRR12868131.39789528 39789528/1
TACCCAGCGCTTTGTGGAAGTGGTTTTTTGCCTCCTTTCTGGCCCTCCCTAAGAGCGCTT

Gallus gallus murghi inverted Dup2
SRA read id :SRR12868131.1086926 1086926/1
CCCGCTGTCAGACCGTGGGGACAGCAGGGACTCACAGGGGCTGAGCAGCAGAGCTGAGCG

Tibetan native chicken Dup1
SRA read id :SRR3041438.14333875 14333875/1
TACCCAGCGCTTTGTGGAAGTGGTTTTTTGCCTCCTTTCTGGCCCTCCCTAAGAGCGCTT

Tibetan native chicken inverted Dup2
SRA read id :SRR3041438.21220905 21220905/1
CCCGCTGTCAGACCGTGGGGACAGCAGGGACTCACAGGGGCTGAGCAGCAGAGCTGAGCG

Supplementary Figure 37. (a) Dup1 Dup2 inverted junction in black-bone chicken breeds **(b)** Dup1 and Dup2 in non-BBC breeds. The reads containing the start of Dup1 and Dup2 have been shown along with their SRA id. The nucleotide sequences highlighted in red and blue represent Dup1 and Dup2, respectively, which is present as continuous sequence BBC breeds. Sequences in black color represent subsequent nucleotides.

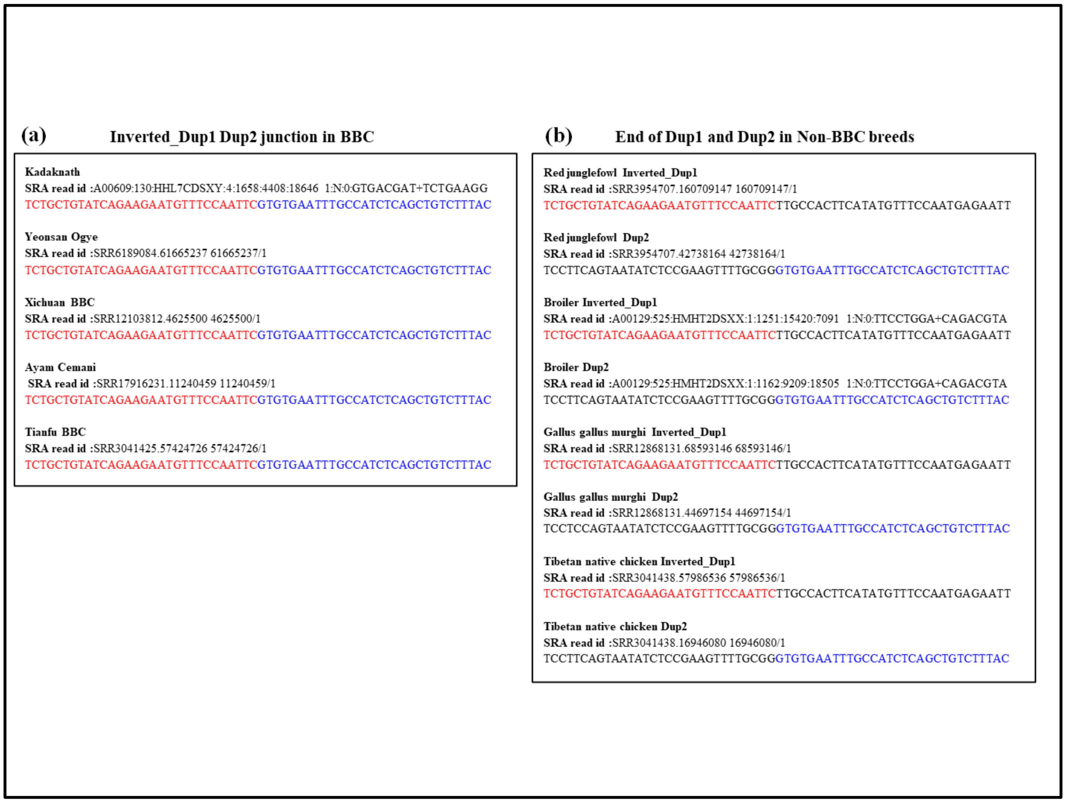

Supplementary Figure 38. (a) Inverted Dup1 Dup2 junction in black-bone chicken breeds **(b)** Dup1 and Dup2 in non-BBC breeds. The reads containing the end of Dup1 and Dup2 have been shown along with their SRA id. The nucleotide sequences highlighted in red and blue represent Dup1 and Dup2, respectively, which is present as continuous sequence BBC breeds. Sequences in black color represent subsequent nucleotides.

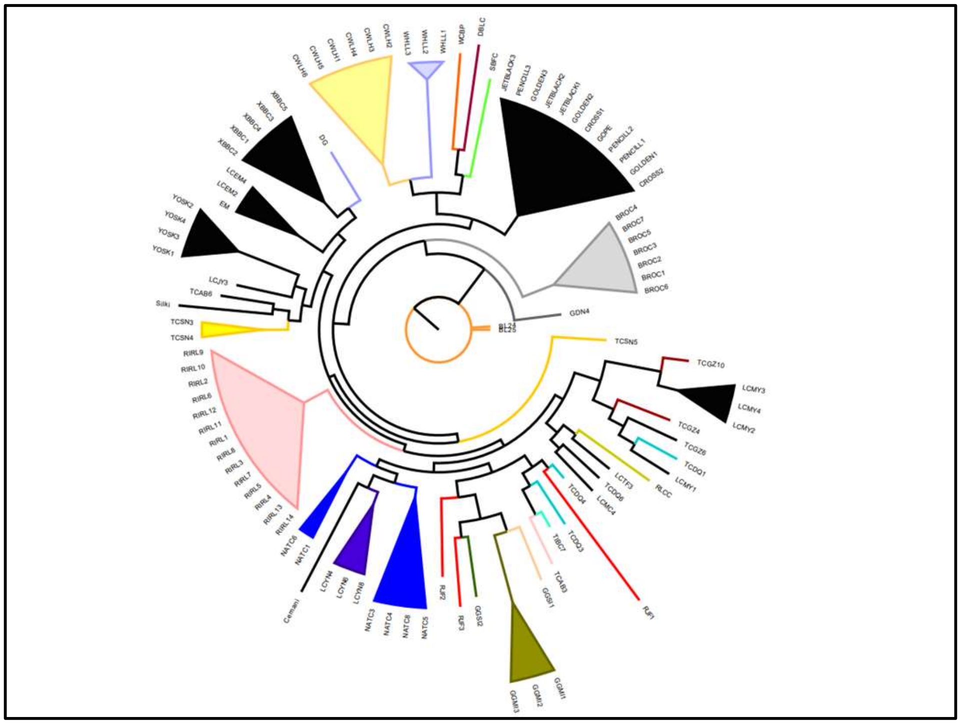

Supplementary Figure 39. Genome-wide phylogeny of 101 individuals using variant call from bcftools. The phylogeny is generated using SNPhylo and visualized in Figtree.

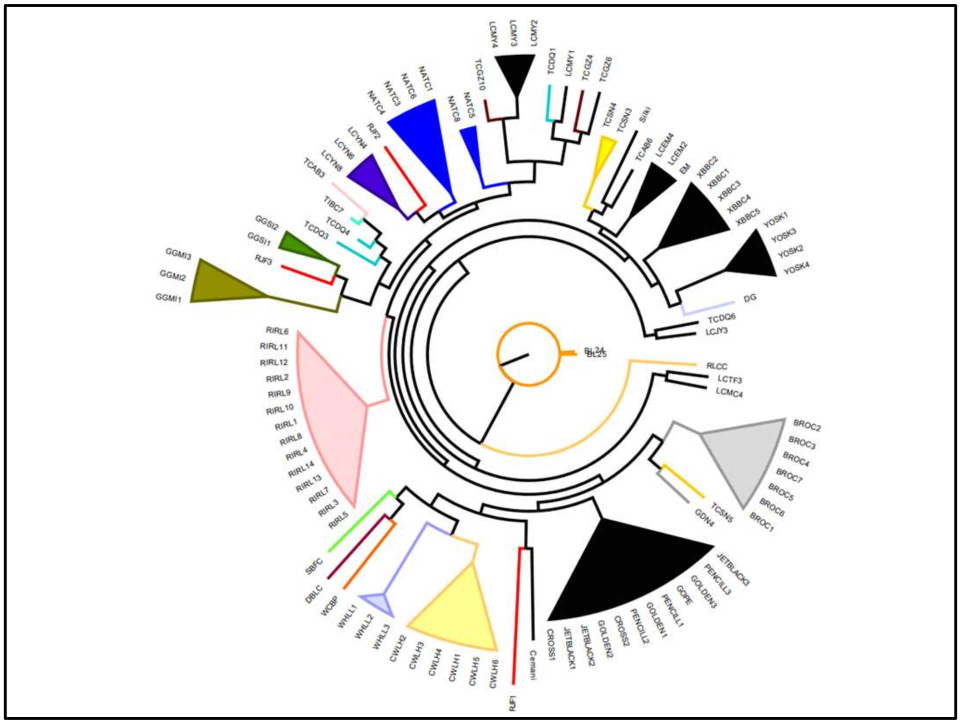

Supplementary Figure 40. Genome-wide phylogeny of 101 individuals using variant call from freebayes. The phylogeny is generated using SNPhylo and visualized in Figtree.

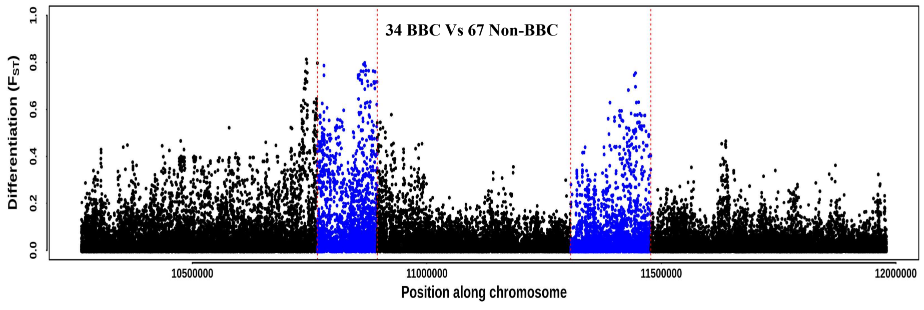

Supplementary Figure 41. F_{ST} landscape of *Fm* locus between 34 black-bone chicken (BBC) and 67 non-black-bone chickens (non-BBC) is shown in a 1bp windows. Dup1 and Dup2 regions are represented in blue color dots, showing high F_{ST} compared to the nearby regions.

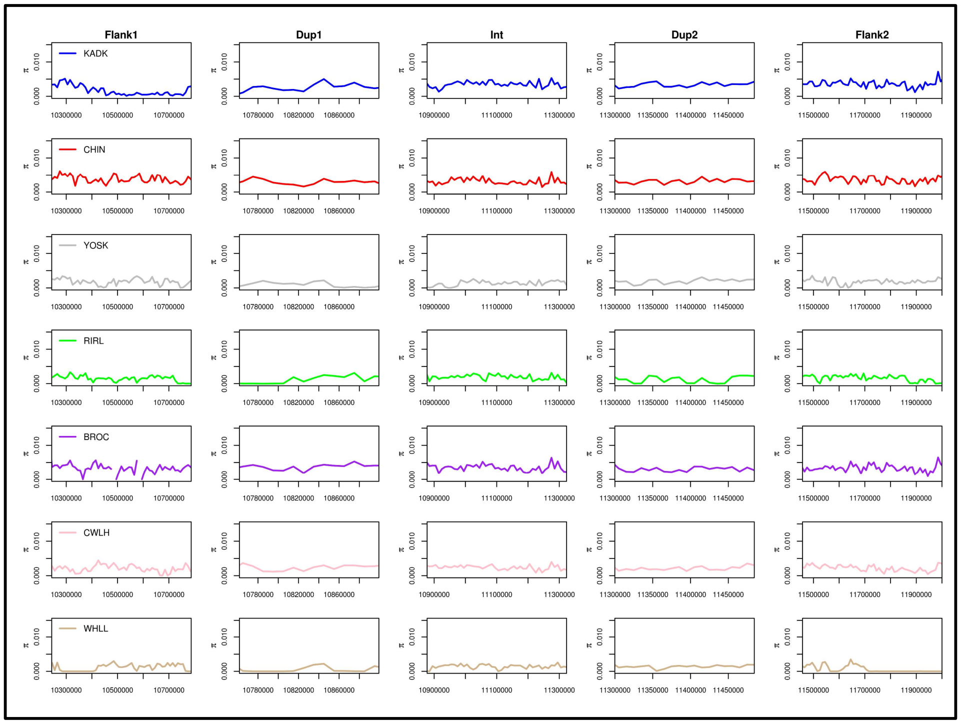

Supplementary Figure 42. Nucleotide diversity (**π**) at *Fm* locus regions (Flank1, Dup1, Int, Dup2, and Flank2) has been shown for each breed represented in different colors using 10Kb sliding windows.

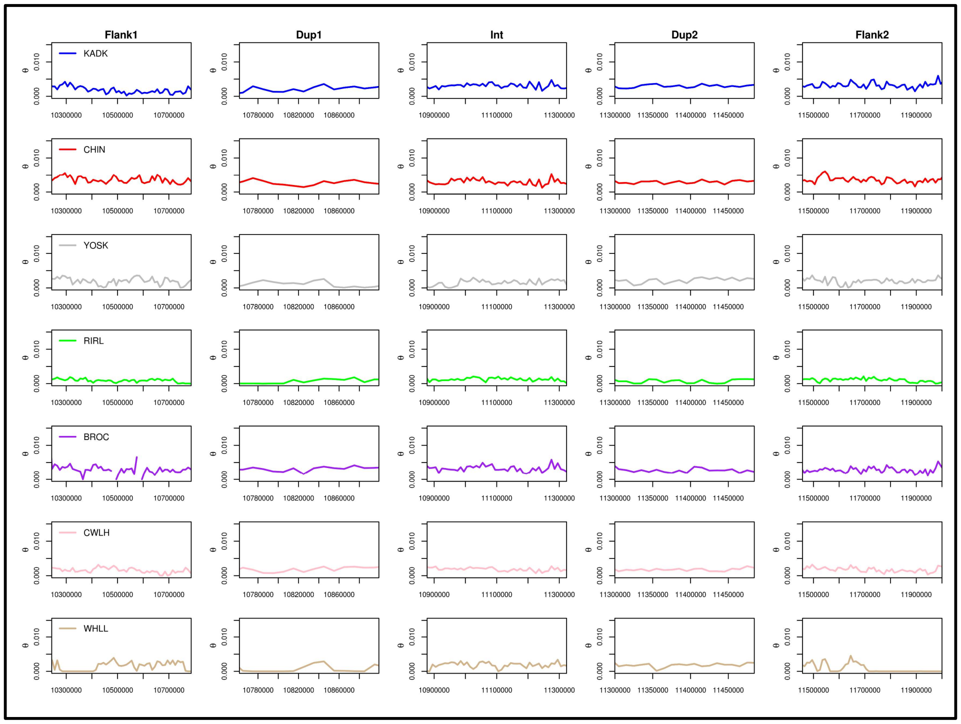

Supplementary Figure 43. Estimated Genetic diversity (**θ**) at *Fm* locus regions (Flank1, Dup1, Int, Dup2, and Flank2) has been shown for each breed represented in different colors using 10Kb sliding windows.

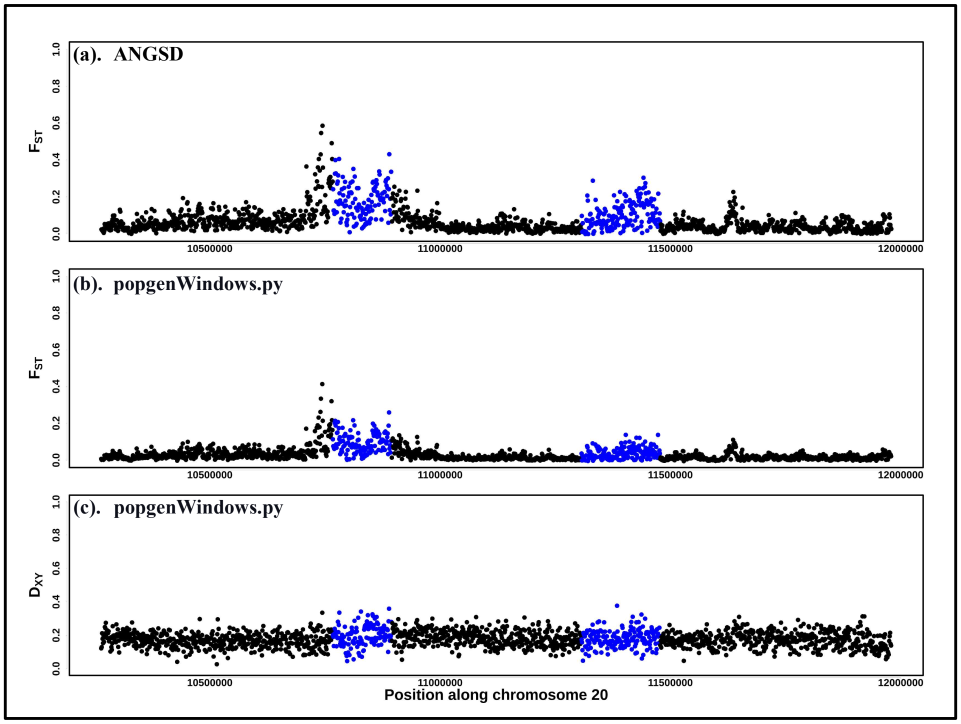

Supplementary Figure 44. Pairwise F_{ST} estimation at *Fm* locus between 34 black-bone and 67 non-black-bone chickens is shown in a 1Kb window calculated using **(a)** ANGSD, **(b)** popgenWindows.py, and **(c)** Dxy using popgenWindows.py. Dup1 and Dup2 regions are represented in blue color dots, having high F_{ST} compared to the nearby regions.

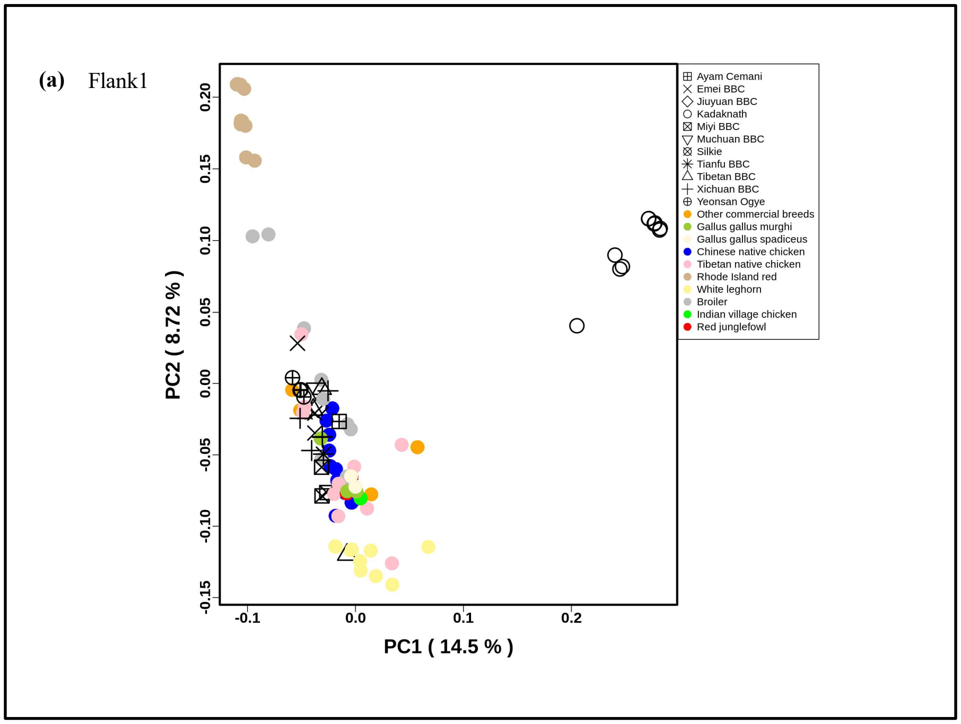

Supplementary Figure 45a. Principal component analysis (PCA) of *Fm* region for 101 chicken individuals with the first two principal groups (PC1 and PC2). Flank1 . Non-black-bone breeds are shown in different colors, while all black-bone breeds are shown in black with different shapes for each breed.

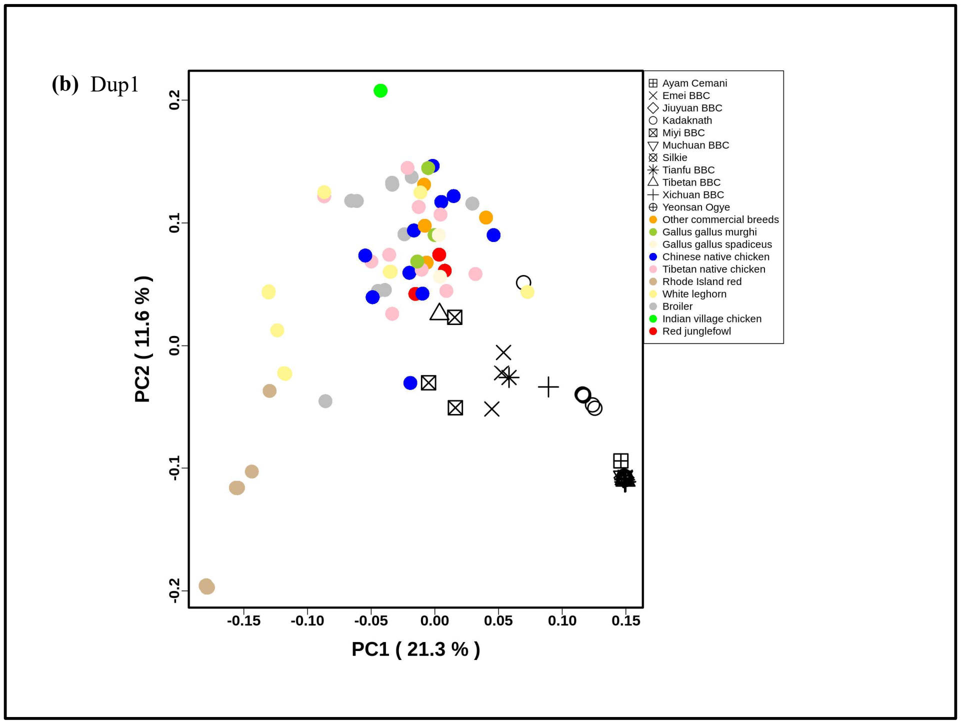

Supplementary Figure 45b. Principal component analysis (PCA) of *Fm* region for 101 chicken individuals with the first two principal groups (PC1 and PC2). Dup1. Non-black-bone breeds are shown in different colors, while all black-bone breeds are shown in black with different shapes for each breed.

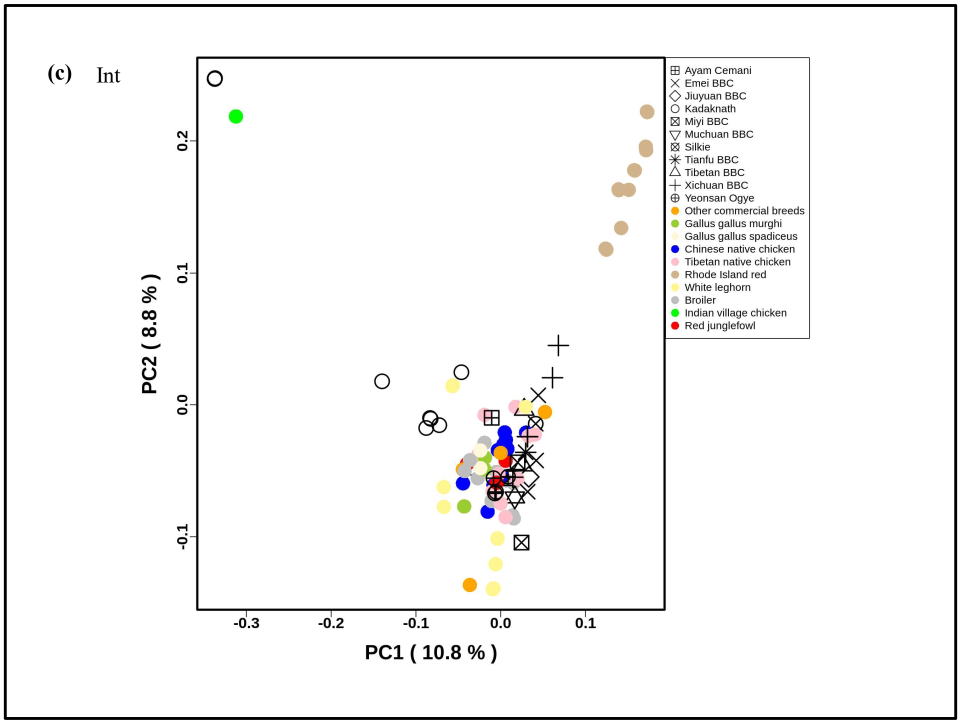

Supplementary Figure 45c. Principal component analysis (PCA) of *Fm* region for 101 chicken individuals with the first two principal groups (PC1 and PC2). Int. Non-black-bone breeds are shown in different colors, while all black-bone breeds are shown in black with different shapes for each breed.

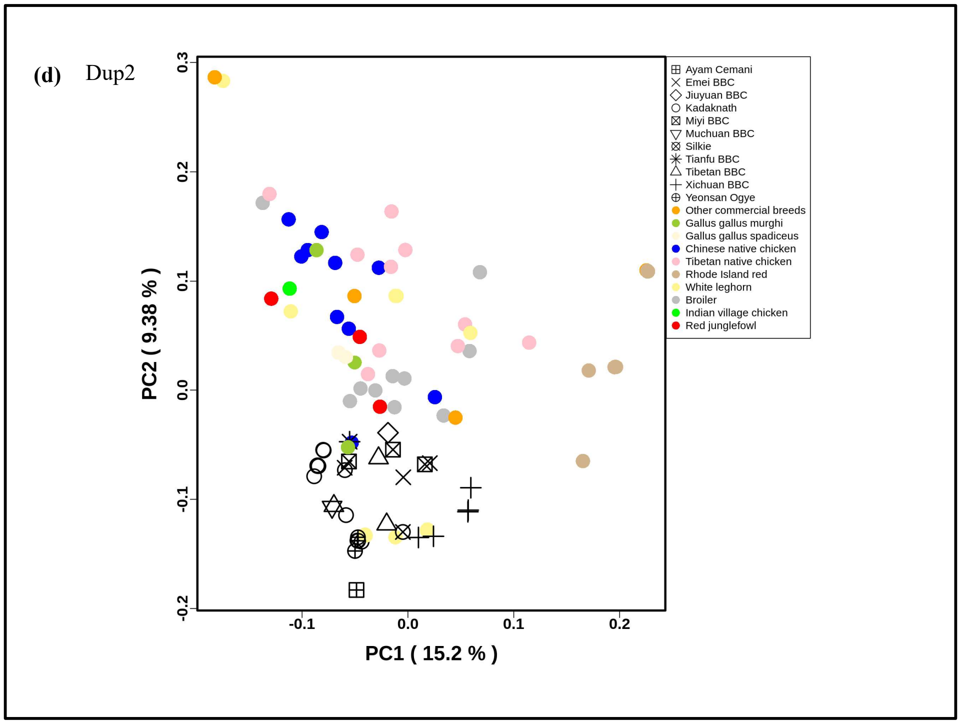

Supplementary Figure 45d. Principal component analysis (PCA) of *Fm* region for 101 chicken individuals with the first two principal groups (PC1 and PC2). Dup2. Non-black-bone breeds are shown in different colors, while all black-bone breeds are shown in black with different shapes for each breed.

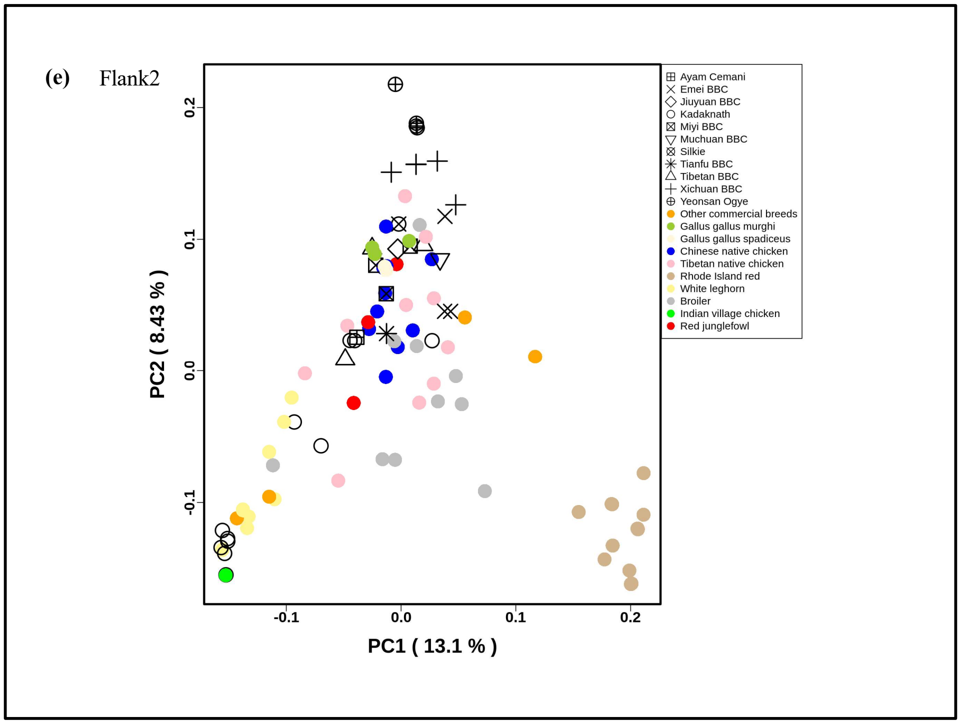

Supplementary Figure 45e. Principal component analysis (PCA) of *Fm* region for 101 chicken individuals with the first two principal groups (PC1 and PC2). Flank2. Non-black-bone breeds are shown in different colors, while all black-bone breeds are shown in black with different shapes for each breed.

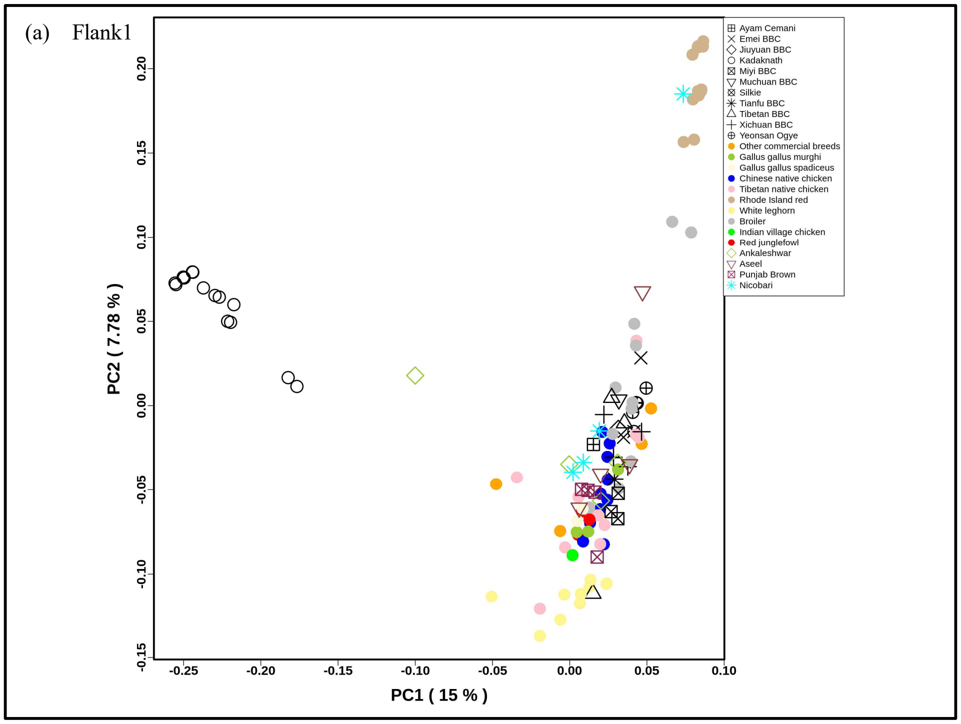

Supplementary Figure 46a. Principal component analysis (PCA) of *Fm* region for 123 chicken individuals with the first two principal groups (PC1 and PC2). Flank1. Non-black-bone breeds are shown in different colors, while all black-bone breeds are shown in black with different shapes for each breed.

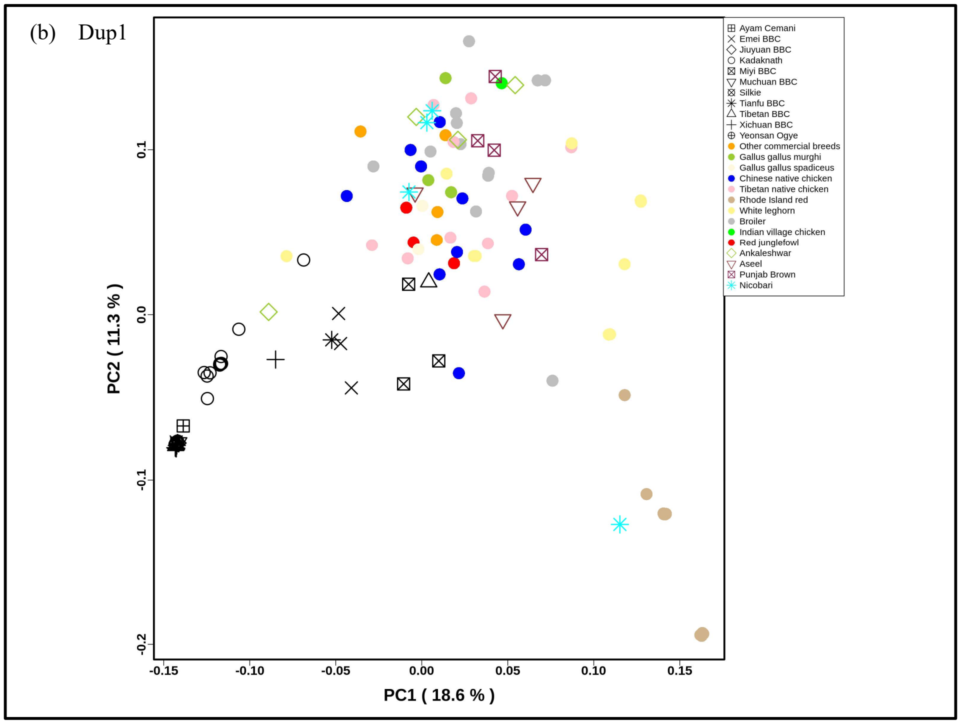

Supplementary Figure 46b. Principal component analysis (PCA) of *Fm* region for 123 chicken individuals with the first two principal groups (PC1 and PC2). Dup1. Non-black-bone breeds are shown in different colors, while all black-bone breeds are shown in black with different shapes for each breed.

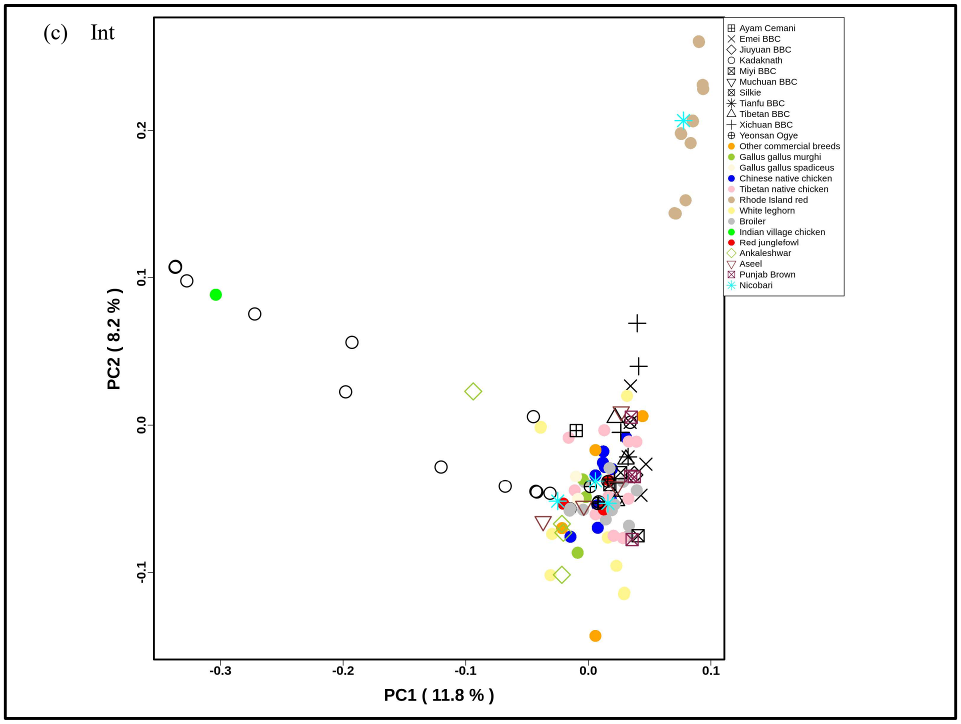

Supplementary Figure 46c. Principal component analysis (PCA) of *Fm* region for 123 chicken individuals with the first two principal groups (PC1 and PC2). Int. Non-black-bone breeds are shown in different colors, while all black-bone breeds are shown in black with different shapes for each breed.

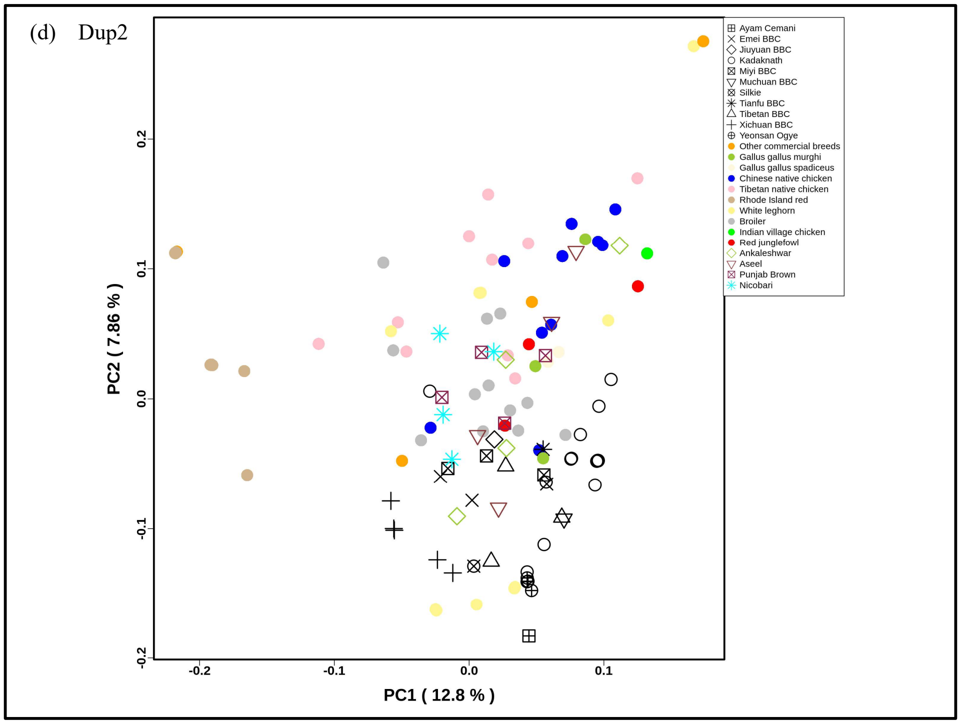

Supplementary Figure 46d. Principal component analysis (PCA) of *Fm* region for 123 chicken individuals with the first two principal groups (PC1 and PC2). Dup2. Non-black-bone breeds are shown in different colors, while all black-bone breeds are shown in black with different shapes for each breed.

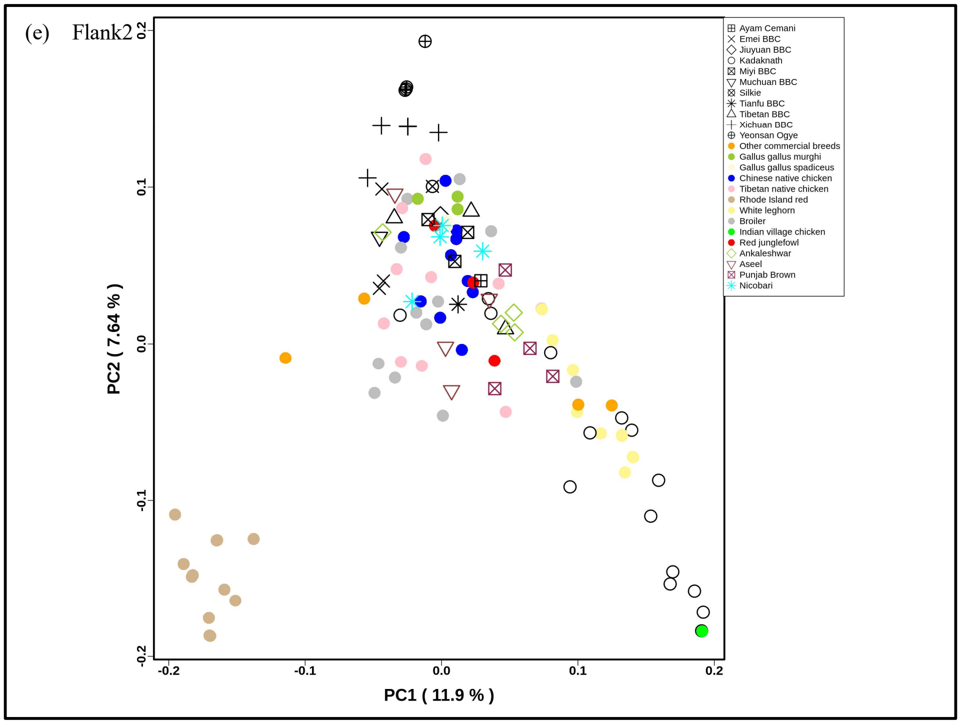

Supplementary Figure 46e. Principal component analysis (PCA) of *Fm* region for 123 chicken individuals with the first two principal groups (PC1 and PC2). Flank2. Non-black-bone breeds are shown in different colors, while all black-bone breeds are shown in black with different shapes for each breed.

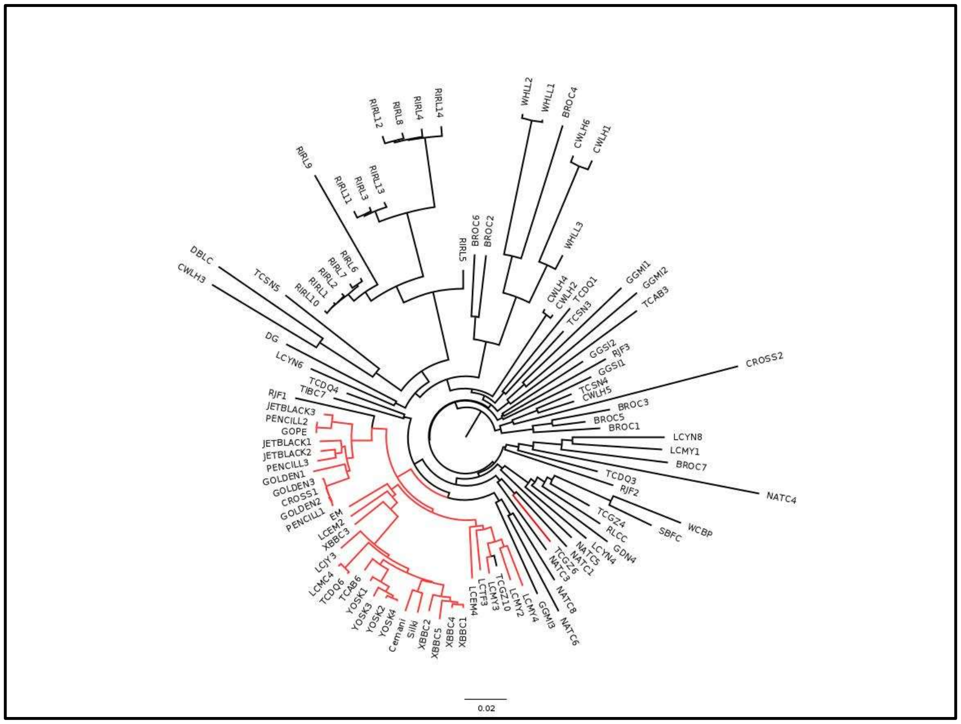

Supplementary Figure 47a. Phylogenetic relationship of Dup1 and Dup2 region for 101 individuals using a variant call from bcftools has been shown. Using vk phylo with nj method used for phylogenetic construction. Red color nodes represent the black-bone chickens, and black color nodes represent the non-black-bone chickens.

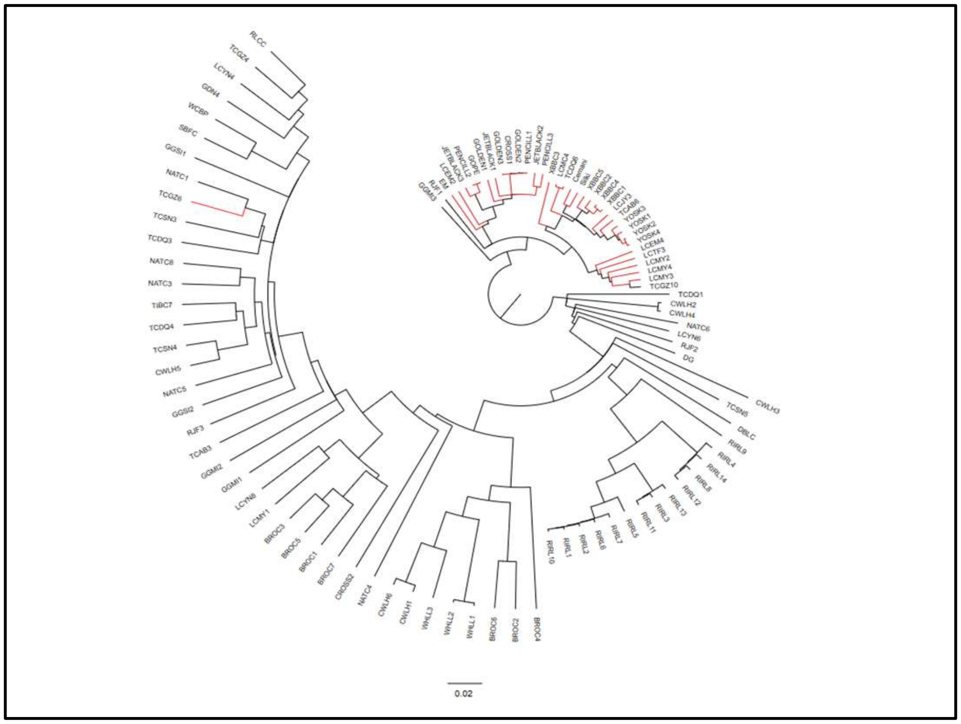

Supplementary Figure 47b. Phylogenetic relationship of Dup1 and Dup2 region for 101 individuals using a variant call from bcftools has been shown. Using vk phylo with UGPMA method used for phylogenetic construction. Red color nodes represent the black-bone chickens, and black color nodes represent the non-black-bone chickens.

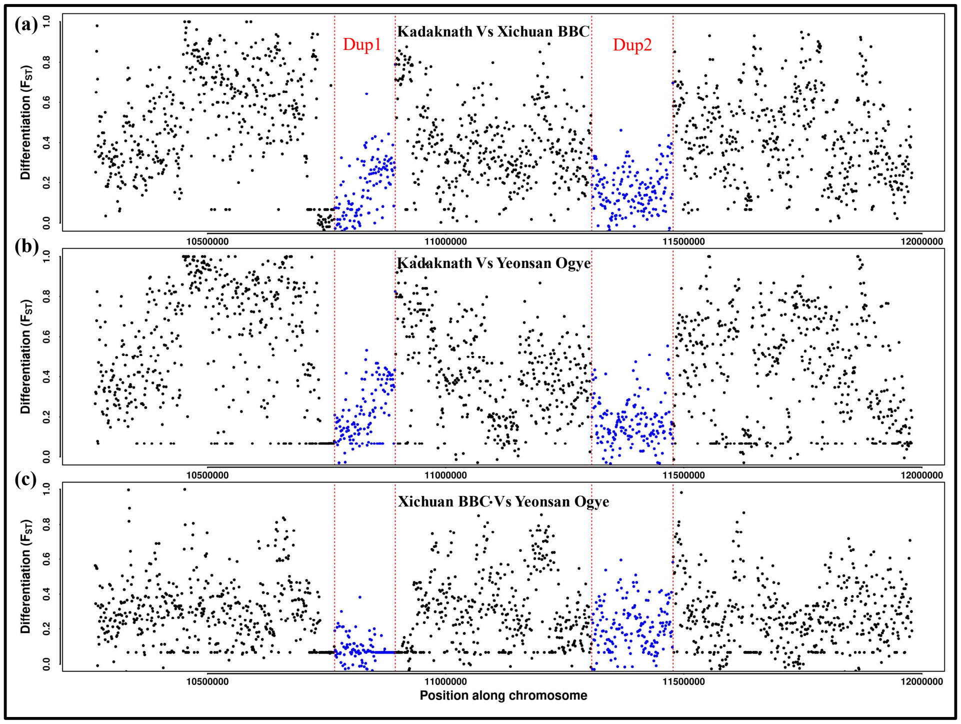

Supplementary Figure 48. Pairwise F_{ST} comparisons between black-bone populations: F_{ST} landscape of *Fm* locus between **(a)** Kadaknath and Xichuan BBC ,**(b)** Kadaknath BBC and Yeonsan Ogye and **(c)** Xichuan BBC and Yeonsan Ogye is shown in a 1Kb non-overlapping windows. Dup1 and Dup2 regions are represented in blue color dots.

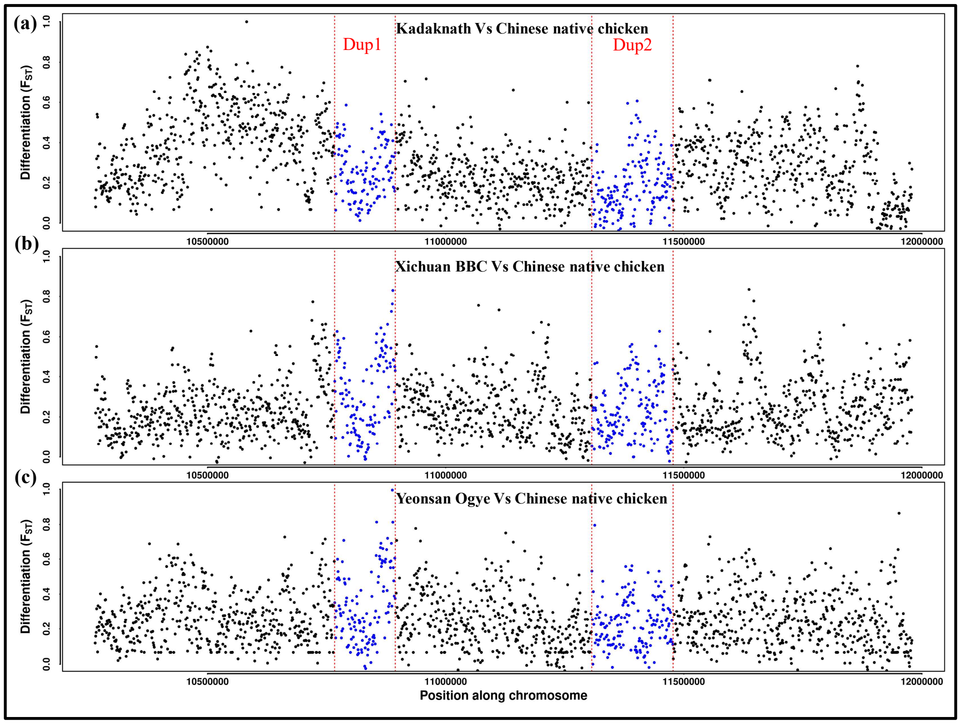

Supplementary Figure 49. Pairwise F_{ST} comparisons between black-bone and non-black-bone populations: F_{ST} landscape of *Fm* locus between **(a)** Kadaknath and Chinese native chicken,**(b)** Xichuan BBC and Chinese native chicken and **(c)** Yeonsan Ogye and Chinese native chicken is shown in a 1Kb non-overlapping windows. Dup1 and Dup2 regions are represented in blue color dots.

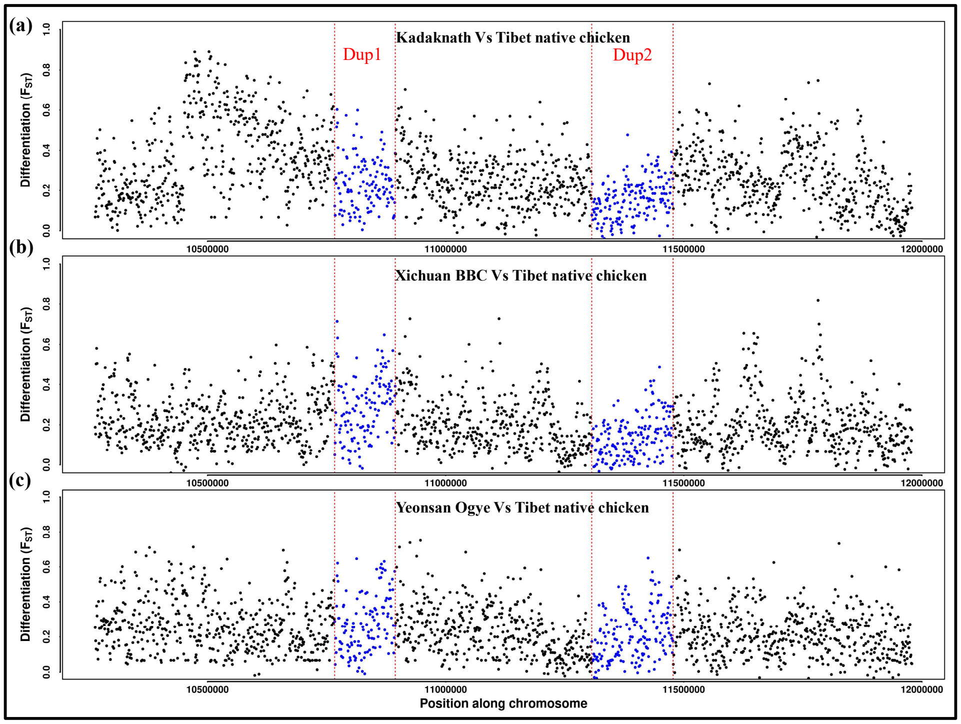

Supplementary Figure 50. Pairwise F_{ST} comparisons between black-bone and non-black-bone population: F_{ST} landscape of *Fm* locus between **(a)** Kadaknath and Tibet native chicken,**(b)** Xichuan BBC and Tibet native chicken and **(c)** Yeonsan Ogye and Tibet native chicken is shown in a 1Kb non-overlapping windows. Dup1 and Dup2 regions are represented in blue color dots.

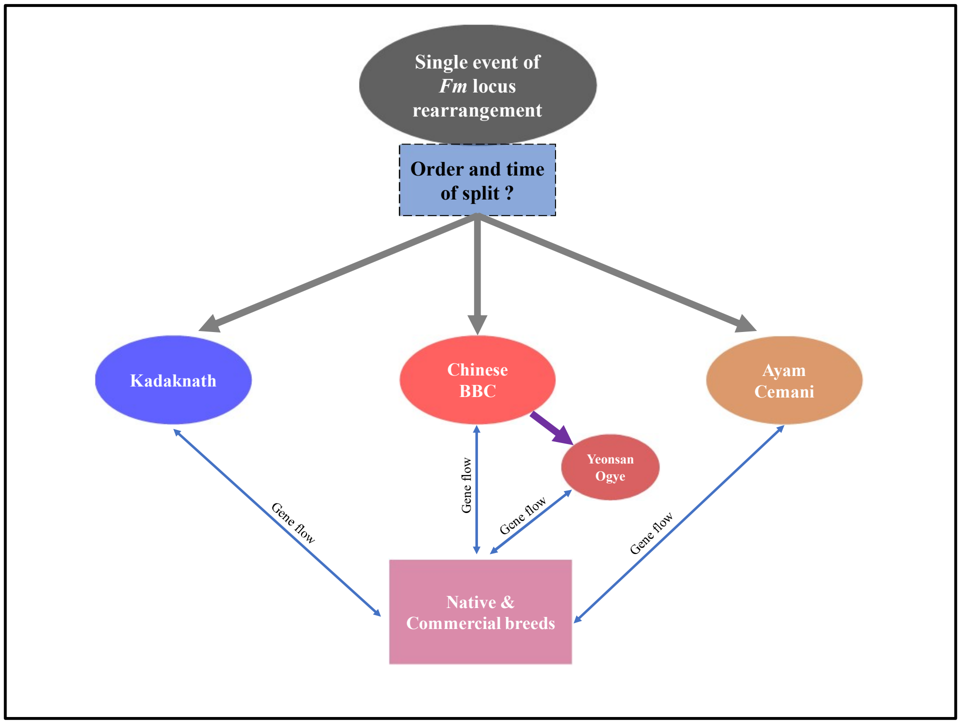

Supplementary Figure 51. The origin of black-bone phenotype (*Fm* locus rearrangement) and distribution of BBC breeds: The common origin of black-bone chicken through a single event at *Fm* locus rearrangement has been shown. The grey color arrows represent the distribution of different BBC breeds from a common ancestor. The purple color arrow represents the origin of Yeonsan Ogye from Chinese black-bone breeds. The two-sided blue color arrow represents the gene flow between different BBC breeds and various native and commercial breeds.

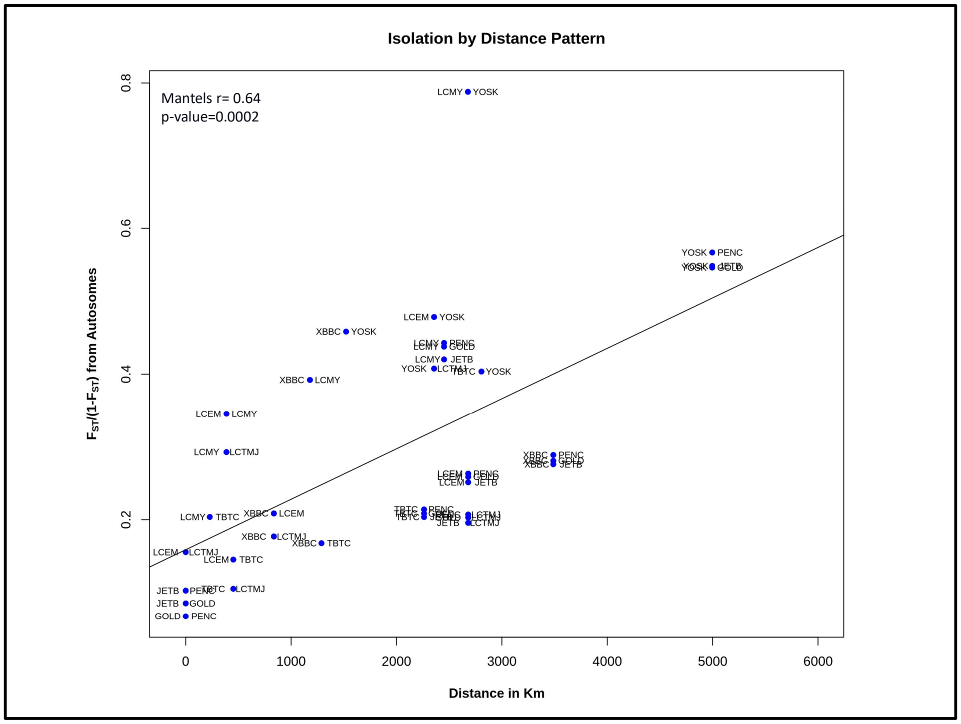

Supplementary Figure 52. Isolation by distance (IBD) pattern between 9 black-bone chicken populations: Pairwise mean F_{ST} comparison between 9 black-bone chicken populations (JETB, PENC, GOLD, XBBC, LCMY, LCEM, TBTC, YOSK, and LCTMJ) using ANGSD is shown at Y-axis, while the geographical distance in km is shown at X-axis.

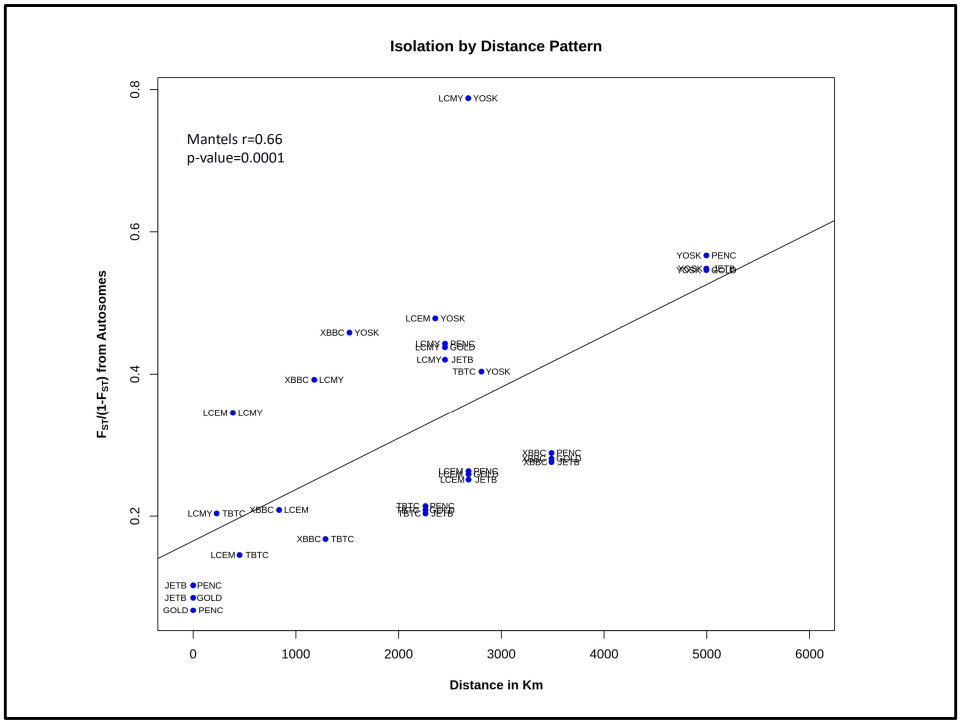

Supplementary Figure 53. Isolation by distance (IBD) pattern between 8 black-bone chicken populations: Pairwise mean F_{ST} comparison between 8 black-bone chicken populations (JETB, PENC, GOLD, XBBC, LCMY, LCEM, TBTC, and YOSK) using ANGSD is shown at Y-axis, while the geographical distance in km is shown at X-axis.

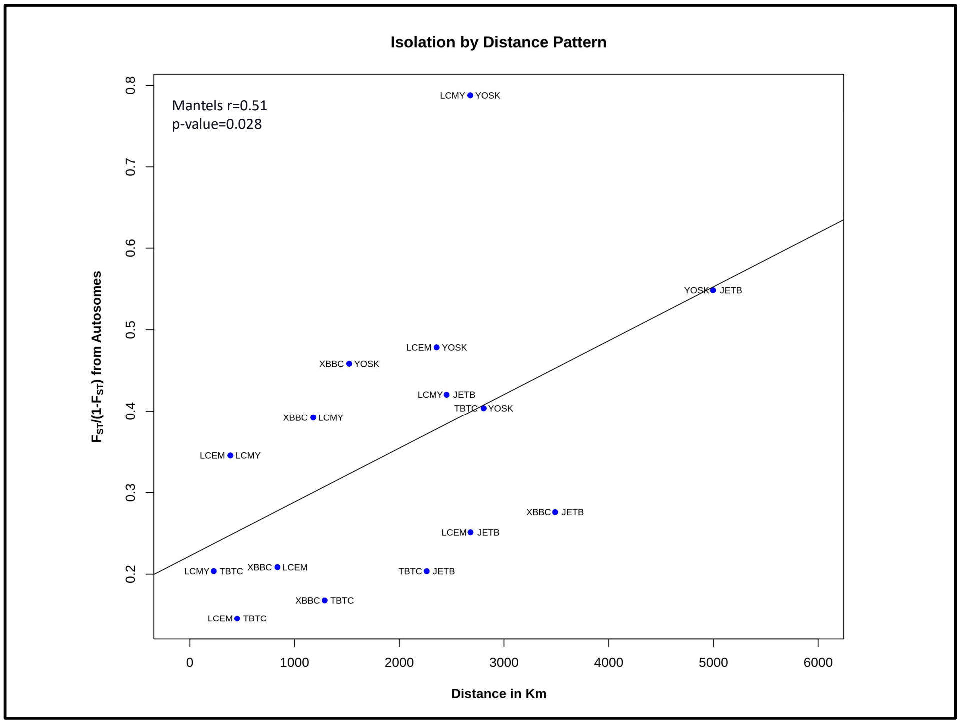

Supplementary Figure 54. Isolation by distance (IBD) pattern between 6 black-bone chicken populations: Pairwise mean F_{ST} comparison between 6 black bone-chicken populations (JETB, XBBC, LCMY, LCEM, TBTC, and YOSK) using ANGSD is shown at Y-axis, while the geographical distance in km is shown at X-axis.

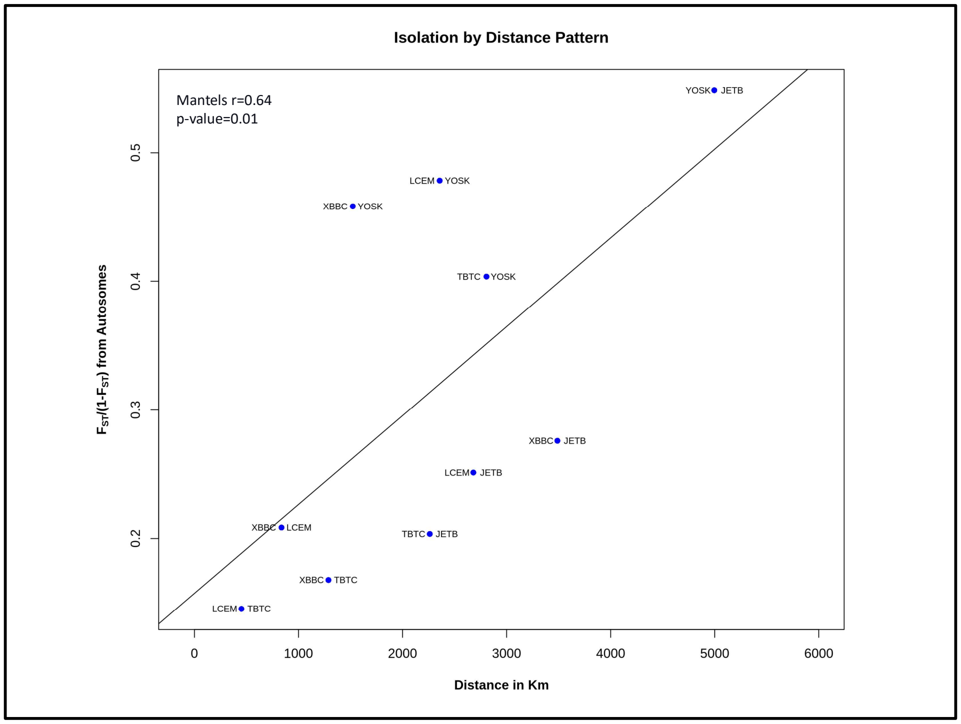

Supplementary Figure 55. Isolation by distance (IBD) pattern between 5 black-bone chicken populations: Pairwise mean F_{ST} comparison between 5 black-bone chicken populations (JETB, XBBC, LCEM, TBTC, and YOSK) using ANGSD is shown at Y-axis, while geographical distance in km is shown at X-axis.

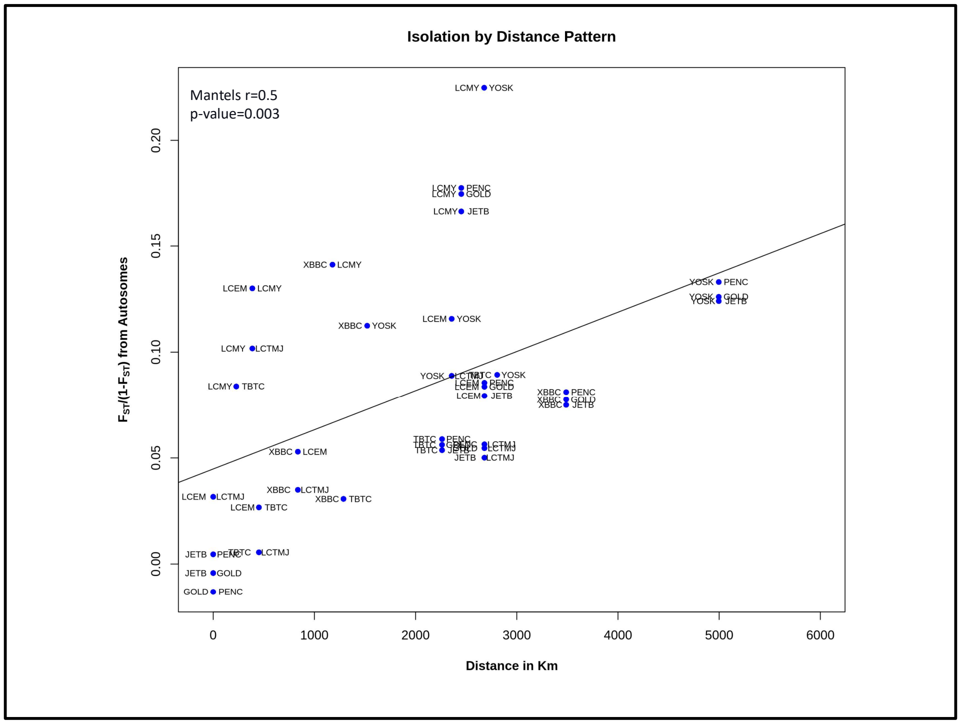

Supplementary Figure 56. Isolation by distance (IBD) pattern between 9 black-bone chicken populations : Pairwise mean F_{ST} comparison between 9 black bone-chicken populations (JETB, PENC, GOLD, XBBC, LCMY, LCEM, TBTC, LCTMJ, and YOSK) using popgenWindows.py is shown at Y-axis, while the geographical distance in km is shown at X-axis.

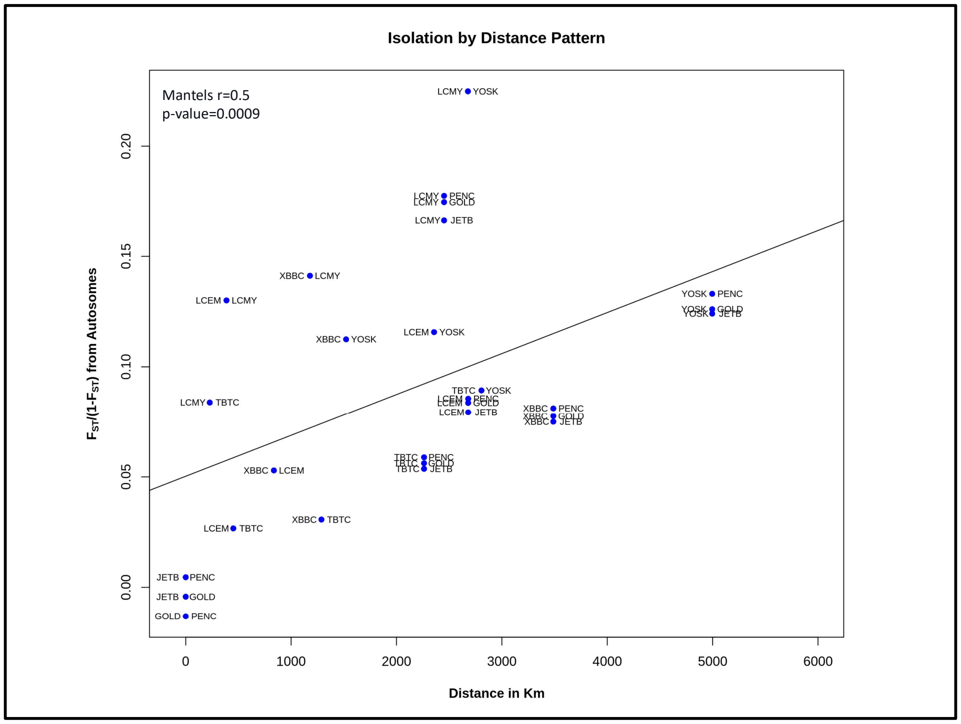

Supplementary Figure 57. Isolation by distance (IBD) pattern between 8 black-bone chicken populations : Pairwise mean F_{ST} comparison between 8 black-bone chicken populations (JETB, PENC, GOLD, XBBC, LCMY, LCEM, TBTC, and YOSK) using popgenWindows.py is shown at Y axis, while the geographical distance in km is shown at X axis.

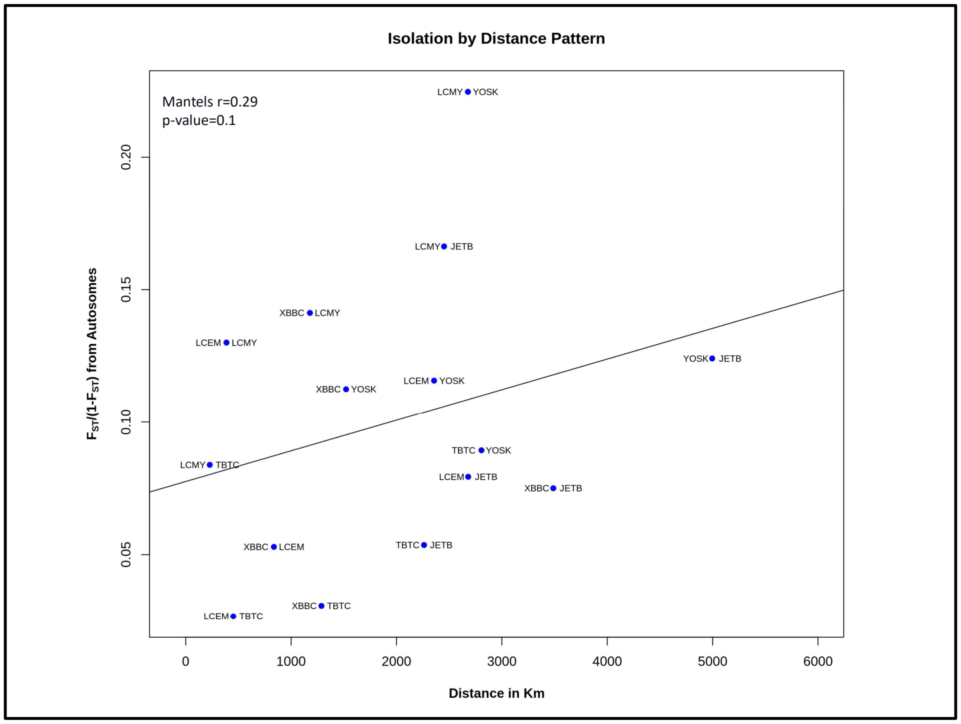

Supplementary Figure 58. Isolation by distance (IBD) pattern between 6 black-bone chicken populations : Pairwise mean F_{ST} comparison between 6 black-bone chicken populations (JETB, XBBC, LCMY, LCEM, TBTC, and YOSK) using popgenWindows.py is shown at Y-axis, while the geographical distance in km is shown at X-axis.

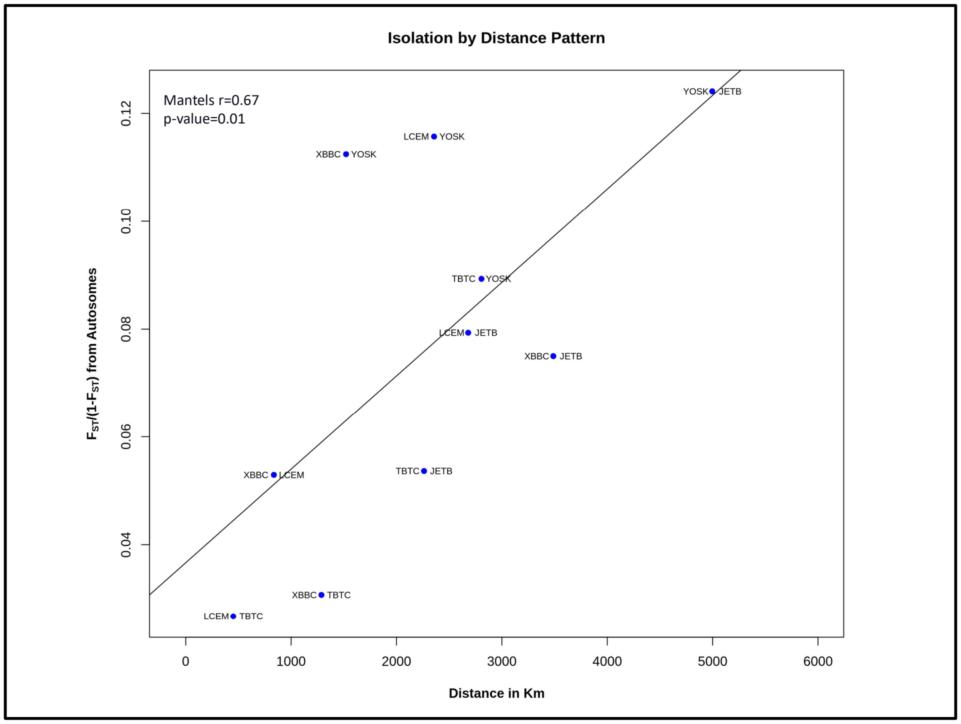

Supplementary Figure 59. Isolation by distance (IBD) pattern between 5 black-bone chicken populations: Pairwise mean F_{ST} comparison between 5 black-bone chicken populations (JETB, XBBC, LCEM, TBTC, and YOSK) using popgenWindows.py is shown at Y-axis, while the geographical distance in km is shown at X-axis.

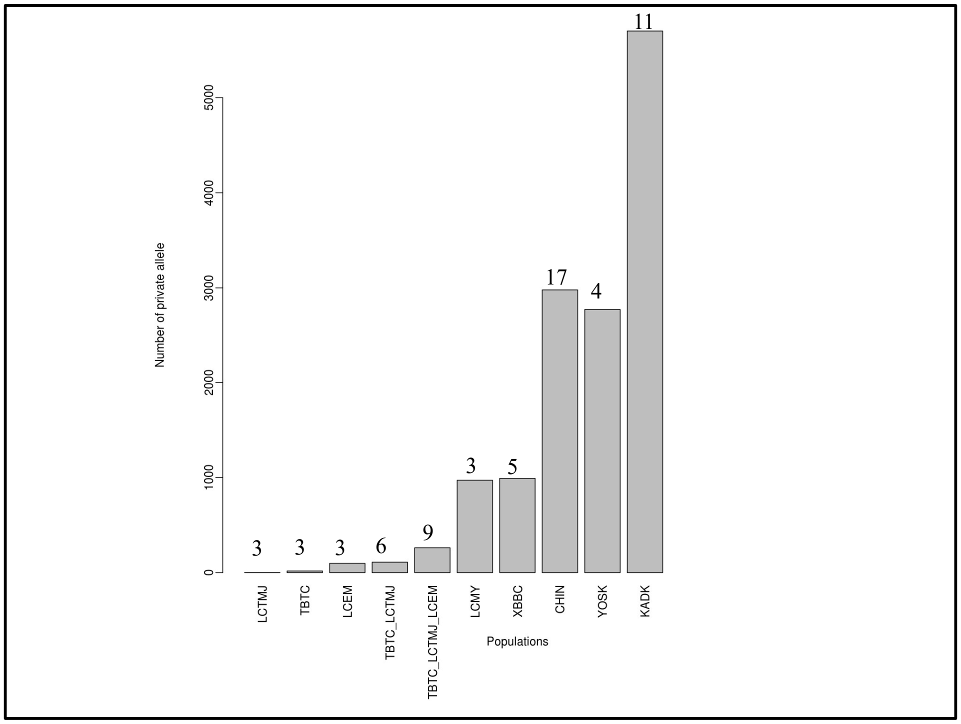

Supplementary Figure 60. Population-specific private allele count showed on Y-axis and X-axis represents the populations. The numbers of individuals in each population is mentioned at the top of each bar.

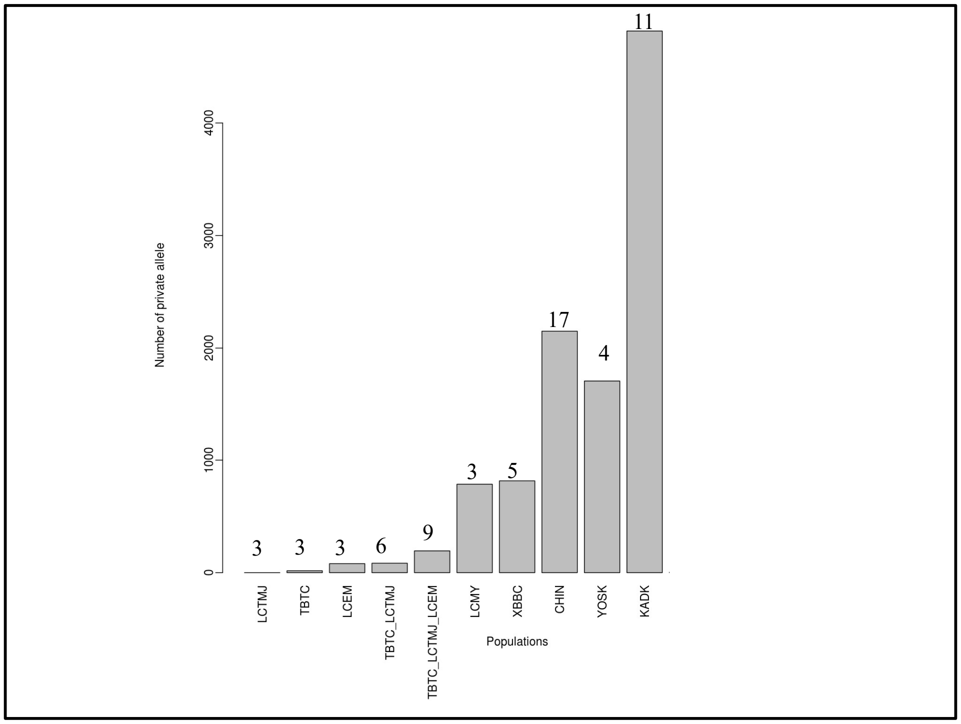

Supplementary Figure 61. Population-specific private allele count shown on Y-axis and X-axis represents the populations. The numbers of individuals in each population is mentioned at the top of each bar. The counts of population-specific private alleles are obtained after removing the common alleles from dbSNP.
